# Diversity Scale of Library Matters: Impact of mRNA Library Diversity Scales on the Discovery of Macrocyclic Peptides Targeting a Protein by the RaPID System

**DOI:** 10.1021/acscentsci.4c01021

**Published:** 2025-03-10

**Authors:** Jinxuan Zhao, Yi Li, Naohiro Terasaka, Haruo Aikawa, Hiroaki Suga

**Affiliations:** † Department of Chemistry, Graduate School of Science, The University of Tokyo, Bunkyo, Tokyo 113-0033, Japan; ‡ Earth-Life Science Institute, Tokyo Institute of Technology, Meguro, Tokyo 152-8550, Japan

## Abstract

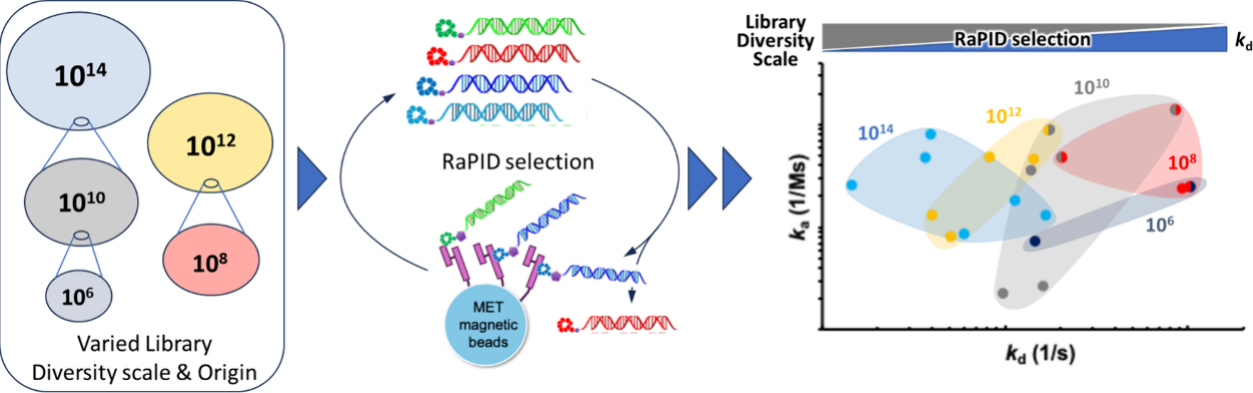

Macrocyclic peptides make up a unique class of modalities known for their high affinity, specificity, and ability to modulate protein–protein interactions, including receptor activation. Messenger RNA display, including the Random Nonstandard Peptides Integrated Discovery (RaPID) system, stands out in identifying target-specific macrocyclic peptides, producing potent binders with low to subnanomolar dissociation constants against diverse targets. It has often been discussed that this success is partly attributed to the vast library of over a trillion different peptide sequences expressed from the corresponding mRNA sequences. However, the impact of library scales on the identification of various binders has not been experimentally validated. Here, we report the RaPID selections against an ectodomain of a receptor tyrosine kinase MET using peptide libraries ranging from 10^6^ to 10^14^ unique members of mRNAs. We thoroughly analyzed the outcomes, including the binding kinetic properties, of the enriched peptide families. This study provides valuable guidelines for designing libraries with various numbers of sequences and selection conditions to enrich macrocyclic peptides with the desired characteristics.

## Abstract 2

The initial sampling of the sequence space determines the evolution of the families by the RaPID selection pressure for slow dissociation rates.

## Introduction

Peptide-based molecules are increasingly recognized as promising frameworks for drug development. Their structural diversity and extensive surface areas enable highly potent interactions with targets and offer remarkable specificity.^1,2^ Short peptides, comprising 4 to 20 amino acids, stand out from traditional drug modalities for their unique properties. They have better chemosynthetic accessibility than antibody drugs and show excellent potential for modulating protein–protein interactions, which was previously deemed “undruggable” due to the lack of suitable small molecule binding sites.^3−5^ These features are proven by the success of peptide therapeutics derived from naturally occurring peptide hormones^6^ and natural product-like peptides.^7^

Exploring new peptides has extended beyond naturally occurring compounds to *de novo* discovery from synthetic combinatorial peptide libraries, attracting attention from the pharmaceutical industry and academic research. In contrast to the isolation of bioactive natural peptidic products from organisms or bacterial lysates, selection methods identify “hit” peptides based on their affinity to a target protein from the peptide libraries that include a vast number of unique sequences to provide designated levels of diversity.^8^ Various “affinity-based” selection methods have been developed against the protein of interest to construct peptide libraries. The One-Bead-One-Compound (OBOC) library is prepared by chemical synthetic approaches of peptides, yielding a typical diversity of 10^5^–10^7^ compounds,^9^ and the more recent development of the DNA-encoded library (DEL) expands its “theoretical” scale to 10^9^ to be handled in industrial and academic laboratories.^10,11^ Biological displays, such as bacteria displays,^12^ yeast displays,^13^ and phage displays,^14^ employ the host translation system to realize the peptide expression with the 10^7^–10^9^ sequences, while *in vitro* displays, such as mRNA (mRNA) displays,^15^ utilize the *in vitro* translation system, giving the highest diversity scales of library over one trillion (10^12^–10^14^).

The dissociation constants (*K*_D_s) of the hit peptides generally range from high μM to sub-nM. As examples summarized in Supporting Table S1A–E, there is a trend that libraries with higher diversity scales have afforded stronger binders against a given target. Particularly, the recent advances in mRNA display combined with the genetic code reprogramming, *e.g*., the RaPID (Random nonstandard Peptides Integrated Discovery) system, have resulted in the discovery of potent *de novo* macrocyclic peptides containing exotic amino acids, such as *N*-methyl-α-amino acids, α-d-amino acids, and β/γ-amino acids, with *K*_D_s in the low nM range (Table S1A). Note that about 10^12^–10^14^ unique mRNA sequences constitute the library utilized in the RaPID system,^5^ underscoring the pivotal role of diversity scales of library in improving peptide screening.

Despite the apparent advantage of using a higher diversity of library in discovering potent peptides, the selection outcome is not solely dependent on the scale of library diversities,^16^ but still shaped by various other factors, such as library design,^17−19^ amino acid,^20^ technical reproducibility of method,^21^ and the expertise of the researchers in the laboratories. To the best of our knowledge, there has been no systematic exploration to address the influence of diversity scales of library to the binding properties of RaPID-derived macrocyclic peptides. Here, we ask simple questions: “Does diversity scale of library really matter?” and “How do the library diversity scales affect the selection results?” To address these questions, we have created macrocyclic peptide libraries with theoretical scales from 10^6^ to 10^14^ on purpose and performed the RaPID selection against MET (hepatocyte growth factor receptor). Analyzing the enrichment trends of sequences from each library, we discuss the impact of library scale on kinetic parameters and dissociation constants, confirming that higher diversity scales of the library help to identify binders with slower dissociation rates (*k*_d_) under the RaPID selection platform.

## Results

### Experiment Design and Construction of Macrocyclic Peptide Libraries with Varied Diversity Scales

To answer our questions raised in the introduction, we chose the ectodomain of MET fused to Fc as a selection target for two reasons. (1) Since we previously performed the RaPID campaign using a thioether-macrocyclic peptide library and successfully identified potent ligands with the range of *K*_D_ of 2–20 nM (Table S1A), this target has been validated as a reliable protein to reproduce the selection experiment.^22^ (2) At the time when the selection was performed previously, the NGS technology was unavailable in our laboratory, and thereby the sequences were determined by ∼40 clones available from the *E. coli* transformation; therefore, the evolution of enriched sequences was not studied. One of the potent macrocycles found in the study was 17-mer aML5 (*K*_D_ = 19 nM), which can be used as a benchmark for the present study.

**Figure 1:**
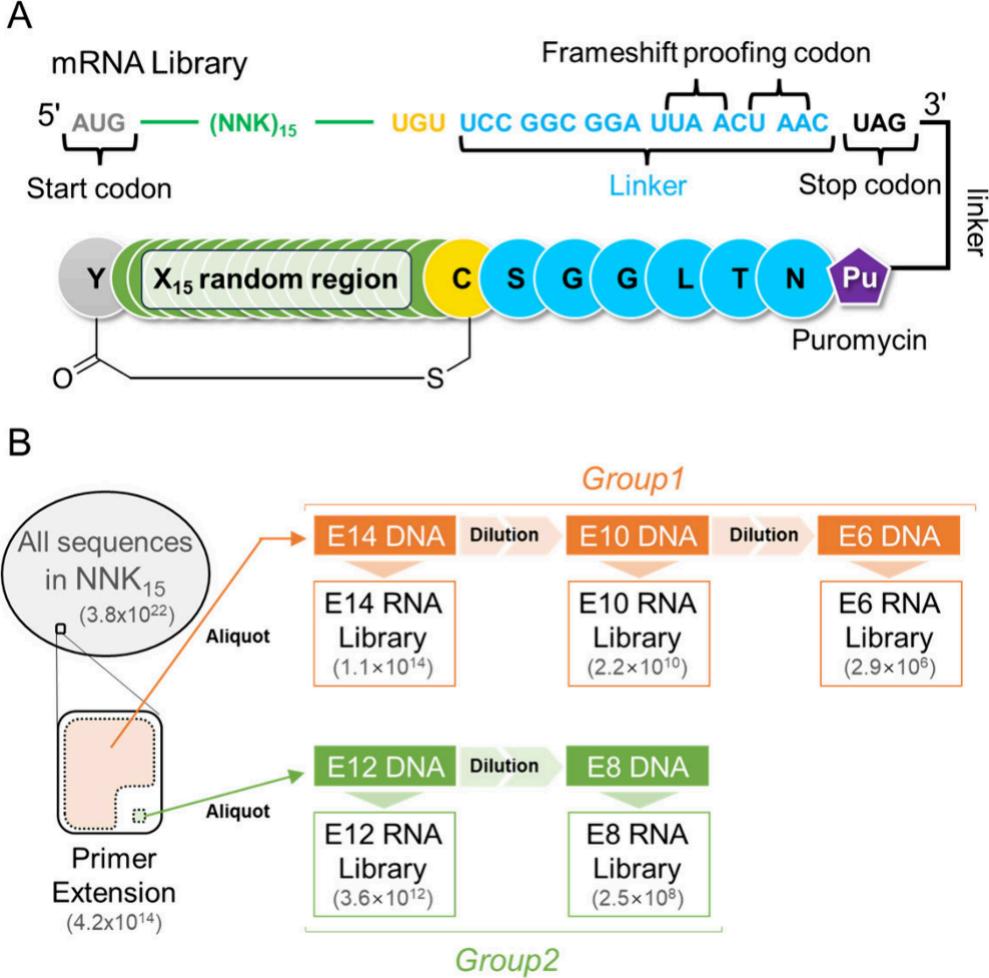
Design of peptide-mRNA libraries and library preparation of various diversity scales. (A) The structure of peptide-mRNA. Peptide is connected to its mRNA via puromycin in the translation step. (B) Schematic representation of the preparation of each library. Group 1 consists of E14, E10, and E6. Group 2 consists of E12 and E8. The estimated sequence diversity of each library is shown in parentheses. Each DNA library was transcribed into the mRNA library and then subjected to translation to produce an mRNA display library based on the RaPID protocols for reprogramming of the initiation codon to ClAc-^L^Tyr for macrocyclization with Cys’s side chain at the downstream position(s) as shown in Figure 1A.

We prepared a macrocyclic peptide library as follows (Figure 1A): (1) for the peptide region, 5′-AUG-(NNK)_15_-UGU-3′ was used as the mRNA template of random peptide sequences, where the initiator AUG codes ClAc-^L^Tyr and UGU codes Cys for cyclization, (2) a Ser-Gly-Gly-Leu-Thr-Asn linker sequence (UCC-GGC-GGA-UUA-ACU-AAC) was added in the downstream of the macrocyclic peptide sequence, designed to halt translation via a UAA stop codon in case that a frameshift would occur, and (3) all mRNA were ligated to the puromycin (DNA-PEG-CC-Pu linker). In the release factor I-deficient (RF1^–^) RaPID system, the stalling of the ribosome at the UAG stop codon on the mRNA generates mRNA-peptide conjugation via the incorporation of puromycin into the peptide chain during the translation event.

To construct the mRNA library of the desired diversity scale, a subset of the NNK_15_ DNA library, theoretically containing a maximum of 3.8 × 10^22^ different sequences, was first prepared by the primer extension and PCR. Mother libraries with a DNA diversity of approximately 10^14^ and 10^12^, named E14 and E12, respectively, were aliquoted from the resulting library subsets (Figure 1B). Since they were independently sampled from the original NNK_15_ library, where only one copy of the respective sequences existed, they theoretically contained unique sequence spaces. A small fraction was then taken from the library and diluted by 4 orders of magnitude into each daughter library subset, E10 from E14, E6 from E10, and E8 from E12. The diversity scale of each DNA library (E14, E12, E10, E8, and E6 libraries) was calculated to be 1.1 × 10^14^, 3.6 × 10^12^, 2.2 × 10^10^, 2.5 × 10^8^, and 2.9 × 10^6^, respectively. Each DNA library was then transcribed *in vitro* into the mRNA library. The quality of each library was verified by gel electrophoresis of mRNA and next-generation sequencing of reverse-transcribed DNA samples (Figure S1). We categorized these libraries into two groups based on their origin; E14, E10, and E6 libraries belong to Group 1, and E12 and E8 libraries belong to Group 2 (Figure 1B).

### The RaPID Selection of Macrocyclic Peptide Binders Targeting MET

The RaPID system was employed to identify anti-MET macrocyclic peptides as previously described.^15^ It should be noted that each ribosome is only available once in the system due to stalling of the ribosome at the UAG stop codon on the mRNA, followed by the formation of the mRNA-peptide fusion. Therefore, the number of mRNA molecules added to the *in vitro* translation system needs to be equal to that of ribosomes, and each unique mRNA sequence forms a single molecule of mRNA-peptide fusion. The ribosome concentration in the *in vitro* translation system is 1.2 pmol/μL (7.2 × 10^11^ ribosomes per μL), so the translation scale was manipulated to cover the expected diversity. For the E14 library, 150 μL scale translation containing 1.1 × 10^14^ ribosomes was performed to express the macrocyclic peptides from the 180 pmol of mRNA. We calculated the sequence diversity based on the assumption where the ribosome does not act as a polysome on the mRNA but rather acts as a monosome in our system due to the short length of the mRNA consisting of ∼100 nucleotides available for ribosome binding.^23,24^ For the E12 library, a 5 μL scale translation containing 3.6 × 10^12^ ribosomes was conducted using 6 pmol of mRNA. For the libraries with smaller diversity scales (<10^10^), we fixed the translation scale to 2.5 μL containing 3 pmol of mRNA and 1.8 × 10^12^ ribosomes. The recovery rates in round 1 of this study were in the range of 0.0005 to 0.002%, meaning the sequence diversity was suppressed by approximately 2 × 10^5^-fold or more (Figure S3), making it possible to cover the diversity scale of library in round 2 or later rounds by 2.5 μL scale translation.

The RaPID selection against MET using each mRNA library was conducted as follows (Figure S2): (1) mRNA was ligated with Pu-linker, (2) the mRNA library was *in vitro* translated to form peptide-mRNA, (3) the peptide-mRNA fusion library was reverse-transcribed to form the corresponding peptide-mRNA/cDNA complex library, (4) the resulting library was then subjected to the negative beads containing a mixture of naked and the Fc-tag-immobilized magnetic beads, for 1 time (typically 6 times in other practices) to remove nonspecific or Fc-tag binders, (5) the supernatant was applied to Fc-tagged MET-immobilized magnetic beads (positive beads) to recover cDNA of MET-binding macrocyclic peptides, and (6) the cDNA was amplified by PCR, and then transcribed into the mRNA library for the subsequent selection round.

By repeating the above affinity selection, the recovery rate, *i.e*., the ratio of the amount of cDNA recovered from the MET-immobilized positive beads compared to the total amount of cDNA in the input library, began to increase at round 3 or later. For E10, E12, and E14 libraries, the increase in recovery rate became apparent at round 4 and plateaued by round 7 (Figure S3). For the E6 and E8 libraries, the recovery rate did not dramatically ramp up at round 4 (especially for E6) but continued to increase until round 7. Theoretically, the recovery rate in the positive selection increases as the molecules with high affinity are enriched. Thus, the earlier increase in positive selection recovery in the E10, E12, and E14 libraries would indicate the presence of molecules with higher affinity in the higher diversity scales of the library.

We subsequently deep-sequenced the recovered cDNA of all libraries from rounds 1 to 7 (Supporting Information 2 NCBI Bioproject ID: PRJNA1151868 reporting the full NGS data). In our previous anti-MET selection that yielded aML5 and aMD4, we employed a classical method of DNA cloning and Sanger sequencing.^22^ In the current study, we employed next-generation sequencing (NGS) without DNA cloning bias, thus providing more sequence information on positive clones that appeared in the enriched libraries and deeper insights into the population and its changing during the selection.^25^ The NGS output of each library was analyzed to determine the positive clones for the following characterization.

### Synthesis and *In Vitro* Binding Affinity Analysis of Selected Anti-MET Macrocyclic Peptides

Peptides with a read number exceeding 2% of the total reads in each library (*i.e*., the “triaged criterion” is less than 2%) were chosen as positive clones (Table S3). For the libraries that possessed more than 6 positive sequences, the top 6 or 7 sequences were selected. Using this criterion, 23 macrocyclic peptides, including 4 pairs of identical sequences (6–1 and 8–4; 8–1 and 10–2; 8–2 and 10–3; 10–1 and 12–3), were successfully prepared by Fmoc solid-phase peptide synthesis (Table 1). Here, it should be noted that we observed more identical sequences across groups when we extended the analysis to the top 20 sequences in each library, which might indicate that the sequence pools were nested or that mutations occurred under the selection conditions. The purity and molecular weight of the peptides were confirmed by ultraperformance liquid chromatography (UPLC) and MALDI-TOF-MS, respectively (Figure S6 and S7). For 10–4 and 12–4 containing three Cys residues, the mutants with two of the Cys replaced by Ser were *in vitro* translated and analyzed based on the recovery rates from MET-immobilized beads to identify potent mutants (Figure S4). These experiments revealed that the third and the first Cys were used for cyclization for 10–4 and 12–4, respectively, and these mutants were synthesized upon the Cys with different protecting groups to form a thioether macrocycle on the desired Cys residue (Supporting Information). The mass spectroscopy data indicated that two proximal Cys residues of 10–4 were in free thiol form without oxidation, whereas the two distal Cys residues of 12–4 were in disulfide form, yielding a peptide with a dumbbell-type bicyclic structure.

**Table 1:**
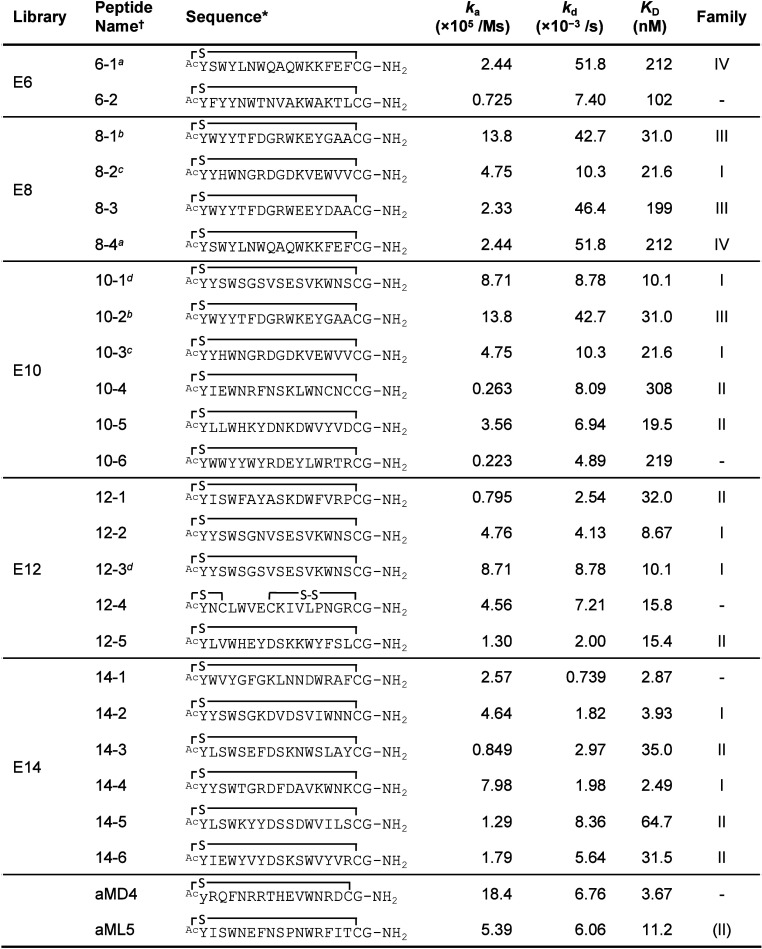
Sequence, Binding Kinetic Indexes, and Family of the Macrocyclic Peptides Obtained from Libraries with Different Diversity Scales of Libraries

* Cyclization structure: “–S–” stands for the thioether formed between *N*-acetyl and thiol of cysteine; “–S–S–” stands for the disulfide bond between two cysteines; “y” in the aMD4 stands for the DTyr.

† Same sequence: ^*a*^6–1 and 8–4; ^*b*^8–1 and 10–2; ^*c*^8–2 and 10–3; ^*d*^10–1 and 12–3.

The binding kinetic constants (*k*_a_ and *k*_d_) and dissociation constant (*K*_D_) of each macrocyclic peptide were determined by surface plasmon resonance, SPR (Table 1, Figure S8). The SPR data of all peptides showed a typical concentration-dependent sensorgram and were fitted by a 1:1 binding model. The calculated *K*_D_ varied over 2 orders of magnitude, from 3 nM to 300 nM. The measurement of *K*_D_ values of aMD4 and aML5 were reexamined and determined to be 3.7 and 11.2 nM, respectively, which are close to the previously determined values.^22^ Since our previous thorough studies on aMD4 and aML5 showed high specificity for MET,^22^ the peptides with low *K*_D_ values (<50 nM, Table 1) are most likely specific for MET. Importantly, we observed a rough correlation between the *K*_D_ values and the subset library’s diversity scale (Table 1), where peptides in the smaller scale libraries (E6 and E8) exhibited relatively larger *K*_D_ values, and those with single-digit *K*_D_ values were mainly found in higher diversities scale of libraries (E12 and E14). Based on the previous RaPID selection results, the most abundant sequences in each library do not always have the lowest *K*_D_ value in the family, presumably due to other factors such as PCR amplification bias and/or translation efficiency bias.

**Figure 2:**
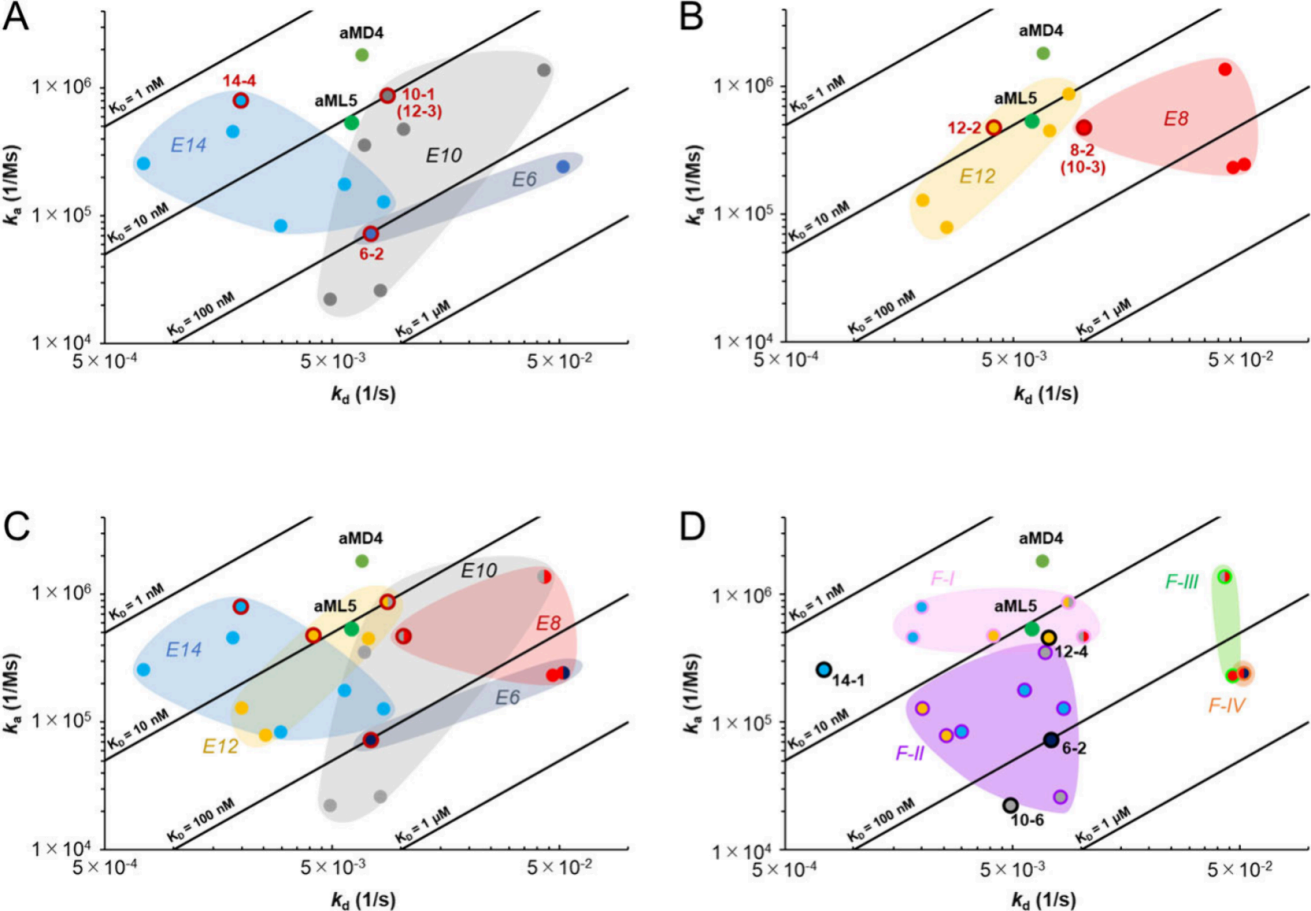
Scatter plots of hit peptides analyzed on kinetic parameters (*k*_a_ and *k*_d_) binding to MET protein. (A–C) Color codes are used for each library as follows: Blue for E14, yellow for E12, gray for E10, red for E8, and dark blue for E6 and their corresponding peptides. Dots with dark red outer frames are the best binders, with the smallest *K*_D_ in each library. (A) Plot of the Group 1 libraries, E14, E10, and E6. (B) Plot of the Group 2 libraries, E12 and E8. (C) The merged plots of both Group 1 and 2. (D) Plot of peptides representing families with common sequences. The frame color of each dot represents the family to which the peptide belongs: pink, purple, light green, and orange for Family I (F–I), F–II, F–III, and F–IV, respectively. Four unique sequences that do not belong to any peptide family are not included in the color block and are marked with black outer frames.

To visualize the trend of the kinetic behavior of the peptides, we plotted the dissociation rate (*k*_d_) on the *x*-axis against the association rate (*k*_a_) on the *y*-axis (Figure 2). In this plot, the clones in the upper left area represent the most potent molecules. In the Group 1 libraries, hit peptides from the E14, E10, and E6 libraries occupied different areas of the plot, reflecting the trends in *k*_a_ and *k*_d_ (Figure 2A). First, the range of *k*_a_ of the hit peptides was about 2 × 10^4^–2 × 10^6^ M^–1^ s^–1^ and did not vary significantly with library diversity scales. Second, most of the hit peptides in E14 have lower *k*_d_ values than those of hit peptides from E10 or E6, indicating some advantage of library diversity scales in discovering those with lower *k*_d_ values. To see the generality of the above trends, we analyzed the kinetic properties of hit peptides in the Group 2 libraries (E12 and E8), which have a different “sampling origin” of mRNA from Group 1 (Figure 2B). Interestingly, similar trends were reproduced, and the library diversity scale clearly affected the *k*_d_ values of hit peptides. These analyses indicate that RaPID selection under the conditions used here was mainly a *k*_d_-dependent process, although this is inconclusive across the groups from the merged plot (Figure 2C).

Next, we tried to find high-affinity motifs from the collection of peptides identified from all of the libraries. The sequence alignment of these 19 clones (23 peptides were selected with four duplicates) revealed four peptide families (F–I–IV, F stands for Family; Table 2), where each family has a clear consensus motif. However, four unique binders, 6–2, 10–6, 12–4, and 14–1, could not be assigned to any of the families found in most of the libraries. These peptide families and unique sequences indicated that characteristic consensus sequences were identified, regardless of the sampling scales from the initial NNK library. Each family and its peptide components are shown in Figure 2D, and the average binding parameters of each family, *k*_aavg,_*k*_davg_, and *K*_Davg_, were calculated (Table 2).

**Table 2:**
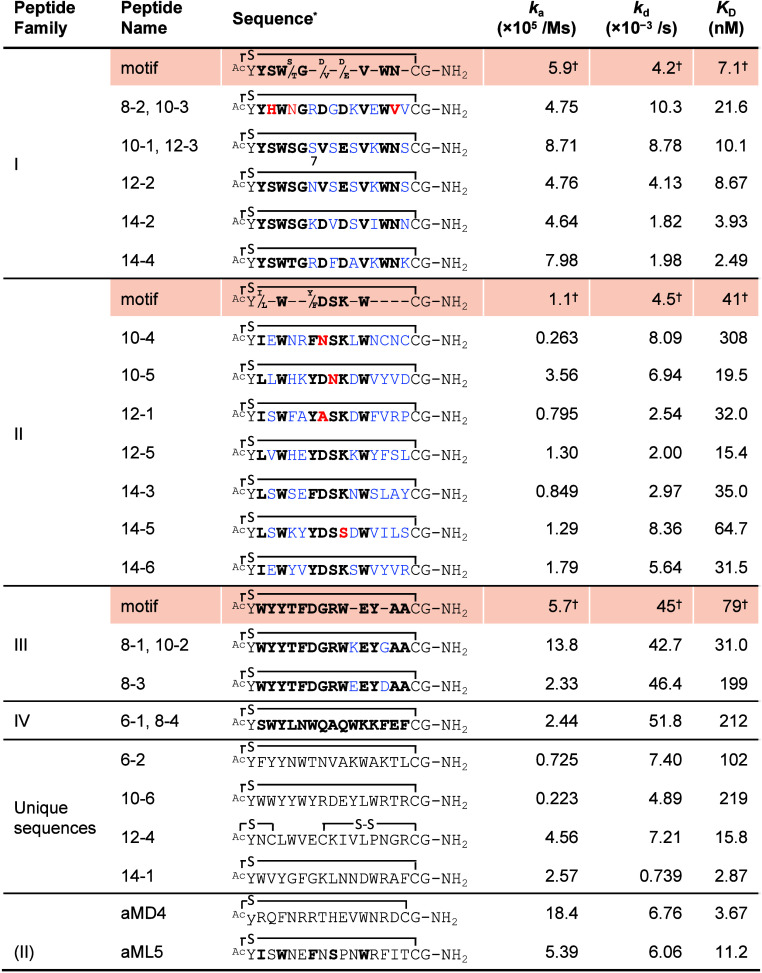
Sequence Alignment of Peptides Categorized by Common Sequences

* Cyclization structure: “–S–” stands for the thioether formed between the *N*-acetyl and the thiol of cysteine; “–S–S–” stands for the disulfide bond between two cysteines; “y” in the aMD4 stands for the DTyr; consensus residues in each sequence are highlighted with bold font; the residue that does not belong to the consensus residue is highlighted in blue; the residue which is not consistent with the consensus residue is highlighted in red.

† *k*_a_, *k*_d_, and *K*_D_ of consensus motif: The *k*_aavg_, *k*_davg_, and *K*_Davg_ are the average values of those of peptides in the family calculated in the logarithm scale.

Five peptides, including the 4 best binders of the E8–E14 library belong to the F–I (Table 2), cyclic(^Ac^Y**YSW**(**S/T**)**G**X(**D/V)**X(**D/E**)X**V**X**WN**XC)G-NH_2_ (the thiol group of the C-terminal Cys residue involved in thioether macrocyclization with the N-terminal Ac group, where the underlined bold residues are consensus), and the family showed *K*_Davg_ = 7.1 nM, *k*_aavg_ = 5.9 × 10^5^ M^–1^ s^–1^, and *k*_davg_ = 4.2 × 10^–3^ s^–1^ against the target protein. The consensus sequence of the F–I peptides most likely plays a critical role in the interaction with the MET.

The F–II, cyclic(^Ac^Y(**I/L**)X**W**XX(**Y/F**)**DSK**X**W**XXXXC)G-NH_2_, included the 7 peptides from the E10 to E14 libraries with *K*_Davg_ of 41 nM, *k*_aavg_ of 1.1 × 10^5^ M^–1^ s^–1^, and *k*_davg_ of 4.5 × 10^–3^ s^–1^ (Table 2). It is worth mentioning that the previously reported 17-mer peptide aML5, cyclic(^Ac^Y**I**S**W**NE**F**N**S**PN**W**RFITC)G-NH_2_ (*K*_D_ = 11.2 nM)^22^ shares sequence homology with the F–II, where the underlined bold residues are consensus. The reproducible discovery of F–II peptides in the different selection experiments indicates that this consensus sequence also represents a crucial motif for binding with a *k*_d_ value similar to that of F–I. However, their *k*_a_ values are generally slower than those of F–I.

For F–III peptides (*K*_Davg_ of 79 nM, *k*_aavg_ of 5.7 × 10^5^ M^–1^ s^–1^, and *k*_davg_ of 45 × 10^–3^ s^–1^) and IV peptide (*K*_D_ of 212 nM, *k*_a_ of 2.4 × 10^5^ M^–1^ s^–1^, and *k*_d_ of 52 × 10^–3^ s^–1^), they only contained the clones from the less diverse libraries E6–E10. Since the sequences are nearly the same among F–III peptides and F–IV peptides, it is difficult to extract useful information from the sequence alignment because the variation of these peptides is very limited (Table 2). In any case, the *k*_davg_ of F–I–IV would indicate that F–III and F–IV peptides with faster *k*_davg_ values were replaced by F–I and F–II peptides with slower *k*_davg_ as the library diversity scale expanded.

### Sequence Analysis of Macrocycles in Different Library Diversity Scales

To elucidate the enrichment of sequences during the selection process, we meticulously tracked the read counts for all peptide families and individual sequences of standout binders within the deep-sequencing data from each selection round across all libraries (Table S4). Since the total read numbers of each library were about 10^5^ in the NGS analysis, a read number of 0 represents an abundance below 0.001% in the library, which is considered negligible for subsequent analysis. By visualizing the population (read number) of each peptide family and individual sequences, a trend became apparent that the expansion of the mRNA library’s diversity scale introduced more potent unique peptides, discussed in detail below, and diminished the prevalence of the weak binders, especially F–III and F–IV peptides.

**Figure 3:**
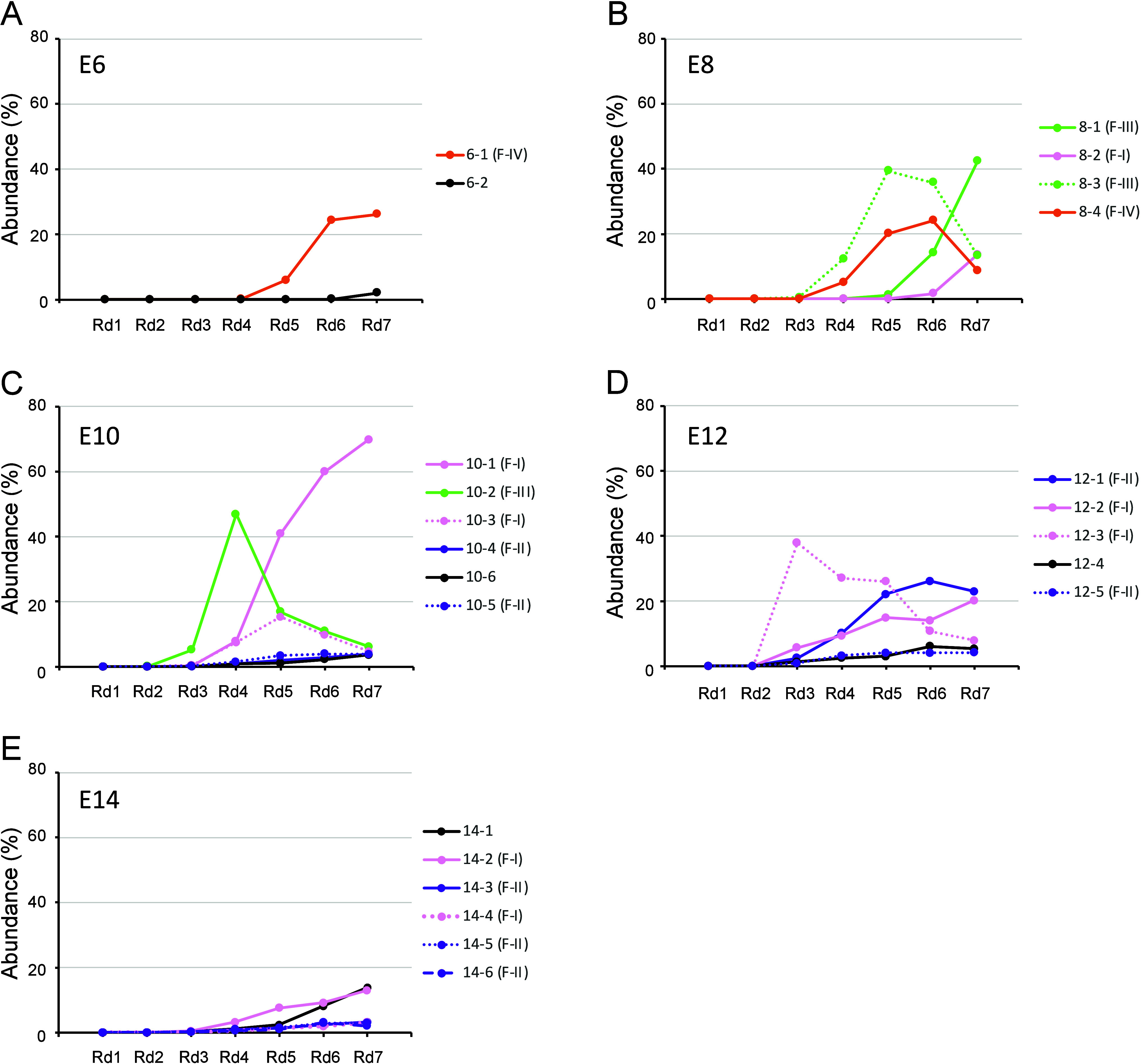
Transition of the population of hit peptides after each selection round. The abundance of each hit peptide was analyzed for the (A) E6, (B) E8, (C) E10, (D) E12, and (E) E14 library. Pink, purple, green, and orange color codes are used for F–I, F–II, F–III, and F–IV, respectively.

First, we analyzed the population dynamics of the peptide families in each round across the libraries (Figure S5; see also Table S4). Intriguingly, F–IV, identified as a single and the weakest species among all families, remained in the later rounds of the E6 and E8 libraries (Figure S5A and B), whereas it was completely depleted in the earlier rounds of the E10–14 libraries (Figure S5C–E). The E6 library yielded only the F–IV peptide (Figure S5A), with only a trace (0.03%) of F–II found in round 7. Although the F–IV also survived in the E8 library, yielding about 9% of the total population in round 7, it could not survive in E10 and larger libraries. Similarly, F–III, the second weakest species among the families, survived in the E8 and E10 libraries (Figure S5B, C), dominated in the E8 library in round 7, but became a minor species in the E10 library. Again, this family was nearly depleted in the E12 and E14 libraries as the library diversity scales expanded (Figure S5D and E). Conversely, F–I appeared in the E8–14 libraries, became a dominant species in the E10 library, and was maintained as a major species in the E12 and E14 libraries (Figure S5D and E). Meanwhile, F–II appeared only in the E10 and E14 libraries (not in E8) and competed with F–I as the dominant species. Thus, the increasing library diversity scales (such as E12 and E14 libraries) generally led to the displacement of the weaker family species (F–III and F–IV) by the stronger family species (F–I and F–II) due to slower *k*_d_ values. Although these trends summarized above are just the general observations for the families, mutant or unique potent species were involved in the competitions of each round, providing more meaningful insights into the surviving species. Next, we analyzed the population and kinetic data of individual peptides in each library (Figure 3 and Table S4).

In the E6 library, the population of peptide 6–1 (belonging to F–IV) started to increase in round 5 and plateaued in round 6 to possess a population of 26% in round 7 (Figure 3A). Notably, the sequence population analysis suggests the presence of unique peptide 6–2 and F–II peptides in the library (Table S4A). Although 6–2 has a 7-fold slower *k*_d_ (= 7.4 × 10^–3^ s^–1^) compared to 6–1 (*k*_d_ = 51.8 × 10^–3^ s^–1^, Table 2), it was not enriched and dominated in the library in round 7. We assume that the slower *k*_a_ property of 6–2 (*k*_a_ = 0.725 × 10^5^ M^–1^ s^–1^) than 6–1 (*k*_a_ = 2.44 × 10^5^ M^–1^ s^–1^) could compensate for the marginal difference in their *k*_d_ values and therefore 6–1 remained as the dominant species.

In the E8 library, 8–3 (F–III) and 8–4 (F–IV) rapidly emerged in early rounds (∼round 4), while 8–1 (F–III) and 8–2 (F–I) caught up in later rounds (Figure 3B and Table S4B). Peptide 8–2 shows a modest yet 5-fold slower *k*_d_ (= 10.3 × 10^–3^ s^–1^) than other species (*k*_d_ = ∼50 × 10^–3^ s^–1^, Table 1). This suggests that even though 8–2 was undetectable in earlier rounds by the triaged criteria, likely due to a low initial population possibly caused by PCR amplification bias, this species was enriched in the later rounds because of its slower *k*_d_. The rapid population increase of 8–1 (F–III) from negligible early counts to dominance in round 7, along with the decline of 8–3, illustrates the critical role of kinetic rates in determining peptide survival. The difference in *k*_d_ between these two F–III clones was very small (*k*_d_ = 42.7 vs 46.4 × 10^–3^ s^–1^, Table 2), but the slower *k*_a_ of 8–3 (= 2.33 × 10^5^ M^–1^ s^–1^) was likely responsible for its replacement by the faster *k*_a_ of 8–1 (= 13.8 × 10^5^ M^–1^ s^–1^). Note that the sequence differences in the peptide between 8-3 and 8–1 are two residues, E11K and D14G, where the mutations could occur at the mRNA codon sequence level, *i.e*., from GAG to AAG and from GAU to GGU in the most abundant sequence at round 7. This suggests that 8–3 in the initial E8 library might be mutagenized by PCR to yield the 8–1 sequence, resulting in the replacement of the 8–3 population with 8–1 (Figure 3B).

In the E10 library, 10–2 (F–III) was enriched in earlier rounds and became the major species in round 4. Accumulation of 10–2 in the earlier rounds likely occurred due to its faster *k*_a_. However, as the F-1 peptides 10–1 and 10–3, characterized by their slower *k*_d_ (= ∼10 × 10^–3^ s^–1^), increased in population, the prominence of F–III and F–IV decreased gradually (Figure S5C). In round 7, 10–1 occupied 70% of the total population and suppressed those of all other families (Figure 3C, S5C, and Table S4C). We also noticed that this library yielded all peptides belonging to F–I–III and a modest unique peptide 10–6, while the weakest F–IV was depleted in the later rounds.

In the E12 library, 12–1 (F–II) was enriched throughout the selection rounds, presumably due to its slow *k*_d_ (= 2.54 × 10^–3^ s^–1^, Figure 3D and Table S4D), while 12–5 (F–II) remained as the fifth ranked population. Despite belonging to the same family of F–II, the nonconsensus sequence of 12–5 differs by 8 residues from 12-1 (Table 2), indicating that these mRNA templates did not emerge by a few mutations but were independently present in the initial E12 library. Two peptides, 12–2 and 12–3, belonging to F–I, also remained as the second and third populations, resulting in the overall population of F–I being the most dominant in round 7, but sharing almost the same level with the population of F–II (Figure S5D). It should be noted that there is only one residue difference of S7N in 12–2 and 12–3, whose mutation could be derived from AGT to AAT, although it is unclear whether both clones existed in the library or one emerged by mutation. It is also worth highlighting that the F–III and F–IV peptides were completely depleted by the triaged criteria. On the other hand, 12–4, which is the most unique clone in this study because of its characteristic dumbbell-type bicyclic structure, remained in the population throughout the selection campaign, presumably because it has a slow *k*_d_ (= 7.21 × 10^–3^ s^–1^) and a *k*_a_ comparable to that of 12–2.

The E14 library has given the most enlightening outcomes, maintaining major populations of both F–I and F–II peptides (Figure 3E and Table S4E), similar to the E12 results (Figure 3D and Table S4D). However, the origins of the E14 and E12 library peptides are different, as the E14 mRNA sampling space was separated from that of the E12 prior to peptide sequence generation (Figure 1B). Therefore, it was very unlikely that the E14 and E12 libraries contained identical mRNA sequences. In fact, the nonconsensus 5 residues among the F–I peptides, 12–2, 12–3, 14–2, and 14–4, are quite different, indicating that these mRNA templates were independently present in the respective E12 and E14 mRNA libraries (Table 2, S4D, and S4E). Similarly, the nonconsensus 8 residues among the F–II peptides, 12–1, 12–5, 14–3, 14–5, and 14–6, are also quite different. Thus, these F–I and F–II peptides found in the E14 library have emerged from independent mRNA templates, giving their variants with slower or comparable *k*_d_ values distinct from those in the E12 library. This means that many independent variants of F-1 and F–II peptides were successfully sampled in the E12 or higher library diversity scale (E14), yet the selection is still a convergent evolution that emerged as consensus sequences.

Second, 14–1 was the most enriched as a single clone, with the lowest *k*_d_ value among all peptides identified in the selection campaigns (Table 1 and 2, Figure 2D). Most importantly, 14–1 is a unique sequence distinct from all other families, including aMD4 and aMD5, which were previously found in a completely different set of libraries. This result clearly demonstrates the unparalleled advantage of higher diverse libraries in identifying novel peptide sequences unreachable by lower diverse libraries. Most importantly, the RaPID system should be able to discover another type of unique peptide by simply repeating the selection using a differently sampled E14 library or higher diverse libraries, *e.g*., an E16 library, if economically and practically possible.

## Discussions

The experimental details are described in the Supporting Information. Besides revealing the impact of library diversity scales on the RaPID selection, the *K*_D_s and NGS analyses highlight the crucial factors in the selection pressure: the methodology used for negative selection against naked and Fc-immobilized beads, followed by positive selection against MET-Fc-immobilized beads. Employing a ratio of target MET protein nearly equivalent to the peptide-mRNA/cDNA molecules for each round, a 1:1 binding between peptide and MET was attempted to achieve. Therefore, the *k*_a_ of >2 × 10^4^ M^–1^ s^–1^ is likely the threshold of a hit peptide that can associate with MET within the incubation time of 30 min, indicating a relatively weak selection pressure regarding *k*_a_. Conversely, after treatment of the peptide library solution with the positive beads, the durability of the peptide-target complexes was rigorously panned through three buffer washes, which was directly reflected in the dissociation rate (*k*_d_) of the respective selected peptides.

This exploration provided two profound insights with broader implications. First, the appropriate library diversity scale and selection rounds are highly dependent on the selection conditions and the purpose of the study. When lower diversity scales of libraries such as E6 and E8 were used, potent binders typically emerged in later rounds. For example, 8–3 (F–III) with a *K*_D_ of 199 nM and 8–4 (F–IV) with a *K*_D_ of 212 nM dominated the early rounds (Figure 3B, Table S4B). Stopping the selection prematurely at round 3 or 4 might have missed potent peptides such as 8–2 (F–I) with a *K*_D_ of 21.6 nM. However, the less diverse libraries completely missed potent F–II and unique peptides simply due to missing such species in the initial sampling. On the other hand, using a medium diversity scale of library such as E10, we were able to capture both weak (F–III and IV) and strong binders (F–I), but missed other potent peptides (F–II). Libraries with higher diversity scales (E12 and E14) yielded potent F–I and F–II peptides and unique peptides, but weaker binders were depleted early due to intense competition. Since these weaker species should have existed in the initial libraries, their harsh competition with potent species swept them out. These results also provide another approach to obtaining a potent binder using a lower diversity scale of library; for example, to find 14–4 from the E10 library, (1) perform several rounds of selection using the E10 library to obtain the potent binder family F–I, and (2) construct a focused library by randomizing some amino acids and performing another selection.

To assess whether the above experimental data fit with a theoretical knowledge, the so-called “extreme value theory”,^26,27^ we have simulated the correlation of the affinities of the binders with the library diversity scale. The plot visualization of *k*_d_ (or *K*_D_) versus library diversity scale (Figure S9) clearly displayed the correlation between them, where a 10^7^-fold increase in the library diversity scale gives the best binder with a 10-fold slower *k*_d_ (Figure S9B). Also, the plot shown in Figure S9C reproduced the *lognormally distributed affinities model* reported by Tanaka et al.^27^ However, it should be noted that the absolute values of *k*_d_ (or *K*_D_) described in this figure could vary if the library design and the unnatural amino acid usage are changed.^28,29^*(Although we could see some trend between the K*_*D*_*of the hit peptide and the diversity scale of the library as shown in*Figure 2 and S9, *the statistical analysis did not show the significance using the current data set. Further analysis including the data of additional synthesized peptides is shown in*Figure S10 and Table S5*.)*

Second, as discussed above, the selection pressure applied in our experiments was based on the slow *k*_d_ of the peptides. However, we also observed that some peptides, such as 6–1 and 8–1, that have faster *k*_a_ compete out with species that have a similar *k*_d_ property. Since the washing procedure of the peptide-bound beads is the critical step in selecting peptides with slower *k*_d_, changing the procedure and conditions could be practical to alter the peptide populations, resulting in the discovery of species with desired *k*_a_ and *k*_d_ properties.

The possible procedures for adjusting the selection pressure are as follows: (i) Short incubation time and lower target concentration would select peptides with faster *k*_a_ through the competitive binding. (ii) In a highly diverse library (>10^12^), since only one copy or a few copies of each sequence exists in the first round, to reduce the risk of losing potential binders (bearing even faster *k*_d_), the conditions for washing the peptide-bound target on beads can be controlled, *e.g*., one wash vs three washes; however, such conditions may leave nonspecific binders behind, so that careful NGS data analysis is required to distinguish specific binders from nonspecific ones. (iii) If the target does not have a potential binding site for peptides to acquire slow *k*_d_, milder washing conditions can be applied for the recovery of moderate binders. (iv) Adding the free (unimmobilized) target protein to the selection mixture may capture the species with fast *k*_d_, yielding peptides with “extreme” slow *k*_d_ values.^30,31^

## Conclusions

In this study, we performed the five RaPID selection campaigns in parallel against the ectodomain of MET using the peptide libraries expressed from 10^6^, 10^8^, 10^10^, 10^12^, and 10^14^ unique members of the mRNAs (E6–E14). We thoroughly analyzed the outcomes, including the binding kinetic properties, of the peptide families enriched by the respective selections, identifying four families (Fs) and four independent species. Among them, F–I represents the most potent family of macrocycles with single-digit nM *K*_D_ values and slow *k*_d_ found from the E12 and E14 libraries, while a unique macrocycle, 14–1, present only in the E14 library, also exhibited the affinity in the same range. NGS data of the enriched libraries revealed that the initial sampling of the sequence space determined the evolution of the families through selective pressure for slow *k*_d_ values. Interestingly, the weaker binders F–III and F–IV, which should exist in all initial libraries of E6 and E14, were mainly found in the less diverse libraries (E6 and E10), whereas they were completely depleted in E12 and E14 due to the competition with potent peptide families of F–I and F–II with slow *k*_d_.

Although we conducted each selection only once, it is impossible to reconduct the same selection using the exact same library due to unavoidable contaminations from the previous selection. However, it should be noted that this experiment is a repeat of our previously reported work,^22^ and the same family of peptide including the critical motif for binding to MET was found in the present work, *i.e*., the experimental reproduction was clearly made. Moreover, we found new families and independent rare species. This means that the main conclusion of the “diversity scale of library matters” reported herein can be generalized at least for the RaPID selection platform and possibly for other platforms. Most importantly, this study provides valuable guidelines for the design of libraries with different diversity scales and selection conditions to enrich macrocyclic peptides with desired characteristics using not only the RaPID system.

## Methods

No unexpected or unusually high safety hazards were encountered during this research.

All experimental methods can be found in the Supporting Information.

## Data Availability

The next-generation sequencing data are available via NCBI Bioproject via accession ID PRJNA1151868.

## Supporting Information Available

Materials; methods; representative peptides identified from the combinational libraries; primer list; deep-sequencing results; enrichment tracing result; library quality check (Urea-PAGE and deep-sequencing result); the RaPID selection scheme; recovery rate; structure determination for 10–4 and 12–4, the population of each peptide family; UPLC; MALDI-TOF MS; SPR sensorgrams; correlation fitting chart between *K*_D_s or *k*_d_s of hit peptides and the library diversity scale, and references of representative identified peptides (PDF)
NGS result of five libraries at each selection round and the processed data of recovered peptides’ read number (ZIP)
Transparent Peer Review report available (PDF)

## Abbreviations

*K*_D_: dissociation constant
*k*_a_: association rate
*k*_d_: dissociation rate
mRNA: messenger ribonucleic acid
PCR: polymerase chain reaction
SPR: surface plasmon resonance
