# Supplementary material for: Diversity Scale of Library Matters: Impact of mRNA Library Diversity Scales on the Discovery of Macrocyclic Peptides Targeting a Protein by the RaPID System
Source: ACS Cent Sci. 2025 Mar 10;11(3):431–40. doi: 10.1021/acscentsci.4c01021 (PMC11950852; doi:10.1021/acscentsci.4c01021)
Supplement: Supplementary file 1 — oc4c01021_si_001.pdf [file oc4c01021_si_001.pdf]

Supporting Information

Diversity scale of library matters: Impact of mRNA library diversity scales on the discovery of macrocyclic peptides targeting a protein by the RaPID system

Jinxuan Zhao[1], Yi Li[1], Naohiro Terasaka[2], Haruo Aikawa[1], and Hiroaki Suga[1*]

[1]Department of Chemistry, Graduate School of Science, The University of Tokyo, Bunkyo, Tokyo 113-0033, Japan

[2]Earth-Life Science Institute, Tokyo Institute of Technology, Meguro, Tokyo 152-8550, Japan

[*]Corresponding author, E-mail: hsuga@chem.s.u-tokyo.ac.jp

This supporting information references 188 other publications.

Supporting Information includes: Materials; representative peptides identified from the combinational libraries; primer list; statical comparison of median values of K_D among E6-14 libraries; deep-sequencing results; enrichment tracing result; library quality check (Urea-PAGE and deep-sequencing result); the RaPID selection scheme; recovery rate; structure determination for 10-4 and 12-4, the population of each peptide family; UPLC; MALDI-TOF MS; SPR sensorgrams; correlation fitting chart between K_Ds or k_ds of hit peptides and the diversity scale of library, and references of representative identified peptides.

Note: At the request of the editor, we synthesized 4 peptides from the E6 library and 1 peptide from the E8 library (Table S3) and performed SPR measurement (Figure S10). The results of the statistical analysis are shown in Table S5.

General Procedures

Materials

Reagent	Source	Identifier
**Oligonucleotides**		
PCR primers used for the library construction or the RaPID system selection, see **Table S2**	Eurofins Genomics, Tokyo, Japan	N/A
Puromycin construct: 5’-pCTCCCGCCCCCCGTCC–PEG linker–CC–puromycin-3’	Gene Design Inc, Osaka, Japan	N/A
**Recombinant Proteins and enzymes**		
His-/ hFc-tagged human MET ectodomain	Sino Biological Inc.	10692-H03H
Recombinant human IgG1 Fc	R&D systems	110-HG-100
M-MLV reverse transcriptase (H-)	Promega	M3682
RNasin RNase inhibitor	Promega	N2615
RQ1 DNase	Promega	M6101
Enzymes without further specification	Expressed by Suga Group members	N/A
**Critical Commercial Assays**		
Series S Sensor Chip Protein G	Cytiva	29179315
MiSeq Reagent Kit v3 (150-cycle)	illumina	MS-102-3001
TapeStation D1000 ScreenTape/ TapeStation D1000 Reagent Kit	Agilent	5067-5582/ 5067-5583
**Chemicals**		
Chemicals for premixed buffers and other procedures	Nacalai Tesque, Tokyo Chemical Industry, Sigma-Aldrich Japan, or Fujifilm Wako Chemicals	N/A
Amino acid derivatives and coupling reagents for SPPS	Watanabe Chemical Co., Ltd	N/A
**Software and Online Tools**		
Biacore™ Insight Software	Cytiva	29310602
WEBLOGO 2.8.2	https://weblogo.berkeley.edu/	N/A

Methods

mRNA Library design

Translation initiator (27 bp)	(Start) (3 bp)	NNK 15 (45 bp)	Cys (3 bp)	Linker (18 bp)	(Stop) (3 bp)	Puromycin linker overlap (15 bp)
GUUGAACUUUAAGUAGGAGAUAUAUCC	AUG	$(NNK)_{15}$	UGU	UCCGGCGGAUUAACUAAC	UAG	GACGGGGGGCGGAAA

Phenol/Chloroform/Isoamyl alcohol (PCI) extraction

An equal amount of phenol/chloroform/isoamyl alcohol (25:24:1) was added to the solution, mixed vigorously, and centrifuged at 13,000 rpm for 5 minutes. The supernatant was added to an equal amount of chloroform/isoamyl alcohol (24:1) and mixed vigorously again. After 2 minutes of 13,000 rpm centrifugation, the supernatant was recovered.

Ethanol precipitation

If necessary, a 10% volume of 3 M NaCl was added to make samples containing 0.3 M NaCl. Twice the amount of 95% ethanol was added, mixed vigorously, and centrifuged at 13,000 rpm for 15 min. After removing the supernatant, 70% ethanol was added gently to wash the pellet and centrifuged for 3 min. After removing the supernatant, the resulting pellet was dried and dissolved with water by 10% of the initial solution.

Isopropanol precipitation

To the sample, 80% volume of isopropanol was added, mixed vigorously, and centrifuged. After removing the supernatant, 70% ethanol was added gently to wash the pellet and centrifuged at 13,000 rpm for 3 min. After removing the supernatant again, the resulting pellet was dried and then dissolved with water.

mRNA purification using urea denaturing polyacrylamide gel electrophoresis (PAGE)

The crude mRNA solution was mixed with the same volume of 2×RNA loading buffer (8 M Urea, 2mM $Na_2EDTA \cdot 2H_2O$, 2mM Tris, 2% BPB) and denatured by incubating at 95 °C for 1 min. The sample was then loaded to the polyacrylamide gel (8% Acrylamide: Bis = 19:1, 6 M Urea), and 250-volt electrophoresis for 70 min was conducted. The mRNA was harvested by soaking the gel piece that constrained the mRNA band with 0.3 M NaCl, followed by the Ethanol precipitation.

Agarose gel electrophoresis

A mixture of 2 μL of PCR product and 2 μL of 2x DNA loading buffer were loaded to the wells of 3% agarose gel. After electrophoresis was performed at 135 V for 10 min, DNA bands were analyzed by UV light using Printgraph 2M (ATTA).

TapeStation D1000 electrophoresis

The length of the PCR product was analyzed using the TapeStation D1000 electrophoresis (Agilent) following the instructions.

Extension reaction

Reverse primer NNK15.ex.R91 was used for extension in a 700 µL scale. A mixture of 7.0 µL of 100 µM T7.F53 and 7.0 µL of 100 µM reverse primer were prepared and carried out for extension PCR in the KOD mixture containing 120 mM Tris-HCl (pH 8.0), 6 mM $(NH_4)_2SO_4$, 2.5 mM $MgCl_2$, 10 mM KCl, 0.001% AcBSA (v/v), 0.1% Triton X-100 (v/v), 250 µM each dNTPs and 1.5% (v/v) 1×KOD DNA polymerase. Then extension (94 °C, 1 min→ (55.9 °C, 1 min→ 68 °C, 1 min) ×2 cycles→ 68 °C, 5 min) was carried out. 4.21×10^{14} different sequences were expected to generate using LifeECO ver3.0 (Bioer Technology).

10^{14} diversity scale (E14) library construction

A large part of the extension reaction product (650 µL, containing 3.91×10^{14} different sequences) was aliquoted and amplified with PCR in a 6,500 µL scale using the same KOD mixture (94 °C, 1 min→ (94°C 40 sec→ 62.9 °C, 40 sec →68 °C, 40 sec) ×2 cycles). PCR amplification was replicated until the DNA band was checked on Agarose gel. After checking the DNA band with TapeSation (Agilent), PCI extraction and ethanol precipitation were conducted. The pellet was dissolved into 650 µL of 50 mM KCl solution. All the solution was used for *in vitro* transcription at 37 °C for 15 hrs in a 6,500 µL scale T7 mixture containing 40 mM Tris-HCl buffer (pH 8.0), 22.5 mM $MgCl_2$, 10 mM dithiothreitol, 1 mM spermidine, 0.01% Triton X-100, 3.75 mM NTP mix, 0.04 U μL^{-1} RNasin RNase inhibitor (Promega, N2615) and 120 nM T7 RNA polymerase. DNA templates were digested by adding RQ1 DNase (Promega, M6101) at 37 °C for 1 hr. Then, PCI extraction and isopropanol precipitation was conducted. The resulting RNA pellet was dissolved in water. The mRNA was purified by an 8% denaturing polyacrylamide gel electrophoresis (PAGE) containing 6 M urea. The purified mRNA pellet was dissolved in water. The concentration of the mRNA was measured by NanoDrop (Thermo Fisher) and adjusted to 10 µM by adding distilled water.

10^{12} diversity scale (E12) library construction

A small part of the extension reaction product (6.5 µL, containing 3.91×10^{12} different sequences) was aliquoted and amplified with PCR in 65 µL scale using the KOD mixture (94 °C 1 min→ (94 °C, 40 sec→ 62.9 °C, 40 sec →68 °C, 40 sec) ×2 cycles). PCR amplification was replicated until the DNA band was checked on Agarose gel. PCI extraction and ethanol precipitation were conducted after checking the DNA band with TapeSation (Agilent). The pellet was dissolved into 6.5 µL of 50 mM KCl solution, and all the DNA solution was used for *in vitro* transcription in 65 µL scale T7 mixture, followed by the treatment of RQ1 DNase at 37 °C for 1 hr. Then, PCI extraction and isopropanol precipitation were conducted. The resulting RNA pellet was dissolved in water. The mRNA was purified by an 8% denaturing polyacrylamide gel electrophoresis (PAGE) containing 6 M urea. The purified mRNA pellet was dissolved in water. The concentration of the mRNA was measured by NanoDrop (Thermo Fisher) and adjusted to 10 µM by adding water.

10^{10} diversity scale (E10) library construction

An aliquot (0.37 µL) of 10 µM E14 mRNA library solution was diluted 10^4 times with distilled water. A 37

μL of the diluted solution (2.23×10^{10} different sequences were expected) was used for reverse transcription (RT) in a 57.8 μL scale. The RT mixture contained 0.73 mM dATP, 0.73 mM dGTP, 0.73 mM dCTP, 0.73 mM dTTP, 5 μM LTN.R36 primer, 73 mM Tris-HCl (pH 8.3), 43.8 mM $Mg(OAc)_2$, 29.2 mM KOH and 4.5% (v/v) M-MLV reverse transcriptase lacking RNase H activity (Promega, M3682). All RT product was used for PCR in a 578 μL scale using the KOD mixture (94 °C, 1 min→ (94 °C, 40 sec→ 62.9 °C, 40 sec →68 °C, 40 sec) ×2 cycles). PCR amplification was replicated until the DNA band was checked on Agarose gel. PCI extraction and ethanol precipitation were conducted after checking the DNA band with TapeSation (Agilent). The pellet was dissolved into 57.8 μL of 50 mM KCl solution, and all the DNA solution was used for *in vitro* transcription in a 578 μL scale with T7 mixture, followed by the treatment of RQ1 DNase at 37 °C for 1 hr. Then, PCI extraction and isopropanol precipitation was conducted. The resulting RNA pellet was dissolved in water. The mRNA was purified by an 8% denaturing polyacrylamide gel electrophoresis (PAGE) containing 6 M urea. The purified mRNA pellet was dissolved in water. The concentration of the mRNA was measured by NanoDrop (Thermo Fisher) and adjusted to 10 μM by adding distilled water.

10^8 diversity scale (E8) library construction

An aliquot (4.23 μL) of 10 μM E12 mRNA library solution was aliquoted and diluted 10^4 times with distilled water. A 42.3 μL of the diluted solution (2.54×10^8 different sequences were expected) was used for reverse transcription (RT) in a 66.1 μL scale using the RT mixture. All RT product was used for PCR in a 661 μL scale using the KOD mixture (94 °C, 1 min→ (94 °C, 40 sec→ 62.9 °C, 40 sec →68 °C, 40 sec) ×2 cycles). PCR amplification was replicated until the DNA band was checked on Agarose gel. PCI extraction and ethanol precipitation were conducted after checking the DNA band with TapeSation (Agilent). The pellet was dissolved into 61 μL of 50 mM KCl solution, and all the DNA solution was used for *in vitro* transcription in a 611 μL scale with the T7 mixture, followed by the treatment of RQ1 DNase at 37 °C for 1 hr. Then, PCI extraction and isopropanol precipitation was conducted. The resulting RNA pellet was dissolved in water. The mRNA was purified by an 8% denaturing polyacrylamide gel electrophoresis (PAGE) containing 6 M urea. The purified mRNA pellet was dissolved in water. The concentration of the mRNA was measured by NanoDrop (Thermo Fisher) and adjusted to 10 μM by adding distilled water.

10^6 diversity scale (E6) library construction

An aliquot (4.77 μL) of 10 μM E10 mRNA library solution was aliquoted and diluted 10^4 times with distilled water. A 47.7 μL of the diluted solution (2.87×10^6 different sequences were expected) was used for reverse transcription (RT) in a 74.5 μL scale. All RT product was used for PCR in a 745 μL scale with the KOD mixture (94 °C 1 min→ (94 °C, 40 sec→ 62.9 °C, 40 sec →68 °C, 40 sec) ×2 cycles). PCR amplification was replicated until the DNA band was checked on Agarose gel. PCI extraction and ethanol precipitation were conducted after checking the DNA band with TapeSation (Agilent). The pellet was dissolved into 74.5 μL of 50 mM KCl solution, and all the DNA solution was used for *in vitro* transcription in a 745 μL scale with T7 mixture, followed by the treatment of RQ1 DNase at 37 °C for 1 hr. Then, PCI extraction and isopropanol precipitation was conducted. The resulting RNA pellet was dissolved in water. The mRNA was purified by 8% denaturing polyacrylamide gel electrophoresis (PAGE) containing 6 M

urea. The purified mRNA pellet was dissolved in water. The concentration of the mRNA was measured by NanoDrop (Thermo Fisher) and adjusted to 10 μM by adding distilled water.

Quality validation of mRNA libraries

The length of each mRNA library was analyzed on the same polyacrylamide gel (8% Acrylamide: Bis = 19:1, 6 M Urea). After electrophoresis, the gel was dyed with EtBr (0.5 µg/ml) and then analyzed with Typhoon FLA 7000 (532 nm laser, O580 filter). The mRNA libraries were then amplified following the sample preparation procedure of the Miseq v3 kit (150 cycles) (Illumina) and analyzed by the next-generation sequencing using the same kit. The codon bias was then visualized using WEBLOGO 2.8.2 (Figure S1).

***In vitro* selection of macrocyclic peptides binding to MET**

The previous study utilized two libraries initiated by *N*-chloroacetyl-$^{L/D}$Tyr (*N*-ClAc-$^{L/D}$Tyr) followed by random sequences $(NNK)_n$ (n = 4–15, where N and K represent any of the four bases, and U or G, respectively) and a Cys, giving a macrocyclic structure via spontaneous thioether formation between the *N*-ClAc group and thiol group of the Cys. With $(NNK)_{4–15}$ codons in the designed library, the diversity of mRNA sequences is calculated as $\sum_{n=4\text{-}15} (32)^n = 3.9 \times 10^{22}$. In the current protocol, 150 µL of translation solution is the maximum scale generally, which contains 10^{14} molecules of ribosomes (*vide infra*). Thus, based on the theoretical diversity of the mRNA library (3.9×10^{22}), even at 150 µL scale of the translation (containing 1.1×10^{14} molecules of ribosome), the coverage of the mRNA sequences would be 2.8×10^{-7} %. The preparation of ClAc-^{L}Tyr precharged tRNA was carried out following the previously described protocol[19]. The RaPID system selection was carried out following the previously described protocol[40].

Clone assay for cyclization position determination

To uniquely determine the cyclization position in peptides 10-4 and 12-4, DNA templates of 10-4 (C(14,16)S; C(14, 17)S and C(16, 17)S), 12-3 (C(3, 8)S; C(3, 17)S and C(8, 17)S) were prepared by PCR amplification using KOD mixture, followed by *in vitro* transcription, mRNA purification, and Puromycin ligation into 6 μM Pu-mRNA, in the same procedure described above. The macrocyclic peptide 10-4 and 12-3 mutants with only one cysteine cyclization position can be obtained by the translation of the mRNAs. The peptide was subjected to the MET-immobilized beads in the same procedure with RaPID selection. The cyclization position is determined by the recovery rate of each peptide (Figure. S4).

Syntheses of peptides by SPPS (excepting peptide 10-4 and 12-4)

The SPPS was carried out following the previously described protocol[40]. The on-resin coupling of peptide residues was carried out using Syro II (Biotage). The purification and purity analysis was conducted with reversed-phase HPLC on a Prominence LC 20AP system (Shimadzu) with a Chromolith Prep column (100mm by 25-mm internal diameter; MerckMillipore), MALDI-TOF (Ultra Flex; Bruker Daltonics) and reversed-phase ultra-HPLC(UPLC) analysis with a Nexera X2 system (Shimadzu) fitted with a C_{18} reversed-phase column (Waters) (Figure S6 and S7).

Synthesis of peptide 10-4 and 12-4 by SPPS

Peptide synthesis was conducted in the same protocol as other macrocyclic peptides except for the following procedures. For the cysteine used for cyclization with the ClAc group at the N-terminus, Cys(Trt) was used, and Cys(Thio-tBu) was used for the other Cys. After SPPS, the peptides on the resin were incubated with 2 mL of 95% (v/v) TFA and 5% (v/v) *m*-cresol at room temperature for 3 h with rotation to deprotect and cleave off from the resin. After the macrocyclization, 500 μL of H_2O and 250 μL of tributylphosphine were added to the peptide solution, and the mixture was vigorously shaken for 2 hrs to deprotect Cys(Thio-tBu). Peptides were validated samely. For lariat peptide 12-4, the formation of a disulfide bond between Cys 8 and Cys 17 was observed from MALDI-TOF-MS.

Binding kinetics analysis of peptides by SPR

The binding affinities of selected peptides targeting Fc-fused MET were analyzed by SPR using a Biacore 8K instrument (Cytiva) at 25 °C. The composition of the running buffer was HBS EP+ buffer (10 mM HEPES pH 7.4, 150 mM NaCl, 3 mM EDTA, and 0.05% (v/v) Surfactant P20) containing 0.1% DMSO. The target protein, Fc-fused MET, was immobilized on a Biacore sensor chip ProteinG (Cytiva) to 1,500–2,000 response units. The kinetic constants were determined by a single-cycle kinetics analysis by injecting five different concentrations (two-fold dilution series) of each peptide at the flow rate of 30 μL min^{-1}. Binding sensorgrams were fitted using the standard 1:1 interaction model and analyzed using Biacore™ Insight Software (Figure S8).

Table S1A. Representative peptide sequences discovered by RaPID system. Residues engaged in thioether-macrocyclization are marked with asterisks.

mRNA display (RaPID system)				Diversity scale: >10^{12}		
Screening Target	K_D (nM)	IC_{50} (nM)	Library design/ Note	Consensus motif/ Peptide sequence	Variants	Reference
E6AP	0.6		^{D}W*X$_{8-15}$C*	^{D}W*X$_{2}$^{Me}SX$_{2}$^{Me}F-^{Me}G/X-X^{Me}FPC*	6	(32)
Akt2		92	$^{L/D}$Y*X$_{4-12}$C*	^{L}Y*WIX$_{2}$TWPXVXRXC*	2	(33)
		110		^{L}Y*ILVRNRXLRVDC*	2	
SIRT2	3.8		$^{L/D}$Y*-X$_{n}$-LysTfa-X$_{n}$C*	^{L}Y*SNFRI-LysTfa-RYSNSSC*	21	(34)
	3.7			^{D}Y*HDYRI-LysTfa-RYHTYPC*	16	
VEGFR2	94		$^{L/D}$F*X$_{8-15}$C*	^{L}F*VVVSTDPWVNGLYIDC*	2	(35)
	8			^{L}F*IGHYRVKVHPISLERC*	1	
	2			^{D}F*KPDWWTYYDLRHPC*	3	
	33			^{L}F*WELK^{D}YLNTR^{D}YC*	1	
cMET	19		$^{L/D}$Y*X$_{4-15}$C*	^{L}Y*ISWNEFNSPNWRFITC*	1	(16)
	2.3			^{D}Y*WYYAWDQTYKAFPC*	5	
	2.4			^{D}Y*RQFNRRTHEVWNLDC*	1	
EpCAM	1.7		^{D}W*X$_{4-12}$C*	^{D}W*RPTRYRLLPWWIC*	1	(36)
	1.6			^{D}W*SRIALRLGLIC*	7	
PlexinB1	3.5		^{D}W*X$_{10-15}$C*	^{D}W*RPRVARWTGQIIYC*	8	(37)
	274.7			^{D}W*NSNVLSWQTYSWYC*	2	
	700.5			^{D}W*AVSNLPPRPHITYIEC*	1	
HPA		K_i=1±0.1	$^{L/D}$Y*X$_{6-15}$C*	^{D}Y*PYSC*WARHVRIREN	3	(38)
iPGM		5.3	$^{L/D}$Y*X$_{4-12}$C*	^{D}Y*SWPNAPEIWKC*C	1	(39)
		50.5		^{D}Y*QNRSIVVLYGC*C	1	
		5.13		^{D}Y*AVWPNC*RTC	4	
		8.4		^{D}Y*DYPGDHC*YLYGTC	2	
		5.22		^{L}Y*TTLANPFRILHC*	2	
KDM4A-c	29.8		$^{L/D}$Y*X$_{4-12}$C*	^{D}Y*VYNTRSGWRWYTC*	1	(40)
	36			^{L}Y*KSRRSGLTWIPC*	1	
	173			^{L}Y*SKYTQSGVRWVC*	1	
eVP24	3.1	28	$^{L/D}$Y*X$_{7-15}$C*	^{D}Y*IVPWNGGSRLIRNSRC*	2	(41)
	3.2	9		^{L}Y*TRWQGGLRYIRPC*	1	
	963			^{L}Y*YSSRWNHGHFTPC*	7	
	3.6	37		^{L}Y*FKSVRTGLRYVYC*	1	
TET1CCD	22.7		^{D}Y*X$_{4-15}$C*	^{D}Y*LIYAYYTWWEHSC	1	(42)

Table S1A. (Continued)

Screening Target	K_D (nM)	IC_{50} (nM)	Library design/ Note	Consensus motif/ Peptide sequence	Variants	Reference
TET1CCD	48.6			^{D}Y*WYLPSYRVPWFC	1	
tPHD2	5.51		^{D}Y*X$_{4-12}$C*	^{D}Y*IWDTGTFYLSRTC*	2	(43)
	0.27			^{D}Y*VWLTDTWVLSRTC*	1	
	0.17			^{D}Y*VWDPRTFYLSRIC*	1	
	1.46			^{D}Y*WNPLTIYVSRVC*	1	
	0.75			^{D}Y*GWPPIVSRTWPYC*	1	
IL6R	44		^{L}Y*X$_{8-9}$C* Hse: (S)-2-Amino-4-hydroxybutyric acid	^{D}Y*TT-GluMe-^{Me}TyrMe-^{Me}Gly-Ala2Thi-I-Ala2Thi-YC*	18	(44)
	357			^{D}Y*YL-GluMe-^{Me}TyrMe-^{Me}Gly-Ala2Thi-HseMe-Ala2Thi-GluMe-C*	22	
	446			^{D}Y*LAWLFETQTWRIC*	2	
	52			^{D}Y*LWSIRNPVDNSHYWPC*	2	
	912			^{D}Y*SWDPFSVWEATGWIC*	2	
NTCP	1,340		$^{L/D}$Y*X$_{4-15}$C*	^{D}Y*ALWIWPQVYWTRFSC*	1	(45)
	14			^{D}Y*WWAINPHIHVFTWPC*	1	
	50			^{D}Y*HYWYPEWNTALYIWPC*	1	
	2,270			^{D}Y*ILVYPELHLFLLYSC*	1	
	21			^{L}Y*LYTWTWHSNWTVTSLC*	1	
	374			^{L}Y*VWLLWAERSWFLDLGC*	1	
	13			^{L}Y*LWIFHQNWYTFDLGC*	1	
	30			^{L}Y*LYLWTWPNTSYAYNVC*	1	
	4			^{L}Y*TWNTHHYYIWLNC*	1	
	36			^{L}Y*SYIDTFTWIWLYC*	1	
Zika Virus NS2B-NS3	20		$^{L/D}$Y*-X$_{8-10}$C*	^{L}Y*WKI-^{Me}YMe-NTLVNIC*	87	(46)
	8.7			^{L}Y*-^{Me}YMe-K^{Me}FK^{Me}S-^{Me}YMe-K-^{Me}YMe-^{Me}YMe-KC*	4	
	7.5			^{L}Y*TNFYLYPY-^{Me}YMe-FC*	3	
	8.9			^{L}Y*^{Me}GIAKYN-^{Me}YMe-^{Me}YMe-IPC*	2	
	5			^{L}Y*TLPFHN^{Me}GTFFC*	3	
	168			^{D}Y*AII-^{Me}YMe-YNKY^{Me}LNC*	100	
HGF	0.4		^{D}W*X$_{4-15}$C*	^{D}W*PLSKWWYSKRC*	1	(47)
	1.3			^{D}W*DYITKWWKC*	2	
	12			^{D}W*KFRISKWYKC*	3	
	2.1			^{D}W*IFKISKWYKC*	2	
K48Ub2	14		^{D}W*X$_{6-12}$C*	^{D}W*GWFDNLYWYVTHC*	10	(48)

Table S1A. (Continued)

Screening Target	K_D (nM)	IC_{50} (nM)	Library design/ Note	Consensus motif/ Peptide sequence	variants	Reference
	14			^{D}W*WYDREYYYLGYGC*	1	
	26			^{D}W*GWFDDLYFVAYC*	9	
	6			^{D}W*LYLDDSGDWWIC*	1	
EGFR	16		^{L}Cba*X_{6-15}C* ^{L}Cba: L-Carboranylalanine	^{L}Cba*REWQLQRYGTHWPC*	1	(49)
	20			^{L}Cba*VNEKVAEVSAGHYSC*	1	
	113			^{L}Cba*DVEHPIRYWADLC*	1	
K48Ub2	141		$^{L/D}$Trp/pClBz/mClBz*X_{10-15}C* mClBz: 3-(chloromethyl)benzoic acid pClBz: 4-(chloromethyl)benzoic acid	pClBz/mClBz*WFDREYYYLGYGC*		(50)
	16			mClBz*GWFDNLYWYITHC*		
	10			mClBz*GWFDINYLFVAYC*		
Wnt3a	490		$^{L/D}$Y*X_{6-15}C*	^{D}Y*NIWVFNLPTLTLYRC*		(51)
	171			^{D}Y*RKYLYERFWWC*		
	318			^{D}Y*LYRVPNRRVFVLVLC*		
	110			^{L}Y*LVYDHLYAWWYFIAC*		
	39.5			^{D}Y*RKYLYERFPWC*		
CCL11	13.7	1000	$^{L/D}$Y*X_{4-15}C* sTyr: sulfotyrosine	^{L}Y*LAPWVWDA-sTyr-IET-sTyr-C*	1	(52)
	25.2	900		^{L}Y*DPQTC*-sTyr-LW-sTyr-SPIIYS	1	
	10.4	160		^{D}Y*-sTyr-sTyr-C*VDWGL-sTyr-RPIET-sTyr-S	3	
	26.6			^{D}Y*-sTyr-C*-sTyr-sTyr-APVILL-sTyr-AD-sTyr-S	8	
SIRPa-B6/ NOD	12.3		$^{L/D}$Y*X_{6-15}C*	^{D}Y*KDWSISARITPVHPC*	4	(53)
	8.22			^{D}Y*RYSAVYSIHPSWC*	10	
	5.97			^{L}Y*YALSDRYTALYPKPC*	14	
hFXIIa	8.21		^{D}Y*X_{6-15}^{D}C* 1: (1S. 2S)-2-ACHC; 2: (1R. 2R)-2-ACPC ; 3: (1S. 2S)-2-ACPC	^{D}Y*NDRSTR2RLVA ^{D}C*	11	(54)
	0.58			^{D}Y*PRLFN2SYLRR ^{D}C*	15	
	0.98			^{D}Y*FAYDRR1LSNN1RNY ^{D}C*	4	
	155			^{D}Y*RYT1NRLF1NA ^{D}C*	2	
FXIIa	5		^{D}Y*X_{6-15} ^{D}C* 1: (1S. 2S)-2-ACHC; 2: (1R. 2R)-2-ACPC ; 3: (1S. 2S)-2-ACPC	^{D}Y*FGVR2FYNRT ^{D}C*	15	(55)
	5.76			^{D}Y*FSLR2SFNRSRG ^{D}C*	3	
	1.87			^{D}Y*FGV32FYNRT ^{D}C*	16	
	12.3			^{D}Y*PRYNL3GASPRF2N ^{D}C*	1	

Table S1A. (Continued)

Screening Target	K_D (nM)	IC_{50} (nM)	Library design/ Note	Consensus motif/ Peptide sequence	variants	Reference
GppNHp	9		^{D}Y*X$_{8-12}$C*	^{D}Y*FVNFRNFRTFRC	3	(56)
AgrC		4.2	$^{L/D}$Y*X$_{4-15}$C*	^{D}Y*VRTLFGWLC	2	(57)
		1.8		^{D}Y*SLFGCFL	48	
		1.1		^{L}Y*RHLFGVLPC	50	
		106.5		^{L}Y*LIKHFLGGIKVAC	24	
BRD3-BD1	260		W*X$_{3-7}$^{Ac}KX$_{4-7}$C* ^{Ac}K: acetyl-Lysine	W*TYLRWT^{Ac}KLTRIWISC*	16	(58)
	0.49			W*KTI^{Ac}KG^{Ac}KTWRT^{Ac}KQC*	19	
	0.08			W*WIIP^{Ac}KVK^{Ac}KGC*	140	
				W*^{Ac}KKAILPG^{Ac}KILKTLHTC*	2	
	13			W*SWLC*K^{Ac}KYNLIH	40	
	25			W*^{Ac}KQWK^{Ac}KYGLKIC*	265	
	27			W*^{Ac}KNWC*WL^{Ac}KR^{Ac}KLLLR	140	
	1.1			W*AYHTIRL^{Ac}KWR^{Ac}KPSISC*	50	
	36			W*^{Ac}KGYLC*LR^{Ac}KRIQRTYN	25	
K48Ub4	9		^{D}F*X$_{6-12}$C* Aoc: 8-Aminooctanoic acid	^{D}F*QYW-Aoc-Y^{Me}AlaT^{Me}GlyVC*	14	(59)
	18			^{D}F*QYW-Aoc-H^{Me}AlaI^{Me}AlaLC*	14	
hEGFR	523		^{D}W*X$_{4-15}$C*	^{D}W*RPFPG^{D}S^{D}A^{D}SF^{D}HDYPC*	ND	(60)
	777			^{D}W*RYN^{D}HYRF^{D}APYVFVC*C	ND	
	998			^{D}W*^{D}H^{D}YVYLN^{D}A^{D}ARFL^{D}YYC*	ND	
iHA	1.5		$^{L/D}$W*X$_{4-15}$C*	^{L}W*TGDFFSSHYTVPRC*	1	(61)
FXIIa		62	^{L}Y*X$_{7-15}$C*	^{L}Y*LRFRPKDRGGC*	8	(62)
		234		^{L}Y*LRFKHISLGGC*	6	
		97		^{L}Y*LRFTLC*RRAPNS	9	
		ND		^{L}Y*LIC*VSRRAFFTDQS	5	
		331		^{L}Y*LRLKPTNQGGC*	2	
		372		^{L}Y*LRFTLC*RFESALS	2	
ATP-binding cassette (ABC) transporters	20		^{D}Y*X$_{10-15}$C*	^{D}Y*SHCLENFWLTILC*	34	(63)
	20			^{D}Y*TPDFSGFLAFLRNAC*	52	
	20			^{D}Y*DPSANWILVLLTC*	4	
	50			^{D}Y*FYVWNPFVFKC*	5	
CIB1	6	12	^{L}Y*X$_{6-11}$C*	^{L}Y*KQPYWLINWC*	16	(64)
		15		^{L}Y*TTPTWFIRFC*	18	
		65		^{L}Y*QQSTWRINWC*	8	
		12		^{L}Y*TGPWTIRWC*	15	

Table S1A. (Continued)

Screening Target	K_D (nM)	IC_{50} (nM)	Library design/ Note	Consensus motif/ Peptide sequence	variants	Reference
FXIIa	0.9	K_i=0.37	XCX$_6$CRRDSDCPGA CICX$_5$C	I-PRIGRL-RATRF	1	(65)
	10	12		I-PRIVY-IRRTY	6	
	23	75		V-PRLLRW-ARGGL	1	
	14	5.5		V-PRVGWR-YPTKW	5	
	82	207		R-CGGYLV-VFKKH	6	
IFNGR1	0.44	9.7	^{D}Y*X$_{4-7}$-Abz/Atp-X$_{4-6}$^{D}C*; X: (1S,2S)-2-ACPC; Z: (1R,2S)-2-ACPC; B: Abz, 2-Aminobenzoic acid J: Atp, 3-aminothiophene-2-carboxylic acid	^{D}Y*VRRIALBNPRVV^{D}C*	ND	(55)
	2.5	400		^{D}Y*VRRLNBNNVNRL^{D}C*	ND	
	62	12		^{D}Y*VSZNRBSHINRH^{D}C*	ND	
	2.6	300		^{D}Y*FSLXBRRIHH^{D}C*	ND	
				^{D}Y*ISIRXRYJVAITST ^{D}C*	ND	
	29	56		^{D}Y*VSZYRRJTYHN^{D}C*	ND	
	35	130		^{D}Y*IAIRRJNPHHST^{D}C*	ND	
	58	190		^{D}Y*IILRRJNVIHSR ^{D}C*	ND	
human SAGA deubiquitinating module		K_i=30.8	$^{L/D}$Y*X$_{6-15}$C*	^{L}Y*TLNFRFWSDHISWYSC*	ND	(66)
		20.6		^{D}Y*AISYRGKTTLLC*	ND	
		27.7		^{D}Y*HQWSYRGATYRSC*	ND	
		63.7		^{D}Y*NGPSYYRLRSWLSSC*	ND	
		805		^{D}Y*WITHSYRGLSKSFC*	ND	
		170		^{D}Y*QTWSWRGRRYSFC*	ND	
				^{L}Y*LHDSAFSFGWSLRLYC*	ND	
				^{D}Y*RQWSFRGRTFWSC*	ND	
hEGFR	681		$^{L/D}$Y*X$_{8-15}$C*; $^{\beta}$G: β-homoglycine; $^{\beta}$A: β-homoalanine; $^{\beta}$X: β-homophenylglycine; $^{\beta}$Q: β-homoglutamine	^{L}Y*$^{\beta}$AN$^{\beta}$XLFND$^{\beta}$XFFL$^{\beta}$XPSC*	51	(67)
	47	66.8		Y*L$^{\beta}$XLY$^{\beta}$XRR$^{\beta}$XPNRYLYC*	7	
	ND			Y*F$^{\beta}$X$^{\beta}$XYNGFRSC*	4	
	1660			Y*$^{\beta}$AN$^{\beta}$XLFND$^{\beta}$X$^{\beta}$XL$^{\beta}$XPSC*	6	
	ND			^{D}Y*FFYDSS$^{\beta}$QVGVRYV$^{\beta}$QDC*	35	
	83.6			^{D}Y*VY ^{D}C*$^{\beta}$X$^{\beta}$GLDPRLSRFS	18	
	34.1	20.3		^{D}Y*FYY$^{\beta}$Q$^{\beta}$XVNVVYRR$^{\beta}$AC*	5	
	159	3290		^{D}Y*FYY$^{\beta}$GDDRVVYRV$^{\beta}$XC*	2	
Gαs/GNP	190	4150	^{D}Y*X$_{8-12}$C*	^{D}Y*FQSVYAIWGTLC*	1	(68)
	31.4	1150		^{D}Y*LITFRQWAFNLPC*	1	
K63-DiUb	16		$^{L/D}$Trp/pClBz/mClBz*X$_{10-15}$C*; mClBz: 3-(chloromethyl) benzoic acid; pClBz: 4-(chloromethyl) benzoic acid	mClBz*-LLIWIGSSKNPYILC*	ND	(69)

Table S1A. (Continued)

Screening Target	K_D (nM)	IC_{50} (nM)	Library design/ Note	Consensus motif/ Peptide sequence	variants	Reference
TNIK	6	0.13	Leader sequence-SWX$_{10-11}$SSSCAQPYPYDVPDYA	GFIYKTLKSSG	1	(70)
		4.6		WLGSQTYGCT	5	
	28	2		WLGDSAYGCS	5	
	58	2.5		WSYEHNGGNYM	1	
	53	2.7		WSQTHNQGHYM	1	
	6.3	0.6		AWSIAKGLNG	1	
		7.7		WSTRHTMGVA	1	
	25	9.1		WDIQIGMRFA	1	
	23			WHGWDKGYFG	1	
	9.7	1.7		WFGNKSLYRG	1	
	1.24	0.014		TIRTRGRIATC	1	
O-GlcNAc transferase		11	$^{L/D}$Y*X$_{7-12}$C*	^{L}Y*FC*PCYQIKYVKPS	11	(71)
		30		^{L}Y*FC*PKYQIIWTS	2	
		19		^{L}Y*FC*PQYQITYYRS	1	
		1.078		^{L}Y*WYTGKHRPRFYPC*	12	
				^{L}Y*VNRKPWWVYIRTC*	1	
		2.8		^{L}Y*RAPNTQAVWYC*	11	
				^{D}Y*WLYDETIYQWPC*	5	
				^{D}Y*LYYDEDGIHSLHC*	3	
		0.19		^{D}Y*IFTYQGLPKFAIC*	2	
		0.062		^{D}Y*WYTYRHRVVYGEC*	19	
				^{D}Y*WNQEDIWSFPC*	2	
α-Synuclein Amyloid Fibrils			$^{L/D}$Y*X$_{4-15}$C*	^{L}Y*NAALQILWLLITFLC*	ND	(72)
				^{L}Y*LTVLRWLLVLLTSCC*	ND	
				^{L}Y*KSAILVVKFVLQLIAC*	ND	
	4.74			^{D}Y*SGLIKWTTALLRTYC*	ND	
				^{D}Y*WSEIDSWLATVLC*V	ND	
				^{D}Y*VYC*FRTWCITEGLVC	ND	
				^{D}Y*DSLSC*IYWDAIVII	ND	
MRP1		Ki=100 nM	$^{L/D}$Y*X$_{4-15}$C*	^{D}Y*FWGNLHWYYEQFDSTC*	ND	(73)
mPro	2.3	40	^{D}Y*X$_{11-14}$^{D}C*; γ1: cis-3-aminocyclobutane carboxylic acid; γ2: (1R,3S)-3-aminocyclopentane carboxylic acid	^{D}Y*FHγ1FPPHTYTL ^{D}C*	10	(74)
	71	1750		^{D}Y*FHγ1FSIFRYRL ^{D}C*	1	
	62	2060		^{D}Y*LHYASTYSγ2YRYAA ^{D}C*	9	
	5.2	50		^{D}Y*FHγ1LNLGYRPG ^{D}C*	1	
	5.2	40		^{D}Y*FHγ2FRDLYILG ^{D}C*	1	
	82	2280		^{D}Y*FHSFAγ1LYTLG ^{D}C*	1	

Table S1A. (Continued)

Screening Target	K_D (nM)	IC_{50} (nM)	Library design/ Note	Consensus motif/ Peptide sequence	variants	Reference
mPro	490	7930		^{D}Y*LHFYTSγ2ATYIYP ^{D}C*	1	

Table S1B. Representative peptide sequences discovered by Phage display. Residues engaged in disulfide-macrocyclization are marked with asterisks.

Phage display				Diversity scale: 10^7–10^9	
Screening Target	K_D (nM)	IC_{50} (nM)	Library design/ Note	Consensus motif	Reference
NRP-1	<28.4 ±2.9		C*X_7C*	F-(F/Y)-XLRS	(75)
GPC3	284±33.51		X_{12} (Ph.D.-12)	DHLASLWWGTEL	(76)
PD-L1	510		X_{12} (Ph.D.-12)	NYSKPTDRQYHF	(77)
	1130			KHAHHTHNLRLP	
β-Catenin	4.57±0.57		AC#X6C#X6C#G<br>#: Three cysteines cyclized by chemical linker	AC#AQKLDGC#SYISWSC#G	(78)
	3.82±0.17			AC#SGWWPKC#QGYIPGC#G	
	3.89±0.32			AC#APGVYRC#NQNFIWC#G	
PDGFRβ		1400	X_{12} (Ph.D.-12)	IPLPPPSRPFFK	(79)
PKCδ	24.5 ± 3.0	0.9±0.05	X_{12} (Ph.D.-12)	$LX_2PXNXPX_4$	(80)
PTPRJ		ELISA absorbance:0.248	C*X_7C* (Ph.D.-C7C)	C*HHNLTHAC*	(81)
		ELISA absorbance:0.33		C*LHHYHGSC*	
PTPRJ		cell growth inhibition (%):<br>24 h: 48.0 ± 2.82<br>48 h: 62.5 ± 4.9<br>72 h: 66.5 ± 2.12	C*X_7C* (Ph.D.-C7C)	C*HHALTHAC*	(82)

Table S1B. (Continued)

Screening Target	K_D (nM)	IC_{50} (nM)	Library design/ Note	Consensus motif	Reference
TfR 1			X_{12} (Ph.D.-12)	SPRPRHTLRLSL	(83)
Tie 2	15800	4.4ug/ mL Pep-PEG-DDP-liposome	X_{12} (Ph.D.-12)	TMGFTAPRFPHY	(84)
CD-21	345		X_{15}	RMWPSSTVNLSAGRR	(85)
VEGFRI (Flt-1)	200	5000	X_{16}	$NX_2EIEXYXWX_5Y$	(86)
IL-10 RA		completely inhibited IL-10-induced STAT3 phosphorylation at 350nM	X_{12} (Ph.D.-12)	FRSFESCLAKSH	(87)
EGFR	22.28 ±0.4		X_{12} (Ph.D.-12)	YHWYGYTPQNVI	(88)
EGFR			X_{12} (Ph.D.-12)	QHYNIVNTQSRVa	(89)
EGFR	50		X_7 (Ph.D.-7)	QRHKPRE	(90)
FGF8b			X_7 (Ph.D.-7)	HSQAAVP	(91)
aFGF			X_7 (Ph.D.-7)	AGNWTPI	(92)
bFGF			X_7 (Ph.D.-7)	PLLQATL	(93)
IL-6Rα			X_7 (Ph.D.-7)	LSLITRL	(94)
α5β1			X_6	CRGDCL	(14)
		100		GACRGDCLGA	
α5β1			$C^*X_7C^*$	C*RRETAWAC*	(95)
				GAC*RRETAWAC*GA	
α6β1		8000	X_{15}	VSWFSRHRYSPFAVS	(96)
αvβ3/αvβ5			CX_9	CDCRGDCFC	(97, 98)
αvβ6		20	X_{12} (Ph.D.-12)	$RTDLX_2LRTYTL$	(99)

Table S1B. (Continued)

Screening Target	K_D (nM)	IC_{50} (nM)	Library design/ Note	Consensus motif	Reference
MMP-9		10000	C*X_{5-8}C*	C*X_2HWGFX_2C*	(100)
N-cadherin	10700		X_{12} (Ph.D.-12)	SWTLYTPSGQSK	(101)
E/N-cadherin	9400/323		X_{12} (Ph.D.-12)	SWELYYPLRANL	(102)
PSMA		2200	X_{12} (Ph.D.-12)	WQPDTAHHWATL	(103)
VEGFR-3			C*X_7C* (Ph.D.-C7C)	C*SDX_2HXWC*	(104)
VEGFR-3		174.8±31.1 µg/mL	X_{12} (Ph.D.-12)	WHWLPNLRHYAS	(105)
EGFRvIII/EGFR			X_{12} (Ph.D.-12)	WHTEILKSYPHE	(106)
				LPAFFVTNQTQD	
Carbonic anhydrase IX		1750±490	X_{12} (Ph.D.-12)	YNTNHVPLSPKY	(107)
EphA2	186±7		X_{12} (Ph.D.-12)	YSAYPDSVPMMS	(108)
EphB4		15	X_{12} (Ph.D.-12)	TNYLFSPNGPIARAW	(109)
PS			C*X_7C*	C*LSYYPSYC*	(110)
HER2	439±63		AC#X_6C#X_6C#G #: Three cysteines cyclized by chemical linker	AC#SLQDPNC#DWWGHYC#G	(111)
	304±10			AC#GLQGYGC#WGMYGKC#G	
HER2			X_{12} (Ph.D.-12)	CVGVLPSQDAIGIC	(112)
				CGPLPVDWYWC	
				CEWKFDPGLGQARC	
				CDYMTDGRAASKIC	
HER2	30200±7600		X_6	KCCYSL	(113)
	10.4		X_6	MARSGL	(114)
	9.1			MARAKE	
	164			MSRTMS	
HER2			X_{20}	WTGWCLNPEESTWGFCTGSF	(115)
HER2			X_{12} (Ph.D.-12)	MCGVCLSAQRWT	(116, 117)
				SGLWWLGVDILG	

Table S1B. (Continued)

Screening Target	K_D (nM)	IC_{50} (nM)	Library design/ Note	Consensus motif	Reference
TGA-72		420	X_{16}	NPGTCKDKWIECLLNG	(118)
		0.39		DPRHCQKRVLPCPAWL	
		10.32		FRERCDKHPQKCTKFL	
TGA-72			X_{16}	GGVSCMQTSPVCENNL	(119)
Galectin-3	72.2 ± 32.8		X_{16}	ANTPCGPYTHDCPVKR	(120)
	5.7 ± 2.2			PQNSKIPGPTFLDPH	
T antigen	69.9± 19.6		X_{15}	YYAWHWYAWSPKSV	(121, 122)
	321.9 ±70			HGRFILPWWYAFSPS	
T antigen	581.1 ±98.0	70±8	X_{15}	IVWHRWYAWSPASRI	(123)
Fibrin-fibronectin complexes			C*X_8C*	C*GLIIQKNEC*	(124)
				C*NAGESSKNC*	
FGFR	400		X_{26}	AESGDDYCVLVFTDSAWTKICDWSHFRN	(125)
FGFR	0.251		X_7 (Ph.D.-7)	MQLPLAT	(126)
FGFR			X_{15}	C-(R/S)-XLLXGAPFX$_4$C	(127)
E-selectin		200000	X_7 (Ph.D.-7)	IELLQAR	(128)
MMP2-processed collagen IV			X_7 (Ph.D.-7)	TLTYTWS	(129)
PSA	2.9±0.1	0.7	C*X_3CX_4CX_2C*	C*VAYCIEHHCWTC*	(130)
Notch1 NRR	150		AC#X_6C#X_6C#G<br>#: Three cysteines cyclized by chemical linker	AC#ERYQGC#FSVGGYC#G	(131)
CD44	460±50		X_7 (Ph.D.-7)	THENWPA	(132)

Table S1B. (Continued)

Screening Target	K_D (nM)	IC_{50} (nM)	Library design/ Note	Consensus motif	Reference
CD44	97.1 ± 6.89		X_{12} (Ph.D.-12)	WHPWSYLWTQQA	(133)
FGF3			X_7 (Ph.D.-7)	VLWLKNR	(134)
Extradomain-B fibronectin	11000		C*X_7C* (Ph.D.-C7C)	C*TVRTSADC*	(135)
APRIL			X_{12} (Ph.D.-12)	AAAPLAQPHMWA	(136)
p16			X_7 (Ph.D.-7)	SHSLLSS	(137)
pre-miR-21			X_{12} (Ph.D.-12)	ALWPPNLHAWVP	(138)
HER2			X_7 (Ph.D.-7)	LTVSPWY	(139, 140)
α-Enolase			X_{12} (Ph.D.-12)	SSMDIVLRAPLM	(141)
EGFR			X_{12} (Ph.D.-12)	FPMFNHWEQWPP	(142)
EGFR			X_7 (Ph.D.-7)	SYPIPDT	(143)
				HTSDQTN	
MUC18			C*X_7C*	C*LFMRLAWC*	(144)
Nucleolin		47800	Landscape phage- display libraries f8/8 and f8/9	DMPGTVLP	(145)
Nucleolin	1.3pM		Landscape phage- display libraries f8/8 and f8/9	DWRGDSMDS	(146)
				VPTDTDYS	
				VEEGGYIAA	
GP130b			X_{12} (Ph.D.-12)	VTWTPQAWFQWV	(147)
Nestin			X7 (Ph.D.-7)	AQYLNPS	(148)
Cadherins			C*X_7C*	C*SSRTMHHC*	(149)
α5β1			C*X_7C*	C*PIEDRPMC*	(150, 151)
αvβ6			X_{20} (Ph.D.-20)	RGDLATLRQLAQEDGVVGVR	(152, 153)
αvβ6			X_{12} (Ph.D.-12)	SPRGDLAVLGHK	(154)
				SPRGDLAVLGHKY	
αvβ3 (RMS-I)			C*$X_{7–10}$C*	C*QQSNRGDRKRC*	(155)
IL-13Rα2	3300		C*X_7C* (Ph.D.-C7C)	C*GEMGWVRC*	(156)
VPAC1		2700	X_{12} (Ph.D.-12)	GFRFGALHEYNS	(157)

Table S1B. (Continued)

Screening Target	K_D (nM)	IC_{50} (nM)	Library design/ Note	Consensus motif	Reference
IGHC			C*X_7C*	C*TLPHLKMC*	(158)
HSPGb			X_{12} (Ph.D.-12)	ASGALSPSRLDT	(159)
Adenoviral receptor			X_{12} (Ph.D.-12)	SWDIAWPPLKVP	(160)
GRP78			C*$_{3\text{-}12}$C*	C*VALPGGYVRVC*	(161)
GRP78			X_{12} (Ph.D.-12)	ETAPLSTMLSPY	(162)
GRP78			X_7 (Ph.D.-7)	GIRLRG	(163)
APP			C*X_7C*	C*PGPEGAGC*	(164)
IL-11Rα			C*X_7C*	C*GRRAGGSC*	(165)
PDGFRβb			C*X_7C*	CRGRRST	(166)
p32/gC1qR	3000		C*X_7C*	C*GNKRTRGC*	(167, 168)
TIP-1			X_7 (Ph.D.-7)	HVGGSSV	(169, 170)
α2bβ3			X_7 (Ph.D.-7)	RGDGSSV	(171)
α3β1b			X_7 (Ph.D.-7)	SWKLPPS	(172)
αvβ3<br>αvβ5	17.8 ± 8.6 /61.7 ± 13.3		C*X_7C*	C*RGDKRGPDC*	(173)
NRP-1	22200 ± 300		X_7 (Ph.D.-7)	GGKRPAR	(174)
	18800 ± 3200			RIGRPLR	
NRP-1	<28400±2900		C*X_7C*	C*GFYWLRSC*	(75)
NRP-1	28400 ±2900		X_7 (Ph.D.-7)	RX_2RX_2R	(175)
MMP2-processe collagen IV			X_7 (Ph.D.-7)	TLTYTWS	(129)
VAV3			X_7 (Ph.D.-7)	SSQPFWS	(176)
CRKL			$X_2CX_{14}CX_2$	YRCTLNSPFFWEDMTHECHA	(177)

Table S1B. (Continued)

Screening Target	K_D (nM)	IC_{50} (nM)	Library design/ Note	Consensus motif	Reference
Plectin-1			X_7 (Ph.D.-7)	KTLLPTP	(178)

Table S1C. Representative peptide sequences discovered by Yeast display.

Yeast display				Diversity scale: 10^7–10^9	
Screening target	K_D (nM)	IC_{50} (nM)	Library design/ Note	Consensus motif	Reference
Mcl-1	1.9±1		Bim-BH3 variants	RPEIWX$_2$QXLX$_2$LGDEINAYYAR	(179)
	1.5±1.2				
	2±1.2				
Mcl-1	20±14	K_i=4±1.8	Bim-BH3 variants	RPEIW(I/V)AQEIX$_3$GDE(V/F)NAYYAR	(180)
Bcl-XL	23±11	K_i=2.9±0.8			
Bfl-1	0.3		Bim-BH3 variants	RPEIWIAQELRRAGDVLNAYYAR	(181)
Bcl-XL	<0.1	K_i=0.09	Bim-BH3 variants	RPEIWYAQGLKRFGDEFNAYYAR	(182)
Bfl-1		K_i=15	Puma-BH3 variant	WAREIGAQLRRMADDLNAQYER	(183)
Bfl-1	3.2	K_i=300	Puma-BH3 variant	WAREIGAQLRRMADDLNAQYER	(184)
Mcl-1		K_i=0.6	Puma-BH3 variant WAREIGAQLRRMADDLNAQYER	RSQYEVIQELIRIGDIVLAYFER	
Bcl-XL		K_i=3.7		QPLIWFGAQLRRGADEFAAQRER	
TCR-42F3	3900		QL9 variant(X-P/Q/L-X$_6$-F/L/I/V)	SPLDSLWWI	(185)
	6400		QL9 variant(QLSX$_2$PX$_2$L)	QLSDRPVDL	
	3400		QL9 variant(X$_3$PFXFDI)	EENPFWFDI	
TCR-42F3	100	EC_{50}=2.86	QL9 variant(XPX$_6$-F/L/I/M)	WPAEGGFQL	(186)
	100	EC_{50}=70		SPAEAGFFL	
TCR55	500	EC_{50}=1200	HLA-B35 variant(XPX$_6$L/M/I/F/Y)	VPLTRDAEL	(187)
TCR2B4	2800		X$_8$	YVVVPDGT	(188)
	15400				
LplA		k cat/k m = 0.99 μM^{-1} min^{-1}	V/I/L/F/M-E/Q/D/H-XDKVX$_2$-E/Q/D/H-V/I/L/F/M-X-A/S	GFEIDKVWYDLDA	(189)

Table S1C. (Continued)

Screening target	K_D (nM)	IC_{50} (nM)	Library design/ Note	Consensus motif	Reference
Cetuximab	15.8±3.9		mD1 variant VQFDLSTRRLKC's point mutation, VX_{10}C	Q1V/D3N/S5G/T6I/K10R	(190)
αvβ3		K_i=2.5±0.6, IC_{50}=28±3	Lacticin 481 variant (AA19-24: WQFVF to X_6)	HVRGDN	(191)
αvβ3, αvβ5		26±5	EETI-II vatiant GCX_{2-3}RGDX_{6-5}CKQDSDCLAGCVCGPNGFCG	PRP-NPPLT	(192)
αvβ3, αvβ5αiibβ3, αvβ1		7±4,7±1,300±100	EETI-II vatiant GCPQGRDGWAPTSCSQDSDCLAGCVCX_3RGDX_{3-5}CG	REA- MPRT	(193)
αvβ3 Integrin	0.78±0.37		ArgP variant GCVRLHESCLGQQVPCCDPAATCYCX_2RGDX_4CYCR	SG- NDLV	(194)
αiibβ3	42–70		ArgP variant GCVRLHESCLGQQVPCCDPAATCYCX_2RGDX_4CYCR	YG- NDLR	(195)
αvβ3	20–30				
αvβ1, αvβ5	> 1000				
αiibβ3	60–90				
αvβ3, αvβ1, αvβ5	> 1000				
CTLA-4	3700		oMCoTI-II variant SAGGAX_2C(X_3)$_{2-4}$CRRDSDCX_3CICRGNGYC	PR-KYSHVP-PGK	(196)
Matriptase-1		K_i=0.83±0.1	oMCoTI-II variant (SGV)$_{50\%}$CP-K/R-X_4C-RR$_{50\%}$D$_{10\%}$SD$_{10\%}$CPGACIC(RGNGY)$_{10\%}$CG	WGVCPKVLRNCRRDSDCPGACICLGNGY	(197)
Trypsin		K_i=35.8±4.7			
Lysozyme	0.9±0.7		Gp2 variant MSNVNTGSLSVNDKKFWATVX_{6-8}FEVPVYAETLDEALELAEWQYX_{6-8}VTRVRP	FSYGNL--SGAYEY	(198)
Rabbit IgG	2.3±1.4			HSVHGY-GNALGY	
Goat IgG	0.2±0.1			YDYDADYY-YSNHSDYL	

Table S1C. (Continued)

Screening target	K_D (nM)	IC_{50} (nM)	Library design/ Note	Consensus motif	Reference
InsR	2.4±0.4		Gp2 variant MSNVNTGSLSVNDKKFWATVX$_{6-8}$FEVPVYAETLDEALELAEWQYX$_{6-8}$VTRVRP	DCLYNDTA-DPNYCI	(199)
Kras	31±6		aPP variant GPRRPRYPGDDAX$_3$DLX$_4$AXLX$_2$YLXVVA	SIE-HEYW-R-WN	(200)

Table S1D. Representative peptide sequences discovered by the One-Bead-One-Compound (OBOC) method. Residues engaged in disulfide-macrocyclization are marked with asterisks.

OBOC				Diversity scale: 10^5–10^7	
Screening target	K_D (nM)	IC_{50} (nM)	Library design/ Note	Consensus motif	Reference
Aminopeptidase N	37.5		F/Y/A/L-(V/E/I/K)$_2$-(N/R/L/D/G/S/H/Y)$_3$-C	Tyr-(Hydropgilic residue)$_2$-Tyr	(201)
α3β1 integrin			^{D}C*X$_6$^{D}C*	^{D}C*XGXGX$_2$^{D}C*	(202)
α3β1 integrin	500± 100		^{D}C*XGXGX$_2$^{D}C*	^{D}C*^{D}DGLG-Hyp-N^{D}C*	(203)
α3β1 integrin		57±0.004	^{D}C*^{D}DGXGX$_2$^{D}C* Hyp: hydrocyproline	^{D}C*^{D}DGXG-Hyp-N^{D}C*	(204)
α3β1 integrin		80±10	^{D}C*^{D}DGXG-Hyp-X^{D}C*X Hyp: hydrocyproline	^{D}C*^{D}DG-Phe(3,5-diF)-G-Hyp-N^{D}C*R	(205)
α4β1		2 pM	isocyanate-4-aminophentlacetic acid-L analog-D analog-V analog-X$_3$ Aad: α-Aminoadipic acid	2-methylphenyl urea-4-aminophenylacetic acid-(ε-6-(2E)-1-oxo-3-(3-pyridinyl-2-propenyl)-L-lysine)-(Aad)	(206)
αvβ3 integrin		680±80	^{D}C*X-R/K/Orn-G-D/E/Aad/Bec-X$_2$- ^{D}C* Orn: Ornithine; Aad: α-Aminoadipic acid; Bec: S-(2-boronoethyl)-L-cysteine	^{D}C*GXGD^{D}D^{D}X^{D}C*	(207)
αvβ3 integrin		70	^{D}C*GRGDX$_2$^{D}C*	^{D}C*GRGD^{D}DX^{D}C*	(208)
αvβ3 integrin	10.8± 1.2		X$_8$	(His/Lys)-X-(His/Lys)	(209)
CD21R	217		X$_7$	YILIHRN	(210)
CD21R	1000			PTLDPLP	
CD21R	476			LVLLTRE	
fungal giant unilamellar vesicles (GUVs)		MIC=25uM	X$_8$, X$_{10}$, X$_{12}$, X$_{14}$ Orn: Ornithine; Nva: Norvaline; Aib: 2-Aminoisobutyric acid	X-WW-Orn-N-Nva-Aib-RWW-XX	(211)

Table S1E. Representative peptide sequences discovered by *E.coli* display. Residues engaged in disulfide-macrocyclization are marked with asterisks.

E.coli display			Diversity scale: 10^7–10^9		
Screening target	K_D (nM)	IC_{50} (nM)	Library design	Candidate sequence/ Consensus motif	Reference
VEGF			X_{15}	X_6-W-(E/D)-W-(E/D)-X_9	(212)
VEGF	>50000		X_6-W-E/D-W-E/D-X_9	C-X_4-I/M-W-(E/D)-W-(E/D)-C-F/M	
VEGF				WWLW-(E/D)-W-(E/D)	
VEGF				XWWLX_2-W-(E/D)-W-(E/D)	
VEGF	470		X_4CX_4-I/M-W-E/D-W-E/D-C-F/I/L/M-X_3	CSR-F/L-V/L-MWEWECF	
HepG2			X_{12} (FliTrx)	IAVAPGWLWEEE	(213)
HepG2				KELCELDSLLRI	
HepG2				IRELYSYDDDFG	
PC-3 cell			X_{12} (FliTrx)	CPGDRGQRRLFSKIEGPC	(214)
PC-3M-1E8 cell			X_{12} (FliTrx)	NVVRQ	(215)
ZR-75-1 cell			X_{15} (containing X_2C*X_7C*X_2)	VEC*YLIRDNLC*IY	(216)
MDA-MB-231、MCF-7、T47-D cell			X_{15} (containing X_2CX_7CX_2)	EWC*GIVRVGYC*LGGGKK	(217)
SCC VII			X_{12} (FliTrx)	C*GGRRLGGC*	(218)
A549			X_{15} (containing X_2C*X_7C*X_2)	WFC*SWYGGDTC*VQ	(219)

Table S2. List of primers. The reverse primer for the library construction (NNK15.ex.R91) was purchased from Ajinomoto Bio-Pharma Services in OPC purification. Other primers are purchased from Eurofin Genomics custom DNA oligo synthesis service. Red codons in 10-4 and 12-4 related primers stand for the cysteine to serine mutation.

Name	Sequence (5'→3')
NNK15.ex.R91	AGTTAGTTAATCCGCCGGAACAMNNMNNMNNMNNMNNMNNMNNMNNM NNMNNMNNMNNMNNMNNMNNCATGGATATATCTCCTACTTAAAG
T7.F51	GGCGTAATACGACTCACTATAGGGTTGAACTTTAAGTAGGAGATATA TCCA
T7.F53	GGCGTAATACGACTCACTATAGGGTTGAACTTTAAGTAGGAGATATA TCCATG
LTN.R36	TTTCCGCCCCCCGTCCTAGTTAGTTAATCCGCCGGA
LTN.R39	TTTCCGCCCCCCGTCCTAGTTAGTTAATCCGCCGGAACA
12-4.C8, 17S.R91	AGTTAGTTAATCCGCCGGAGCTCCTACCATTCGGCAGAACAATCTTG CTCTCAACCCACAGACAATTCATGGATATATCTCCTACTTAAAG
12-4.C3, 17S.R91	AGTTAGTTAATCCGCCGGAGCTCCTACCATTCGGCAGAACAATCTTA CACTCAACCCACAGGCTATTCATGGATATATCTCCTACTTAAAG
12-4.C3, 8S.R91	AGTTAGTTAATCCGCCGGAACACCTACCATTCGGCAGAACAATCTTG CTCTCAACCCACAGGCTATTCATGGATATATCTCCTACTTAAAG
10-4.C14, 17S.R91	AGTTAGTTAATCCGCCGGAGCTACAATTGCTATTCCAAAGCTTACTA TTAAACCTATTCCACTCAATCATGGATATATCTCCTACTTAAAG
10-4.C16, 17S.R91	AGTTAGTTAATCCGCCGGAGCTGCTATTACAATTCCAAAGCTTACTA TTAAACCTATTCCACTCAATCATGGATATATCTCCTACTTAAAG
10-4.C14,16S.R91	AGTTAGTTAATCCGCCGGAACAGCTATTGCTATTCCAAAGCTTACTA TTAAACCTATTCCACTCAATCATGGATATATCTCCTACTTAAAG
aMD4.R88	AGTTAGTTAATCCGCCGGAGCAATCTAAGTTCCAGACTTCGTGGGTA CGGCGATTGAACTGACGCATGGATATATCTCCTACTTAAAG
efx.ext.R45	ACCTAACGCTAATCCCCTTTCGGGGCCGCGGAAATCTTTCGATCC
efx.ext.F36	GTAATACGACTCACTATAGGATCGAAAGATTTCCGC
tRNA$^{fMet}_{CAU}$.ext.F68	GTAATACGACTCACTATACGCGGGGTGGAGCAGCCTGGTAGCTCGTC GGGCTCATAACCCGAAGATCG
tRNA$^{fMet}_{CAU}$.ext.R46	TGGTTGCGGGGGCCGGATTTGAACCGACGATCTTCGGGTTATGAGC
efx.amp.F22	GGCGTAATACGACTCACTATAG
efx.amp.R18	ACCTAACGCTAATCCCCT
tRNA$^{fMet}_{CAU}$.amp.F22	GGCGTAATACGACTCACTATAC
tRNA$^{fMet}_{CAU}$.ext.R17	TGmGTTGCGGGGGCCGGA (Gm indicates 2'-O-methylguanosine.)

Table S3. Top 100 peptide sequences of MET-binders after the 7th round of selection. The 1st Tyrosine was assigned as ClAc-^{L}Tyrosine. The sequences initiated by residues other than ClAc-^{L}Tyrosine, together with similar sequences, were removed from positive clone. The linker sequence, SGGLTN, following the 17th cysteine, is omitted from the peptide sequence.

10^6 Library Top 20 sequences

Name	Sequence	Read number	Population
Removed	YMLFWSAFESNKWNAIC	56652	37.04%
6-1	YSWYLNWQAQWKKFEFC	39966	26.13%
Removed	KVLFWSAFESNKWNAIC	39365	25.74%
6-2	YFYYNWTNVAKWAKTLC	3065	2.00%
	YYMTYQQNAYIPRHLLC	2327	1.52%
	TVLFWSAFESNKWNAIC	1442	0.94%
	KVLFWSDFESNKWNAIC	1339	0.88%
6-3*	YYIIYHAPLGWIKYLSC	791	0.52%
6-4*	YRYFFVIRNRLVLTFHC	425	0.28%
	YMLFWSAFESNKWTAIC	346	0.23%
	YFSMKISKSGNLLWKQC	295	0.19%
	IMLFWSAFESNKWNAIC	285	0.19%
	YKIWVFVNVRIFVLHQC	210	0.14%
	YKVYVSKTWNFVVIITC	173	0.11%
6-5*	YFVVNNITGLVITLIKC	154	0.10%
	KVLFWSAFESNKWIAIC	147	0.10%
	YMLFWSAFESNKWIAIC	127	0.08%
	YYMTYQQNAYIPRDLLC	124	0.08%
	YLVIIRSYSTWTIFKQC	108	0.07%
	YIIKI(Stop)ATTALWLILPC	107	0.07%
6-6*	YNFVLLYGTKFLSLRNC	43	0.03%

*Additional peptide synthesized and analyzed at the request of the editor. Also see Table S5 and Figure S10.

Table S3. (Continued)

10^8 Library Top 20 sequences

Name	Sequence	Read number	Population
8-1	YWYYTFDGRWKEYGAAC	44745	42.40%
8-2	YYHWNGRDGDKVEWVVC	14436	13.68%
Removed	KVLFWSAFESNKWNAIC	13206	12.51%
8-4	YSWYLNWQAQWKKFEFC	9248	8.76%
SPPS failed (8-5*)	YWYYTFDGRWEEYGAAC	8364	7.93%
8-3	YWYYTFDGRWEEYDAAC	5578	5.29%
	YNSMFVYGLDDWKKYTC	1818	1.72%
	KVLFWSAFESNKWTAIC	918	0.87%
	YWYYTFDRRWEEYGAAC	838	0.79%
	YFIRNGWRHTEITSMCC	807	0.76%
	YMLFWSAFESNKWNAIC	534	0.51%
	TVLFWSAFESNKWNAIC	482	0.46%
	KVLFWSAFESNKWIAIC	276	0.26%
	YYYYDYTCNYVWLEDLC	265	0.25%
	YWYYTFDGRWKKYTC	196	0.19%
	YWFYNIVQCCSWYYFKC	140	0.13%
	YWYYTFDGRWEEYCAAC	137	0.13%
	YMLFWSAFESNKWTAIC	126	0.12%
	YWYYRFDGRWEEYGAAC	116	0.11%
	KVLFWSAFESNKWKAIC	106	0.10%

*The peptide was re-synthesized and analyzed per the request of the editor. Also see Table S5 and Figure S10.

Table S3. (Continued)

10^{10} Library Top 20 sequences

Name	Sequence	Read number	Population
10-1	YYSWSGSVSESVKWNSC	162330	69.85%
10-2	YWYYTFDGRWKEYGAAC	14156	6.09%
10-3	YYHWNGRDGDKVEWVVC	10668	4.59%
10-4	YIEWNRFNSKLWNCNCC	8752	3.77%
10-5	YLLWHKYDNKDWVYVDC	8699	3.74%
10-6	YWWYYWYRDEYLWRTRC	8256	3.55%
	YYSWSGSVSESVKWNNC	4427	1.90%
	YYSWSGNVSESVKWNSC	2741	1.18%
	KVLFWSAFESNKWTAIC	2497	1.07%
	YYILNSYPTFTVWNFRC	1818	0.78%
	YLDWMTFDSKDWLVNDC	925	0.40%
	YTYLSPSPFHSVWDYEC	475	0.20%
	YVYIGEKPAFEIIAYSC	475	0.20%
	YYSWSGSVSESVKWNRC	330	0.14%
	YYYWSGRDPDPVQYENC	302	0.13%
	YYSWSGRVSESVKWNSC	300	0.13%
	YWYYTFNQIWIELKSHC	274	0.12%
	YYSWSGSVSESVKWNTC	271	0.12%
	YWWYYWYRDEYLWKTRC	185	0.08%
	YIFWLVYDSPNWQILNC	183	0.08%

Table S3. (Continued)

Top 20 sequences in E12 library

Name	Sequence	Read number	Population
12-1	YISWFAYASKDWFVRPC	110557	22.77%
12-2	YYSWSGNVSESVKWNSC	97053	19.99%
SPPS failed	YSCYWVYCQKVRPDGVC	77691	16.00%
12-3	YYSWSGSVSESVKWNSC	37943	7.82%
12-4	YNCLWVECKIVLPNGRC	25756	5.31%
SPPS failed	YLSWNDYNSDKWFYTPC	21581	4.45%
12-5	YLVWHEYDSKKWYFSLC	20078	4.14%
	YLFWKEFNSAQWVVVKC	14995	3.09%
	YYSWSGNVYEDVNWKEC	12042	2.48%
	YFYLHCINECEWKRQPC	10333	2.13%
	YWWYKCISECVWSWTKC	7208	1.48%
	YLEWSTYESSLWNFVMC	6285	1.29%
	YWYYTFDRRWVPFC	5372	1.11%
	YISWADYDSLNWYITRC	3826	0.79%
	YIGWKRFDSQEWYITNC	2977	0.61%
	YRFFYYWFRGKYVKLAC	2778	0.57%
	YWILHIDGYSWKNYAC	2466	0.51%
	YIHVYGKPEFIVQQYRC	2203	0.45%
	YIKWTAYDSKQWFFVQC	2094	0.43%
	YYFYYGLSGWVPLGRYC	1788	0.37%

Table S3. (Continued)

Top 20 sequences in the E14 library

Name	Sequence	Read number	Population
14-1	YWVYGFGKLNNDWRAFC	27219	13.84%
14-2	YYSWSGKDVDSVIWNNC	25421	12.93%
14-3	YLSWSEFDSKNWSLAYC	6226	3.17%
14-4	YYSWTGRDFDAVKWNKC	6006	3.05%
14-5	YLSWKYYDSSDWVILSC	5957	3.03%
14-6	YIEWYVYDSKSWVYVRC	4070	2.07%
	YYFWSGRDSDPVRWQNC	4059	2.06%
	YNCCFIWNITNWLLVPC	4027	2.05%
	YIGWNRYDSREWFLLDC	3490	1.77%
	YYVWSGKDSDIVIWIVC	3482	1.77%
	YLTWDDFDSNEWYITSC	2578	1.31%
	YIRWLTFDSKDWNFVNC	2511	1.28%
	YIEWANYNSKMWKYIRC	2347	1.19%
	YIAWNRWNSKDWFYVDC	2184	1.11%
	YLRWNAFNSKNWFVDAC	1952	0.99%
	YYRWNGKLDSDIEWIGC	1874	0.95%
	YLQWNNYDSRDWSIRPC	1710	0.87%
	YYSWSGNVSESVKWNSC	1692	0.86%
	YITWTDWDSSDWYFVNC	1672	0.85%
	YLNWKTWDSKSWIYVNC	1651	0.84%

Table S4. Read number and ratio of discovered sequence families and unique sequences at each round for (A) E6, (B) E8, (C) E10, (D) E12, and (E) E14 library.

A

**Peptide family/ Peptide**	**Selection cycles (Read number/ Population ratio)**						
	**Round1**	**Round2**	**Round3**	**Round4**	**Round5**	**Round6**	**Round7**
Family I	0<br>< 0.01%	0<br>< 0.01%	5<br>< 0.01%	0<br>< 0.01%	0<br>< 0.01%	0<br>< 0.01%	0<br>< 0.01%
Family II	0<br>< 0.01%	0<br>< 0.01%	3<br>< 0.01%	0<br>< 0.01%	3<br>< 0.01%	10<br>< 0.01%	50<br>0.03%
Family III	0<br>< 0.01%	0<br>< 0.01%	2<br>< 0.01%	1<br>< 0.01%	6<br>< 0.01%	13<br>< 0.01%	7<br>< 0.01%
Family IV	1<br>< 0.01%	1<br>< 0.01%	3<br>< 0.01%	390<br>0.12%	13799<br>5.94%	50841<br>24.33%	39967<br>26.13%
6-1	1<br><0.01%	1<br><0.01%	3<br><0.01%	390<br>0.12%	13799<br>5.94%	50841<br>24.33%	39967<br>26.13%
6-2	0<br>< 0.01%	0<br>< 0.01%	0<br>< 0.01%	0<br>< 0.01%	6<br>< 0.01%	234<br>0.11%	3065<br>2.00%
**Total read number of E6 library**	76044	98548	126338	321078	232391	208998	152934

Table S4. (Continued)

B

Peptide family/ Peptide	Selection cycles (Read number/ Population ratio)						
	**Round1**	**Round2**	**Round3**	**Round4**	**Round5**	**Round6**	**Round7**
Family I	0<br><0.01%	0<br><0.01%	5<br><0.01%	8<br><0.01%	79<br>0.07%	3366<br>1.60%	14436<br>13.68%
Family II	0<br><0.01%	0<br><0.01%	1<br><0.01%	0<br><0.01%	0<br><0.01%	0<br><0.01%	0<br><0.01%
Family III	5<br><0.01%	15<br>0.01%	687<br>0.47%	20174<br>12.41%	43300<br>40.53%	104847<br>49.82%	58778<br>55.69%
Family IV	0<br><0.01%	3<br><0.01%	121<br>0.08%	8390<br>5.16%	21488<br>20.11%	50762<br>24.12%	9249<br>8.76%
8-1	1<br><0.01%	1<br><0.01%	3<br><0.01%	92<br>0.06%	1279<br>1.20%	29843<br>14.18%	44745<br>42.40%
8-2	0<br><0.01%	0<br><0.01%	0<br><0.01%	8<br><0.01%	79<br>0.07%	3366<br>1.60%	14436<br>13.68%
8-3	4<br><0.01%	14<br>0.01%	684<br>0.47%	20113<br>12.37%	42130<br>39.43%	75441<br>35.85%	14094<br>13.35%
8-4	0<br><0.01%	3<br><0.01%	121<br>0.08%	8390<br>5.16%	21488<br>20.11%	50762<br>24.12%	9249<br>8.76%
**Total read number of E8 library**	146898	134509	145821	162598	106839	210452	105537

Table S4. (Continued)

C

Peptide family/ Peptide	Selection cycles (Read number/ Population ratio)						
	**Round1**	**Round2**	**Round3**	**Round4**	**Round5**	**Round6**	**Round7**
Family I	0	13	895	9103	51421	112426	181950
	<0.01%	0.01%	0.80%	15.47%	57.41%	72.24%	78.29%
Family II	0	3	294	1600	5523	10971	18427
	<0.01%	<0.01%	0.26%	2.72%	6.17%	7.05%	7.93%
Family III	0	91	6865	29404	15808	17374	14334
	<0.01%	0.05%	6.16%	49.96%	17.65%	11.16%	6.17%
Family IV	1	45	2018	2517	381	54	8
	<0.01%	0.03%	1.81%	4.28%	0.43%	0.03%	<0.01%
10-1	0	1	315	4624	36603	93311	162330
	<0.01%	<0.01%	0.28%	7.86%	40.87%	59.95%	69.85%
10-2	0	60	5800	27581	15121	16996	14156
	<0.01%	0.04%	5.21%	46.87%	16.88%	10.92%	6.09%
10-3	0	12	577	4388	13657	15330	10668
	<0.01%	0.01%	0.52%	7.46%	15.25%	9.85%	4.59%
10-4	0	1	75	462	1782	4266	8752
	<0.01%	<0.01%	0.07%	0.79%	1.99%	2.74%	3.77%
10-5	0	1	136	856	2988	5997	8699
	<0.01%	<0.01%	0.12%	1.45%	3.34%	3.85%	3.74%
10-6	0	0	72	488	955	3319	8256
	<0.01%	<0.01%	0.06%	0.83%	1.07%	2.13%	3.55%
**Total read number of E10 library**	147914	169006	111410	58850	89562	155638	232411

Table S4. (Continued)

D

Peptide family/ Peptide	Selection cycles (Read number/ Population ratio)						
	**Round1**	**Round2**	**Round3**	**Round4**	**Round5**	**Round6**	**Round7**
Family I	0	12	64780	39561	38091	33717	137651
	<0.01%	0.01%	44.52%	36.95%	41.40%	25.09%	28.36%
Family II	0	103	9615	20375	26028	42145	135424
	<0.01%	0.06%	6.61%	19.03%	28.29%	31.36%	27.90%
Family III	0	0	844	253	67	45	17
	<0.01%	<0.01%	0.58%	0.24%	0.07%	0.03%	<0.01%
Family IV	0	0	21	1	0	0	0
	<0.01%	<0.01%	0.01%	<0.01%	<0.01%	<0.01%	<0.01%
12-1	0	1	3537	10828	20319	35107	110557
	<0.01%	<0.01%	2.43%	10.11%	22.08%	26.12%	22.77%
12-2	0	1	8211	9962	13672	18799	97053
	<0.01%	<0.01%	5.64%	9.30%	14.86%	13.99%	19.99%
12-3	0	7	55097	28989	23894	14385	37943
	<0.01%	<0.01%	37.87%	27.07%	25.97%	10.70%	7.82%
12-4	0	1	1771	2557	2740	8139	25756
	<0.01%	<0.01%	1.22%	2.39%	2.98%	6.06%	5.31%
12-5	0	0	1285	3394	3729	5340	20078
	<0.01%	<0.01%	0.88%	3.17%	4.05%	3.97%	4.14%
**Total read number of E12 library**	157020	171560	145499	107071	92017	134381	485439

Table S4. (Continued)

E

Peptide family/ Peptide	Selection cycles (Read number/ Population ratio)						
	**Round1**	**Round2**	**Round3**	**Round4**	**Round5**	**Round6**	**Round7**
Family I	0	991	3142	6467	18285	17294	34268
	<0.01%	0.64%	1.53%	5.26%	10.78%	12.78%	17.42%
Family II	1	240	12931	17641	27986	22440	29267
	<0.01%	0.15%	6.30%	14.34%	16.50%	16.58%	14.88%
Family III	0	4	0	0	0	0	0
	<0.01%	<0.01%	<0.01%	<0.01%	<0.01%	<0.01%	<0.01%
Family IV	0	3	1	0	0	0	0
	<0.01%	<0.01%	<0.01%	<0.01%	<0.01%	<0.01%	<0.01%
14-1	0	0	538	1401	4200	10971	27219
	<0.01%	<0.01%	0.26%	1.14%	2.48%	8.11%	13.84%
14-2	0	0	891	3870	12819	12383	25421
	<0.01%	<0.01%	0.43%	3.15%	7.56%	9.15%	12.93%
14-3	0	0	572	1167	2230	3344	6226
	<0.01%	<0.01%	0.28%	0.95%	1.31%	2.47%	3.17%
14-4	0	0	247	682	2345	2662	6006
	<0.01%	<0.01%	0.12%	0.55%	1.38%	1.97%	3.05%
14-5	0	0	483	1169	2607	3886	5957
	<0.01%	<0.01%	0.24%	0.95%	1.54%	2.87%	3.03%
14-6	0	0	342	687	1564	4121	4070
	<0.01%	<0.01%	0.17%	0.56%	0.92%	3.05%	2.07%
**Total read number of E14 library**	194518	155936	205265	123023	169630	135321	196679

Table S5.* Comparison of median values of K_D among E6-14 libraries. P-values are calculated using Mann–Whitney U test for peptides' K_D listed in Table 1 and additionally synthesized peptide 6-3's first measurement data (Figure S10). The significant improvement K_D can be observed between E6-E12, and E6-E14 libraries. It should be note that the additionally synthesized peptides (Figure S10) would not be selected in standard RaPID selection since their abundances at Round 7 all below 2%.

	E6	E8	E10	E12	E14
(vs. E6)					
(vs. E8	0.7213				
(vs. E10)	0.5476	0.7476			
(vs. E12)	**0.0358**	**0.0651**	0.1423		
(vs. E14)	**0.0238**	0.2571	0.3939	0.9271	

*These additional analyses were performed at the request of the editor to ensure the robustness of our dataset. While these results provide a statistical difference between libraries, we did not discuss about them in detail in the manuscript because peptide 6-3 was selected from those that did not meet the hit criteria (the abundance at Round 7 >2%).

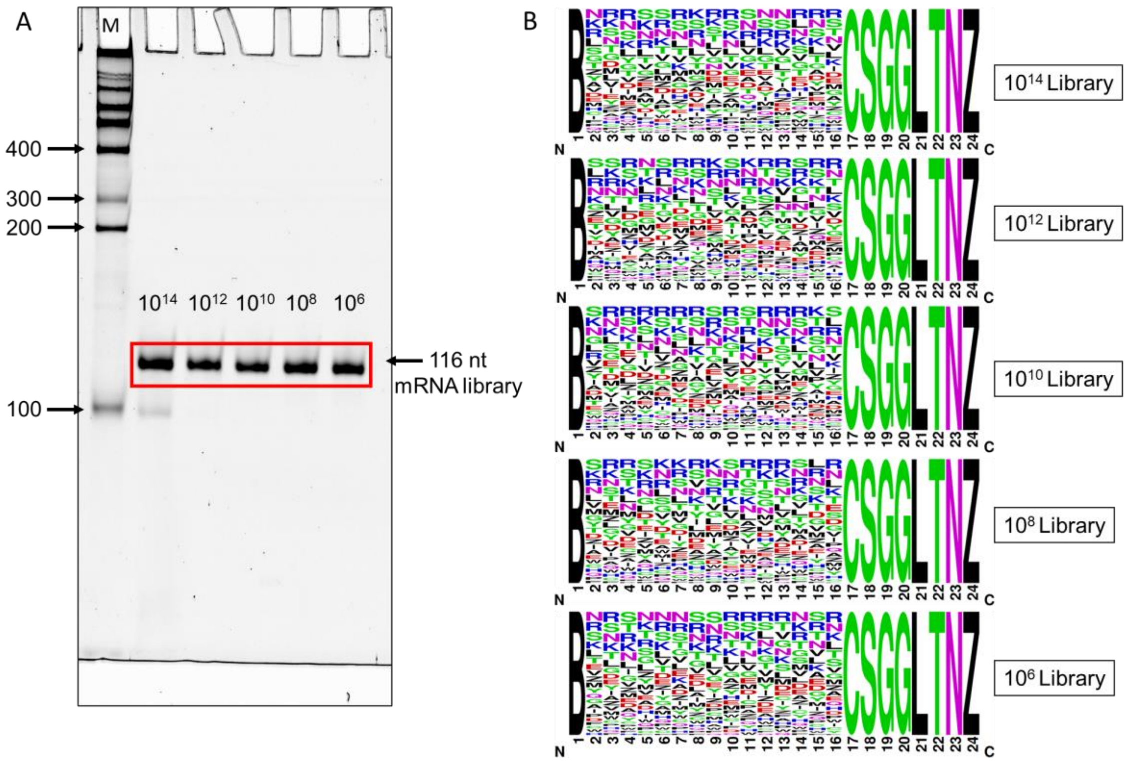

Figure S1. Quality validation of mRNA libraries by gel electrophoresis and sequencing. (A) The nucleotide length of each mRNA library was analyzed by 8% Urea-PAGE after staining using Ethidium Bromide (EtBr). All libraries have the same length of 116 bp. M: length marker. (B) The prepared mRNA libraries were sequenced by NGS and visualized using WEBLOGO 2.8.2. The height of the symbols in each residue spot indicates the probability of each amino acid's appearance in the library. The start and stop codons are designated as B and Z, respectively. The chart suggests that the amino acid set is fixed at residues 1 and 17-24, while at residues 2-16, the well-mixed amino acid symbols suggest no significant codon bias.

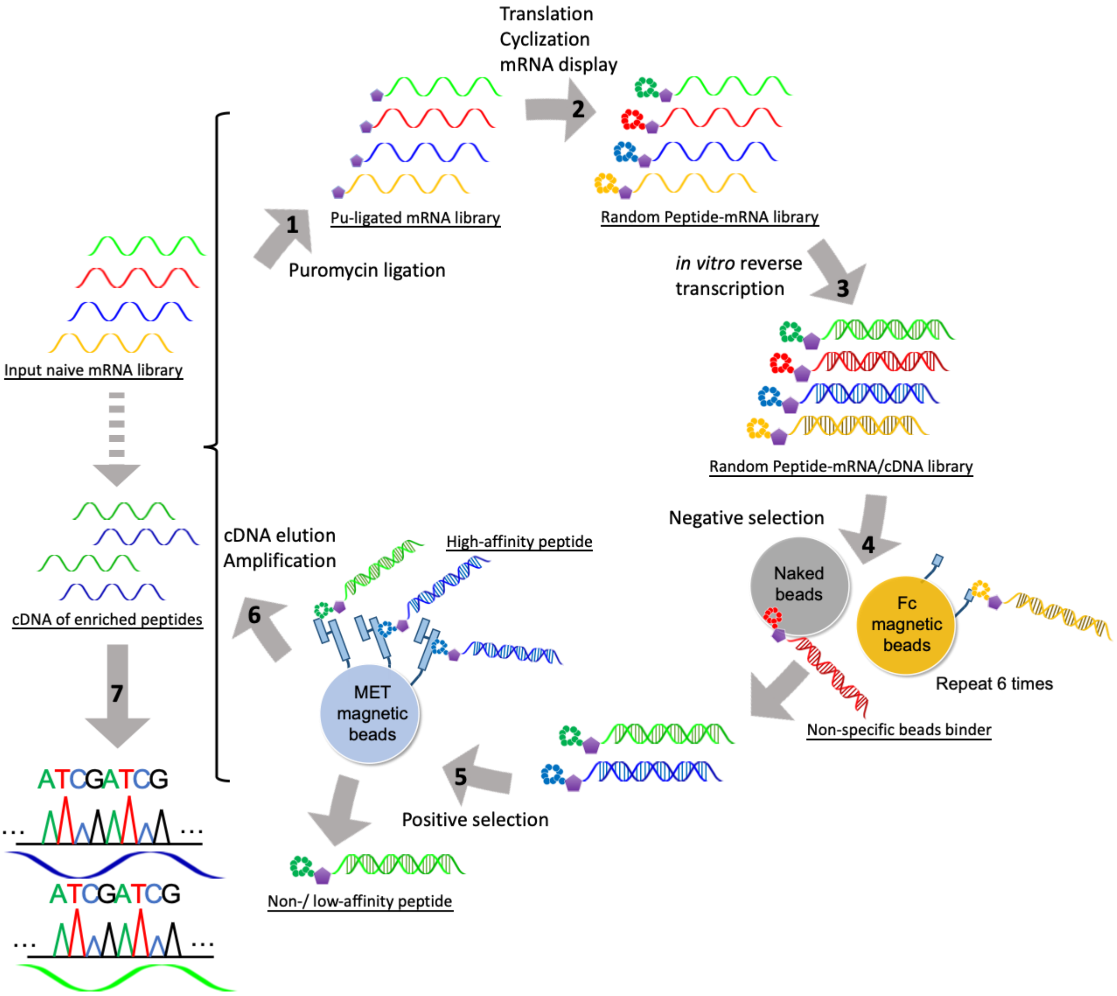

Figure S2. Schematic representation of the selection. From mRNA library, (1) ligation with puromycin linker, (2) translation, cyclization, and mRNA display to the peptide, (3) reverse transcription, (4) negative selection (from the second round), (5) positive selection, and (6) recovered cDNA was amplified and transcribed to give mRNA library for the next round. After repeating several selection rounds, (7) cDNA library encoding binder sequences are analyzed using the next-generation sequencer.

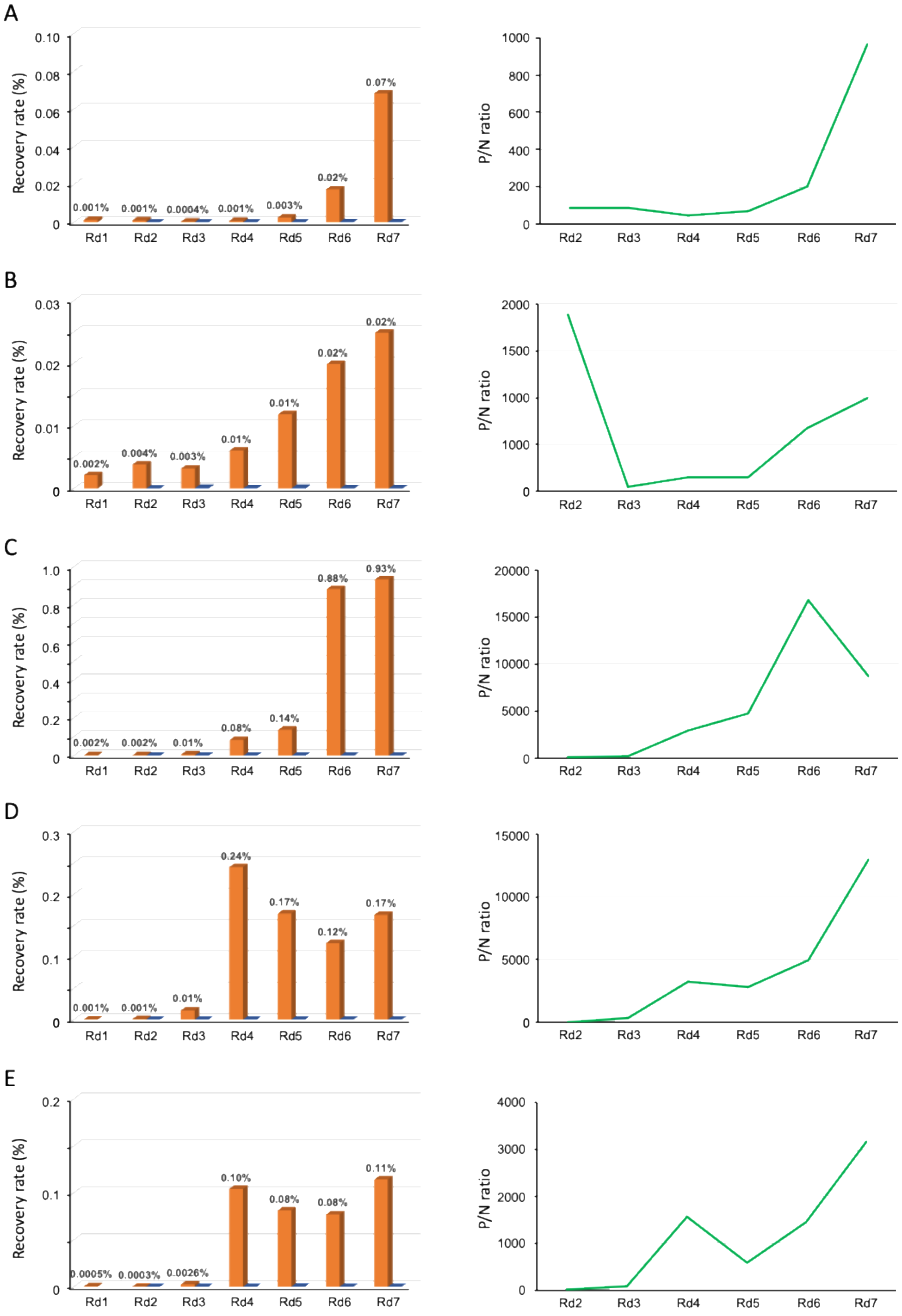

Figure S3. Recovery rate of cDNA at each round. Results for (A) E6, (B) E8, (C) E10, (D) E12, and (E) E14 library. Orange and blue bars indicate the recovery rate of MET binders (positive) and beads binders (negative), respectively. Negative selection was not performed in the first round. The graphs shown on the right by green lines indicate the ratio of positive recovery/negative recovery (P/N) at each round.

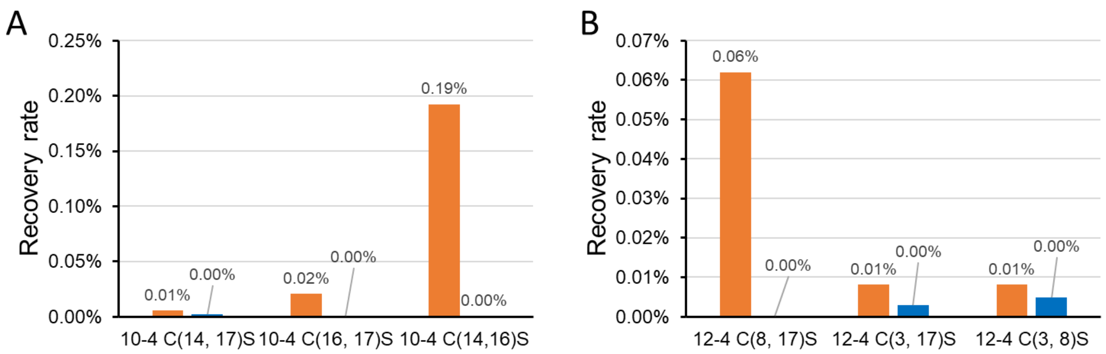

Figure S4. The recovery rate of the mutants of hit peptides having multiple cysteines. Recovery rates of each mutant for (A) 10-4 and (B) 12-4. According to the recovery rate, the cyclization position of 10-4 and 12-4 is the 17th and the 3rd cysteine, respectively.

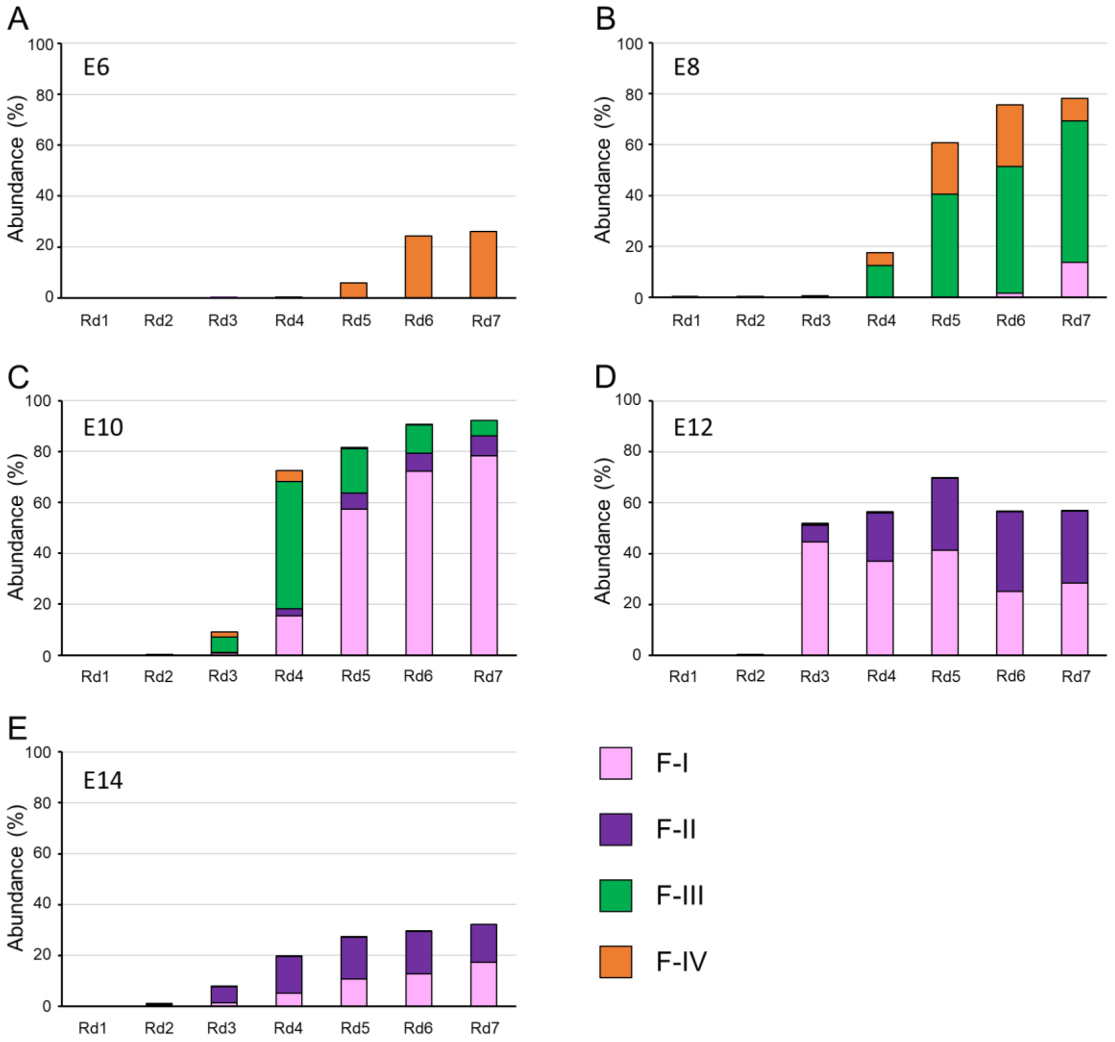

Figure S5. Population of peptide families after each selection round. The abundance of each family was analyzed for (A) E6, (B) E8, (C) E10, (D) E12, and (E) E14 library. Pink, purple, green, and orange color codes are used for F-I, F-II, F-III, and F-IV, respectively.

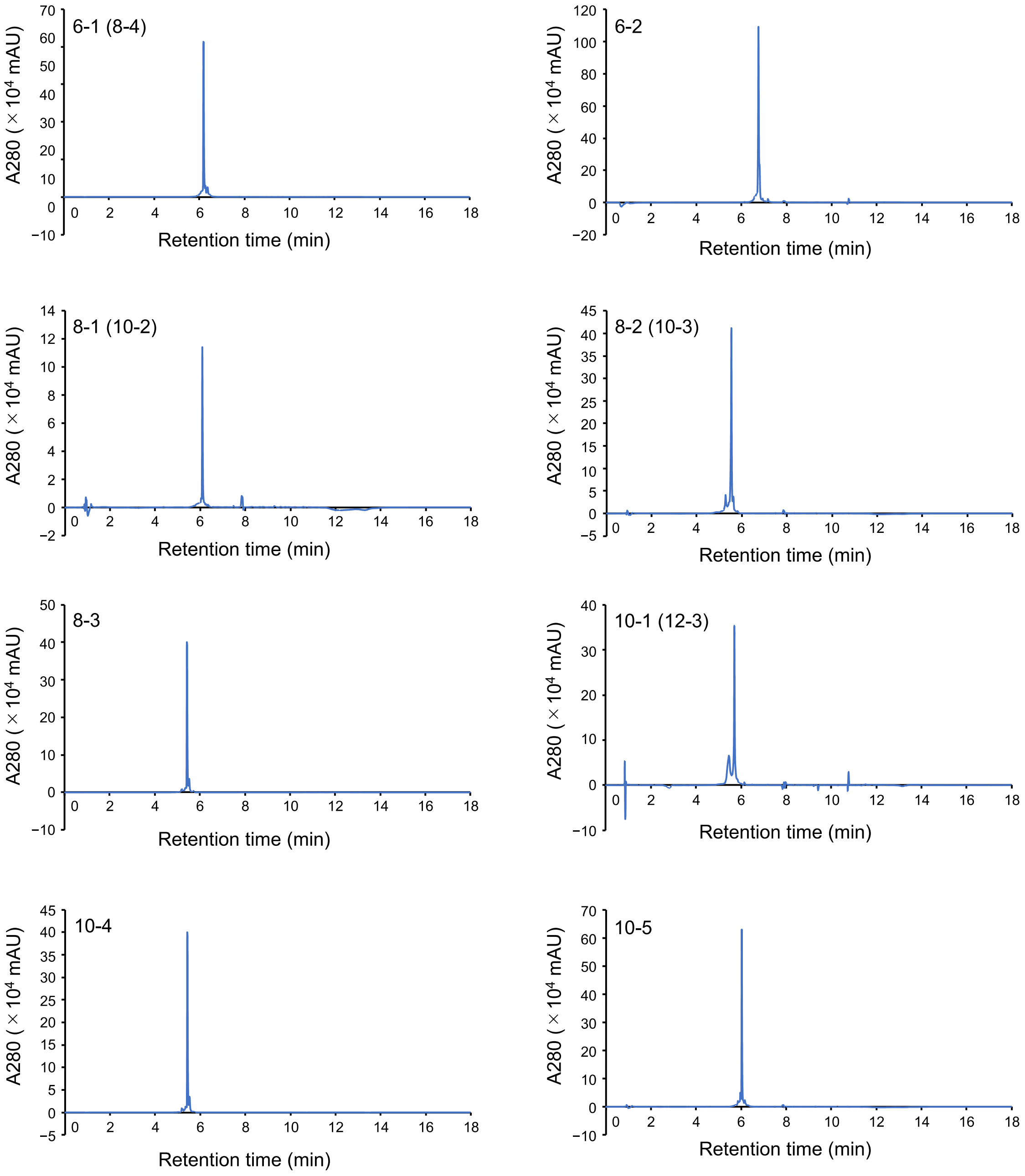

Figure S6. Chromatograms of synthesized macrocyclic peptides measured by UPLC. The analyses were performed under a linear gradient condition using two solvents; buffer A was water with 0.1% (v/v) TFA; buffer B was acetonitrile with 0.1% (v/v) TFA.

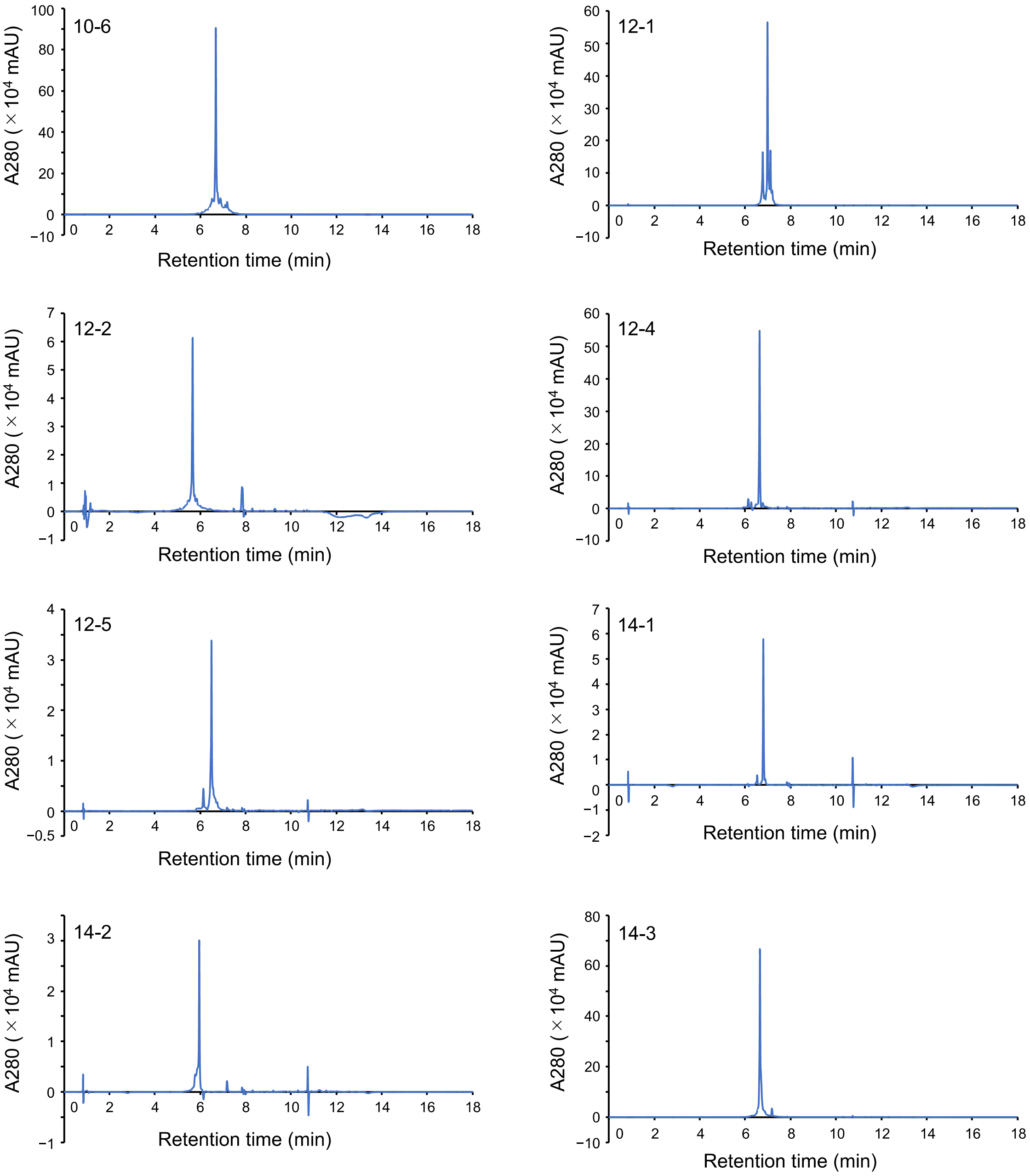

Figure S6. (Continued)

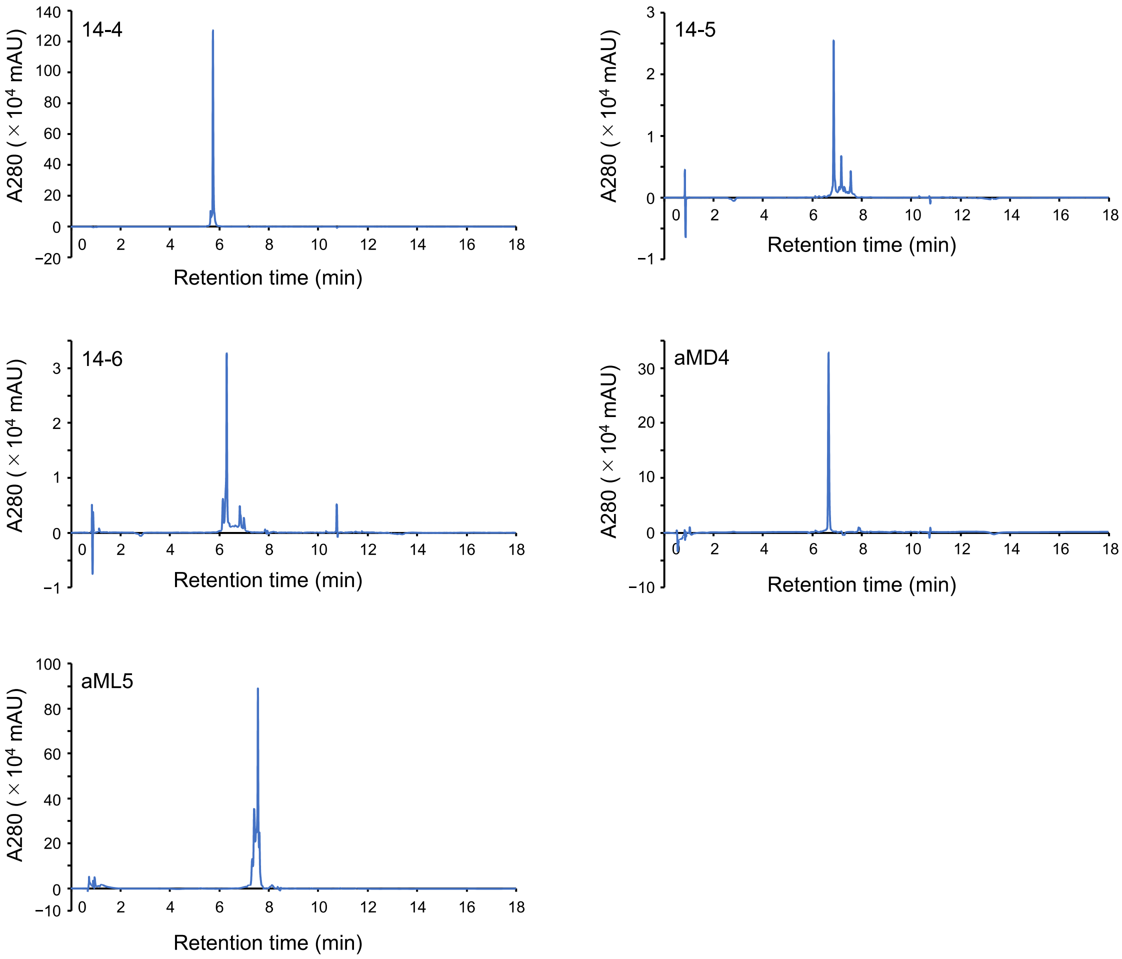

Figure S6. (Continued)

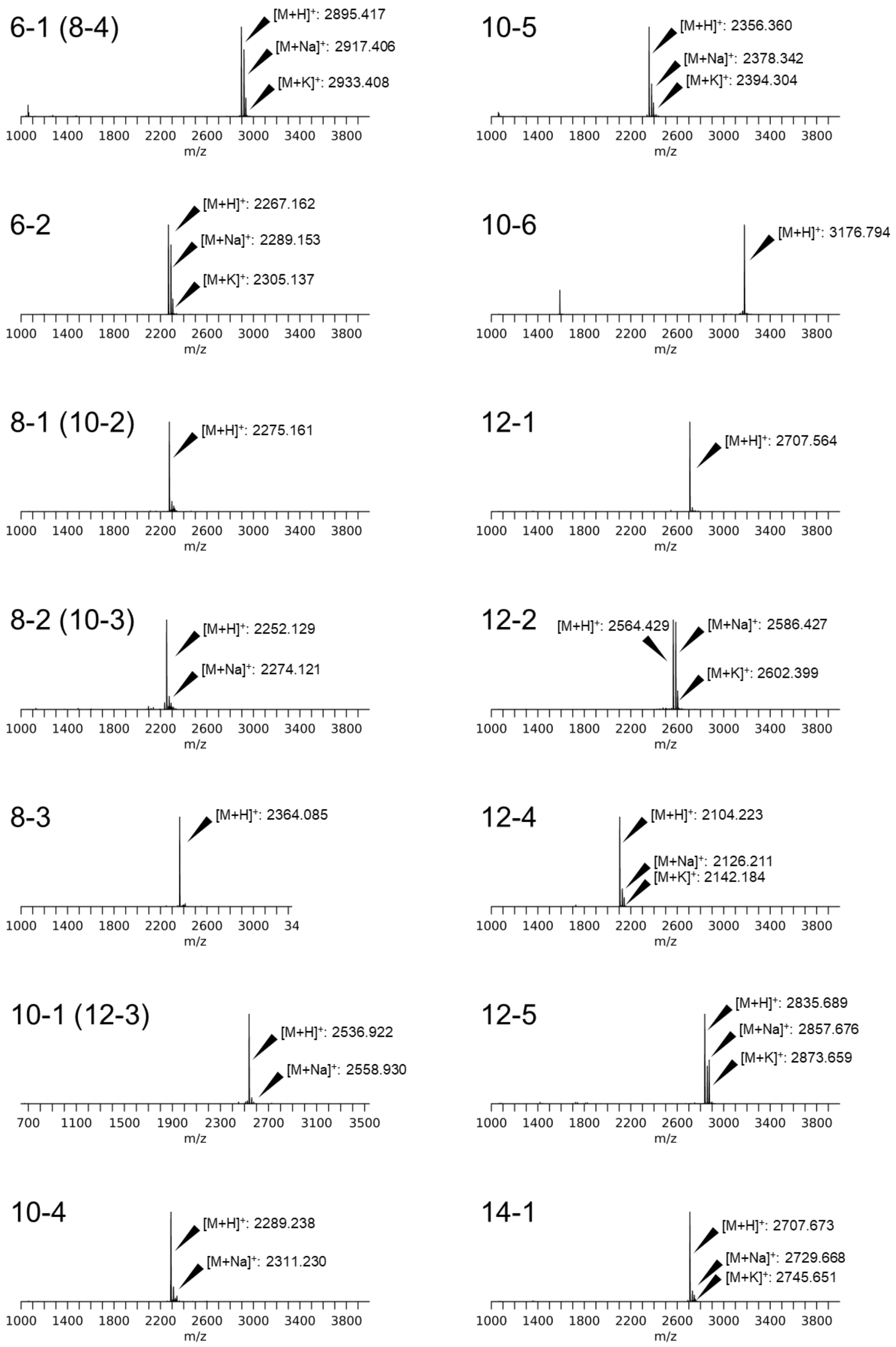

Figure S7. MALDI-TOF mass data of synthesized peptides. The peaks of $[M+H]^+$, $[M+Na]^+$, and $[M+K]^+$ indicate the proton, sodium cation, and potassium cation adducts of the desired peptide, respectively.

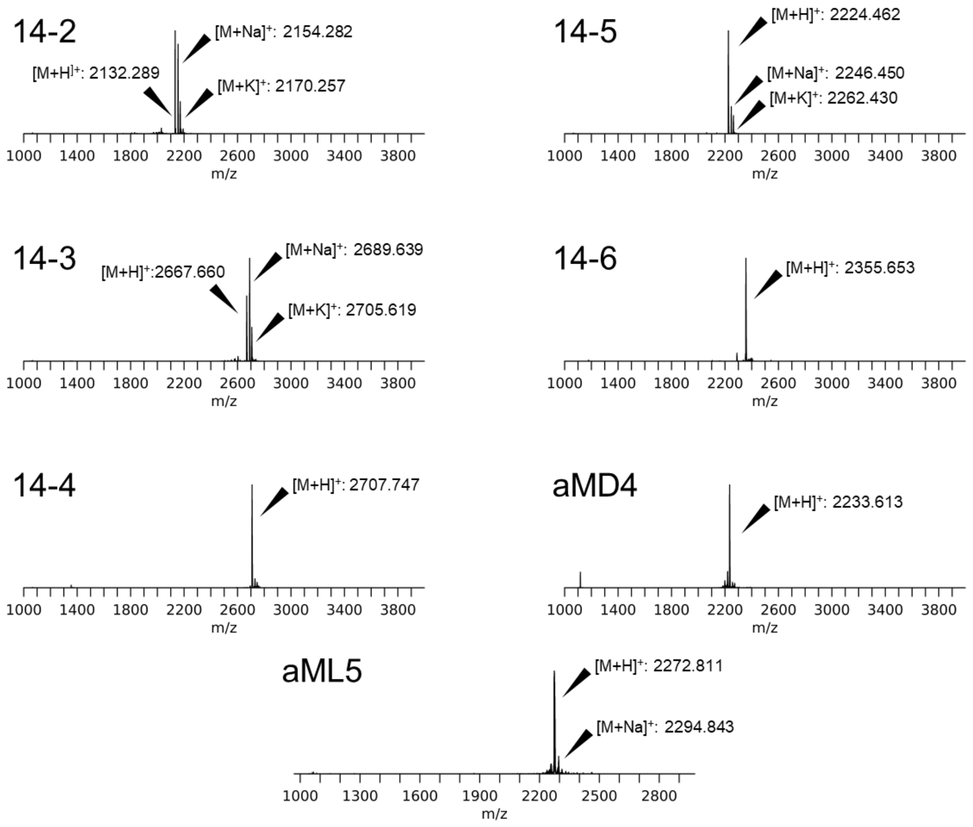

Figure S7. (Continued)

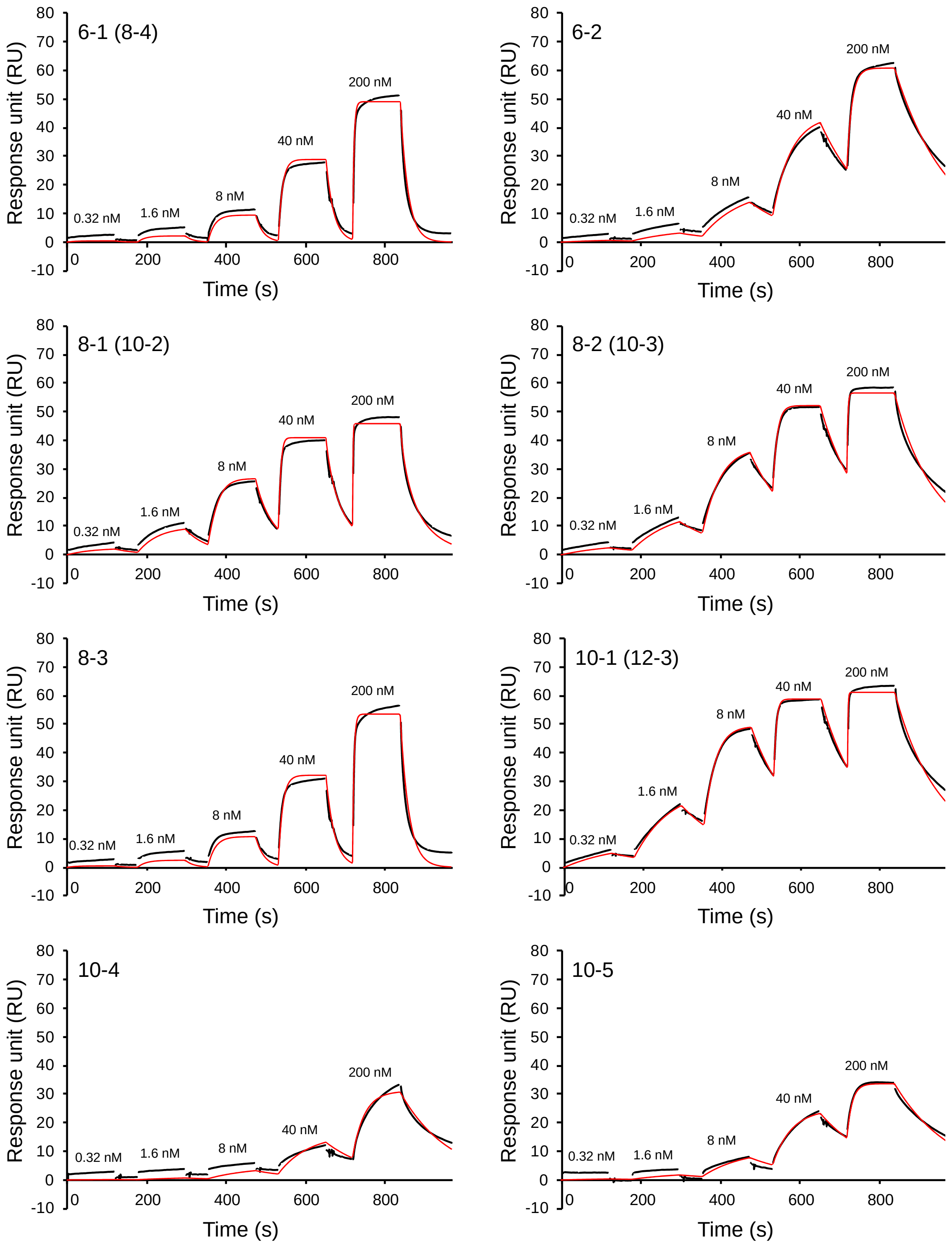

Figure S8. SPR analyses of the selected peptides binding to the MET. Experimental and fitted curves are shown in blue and black, respectively.

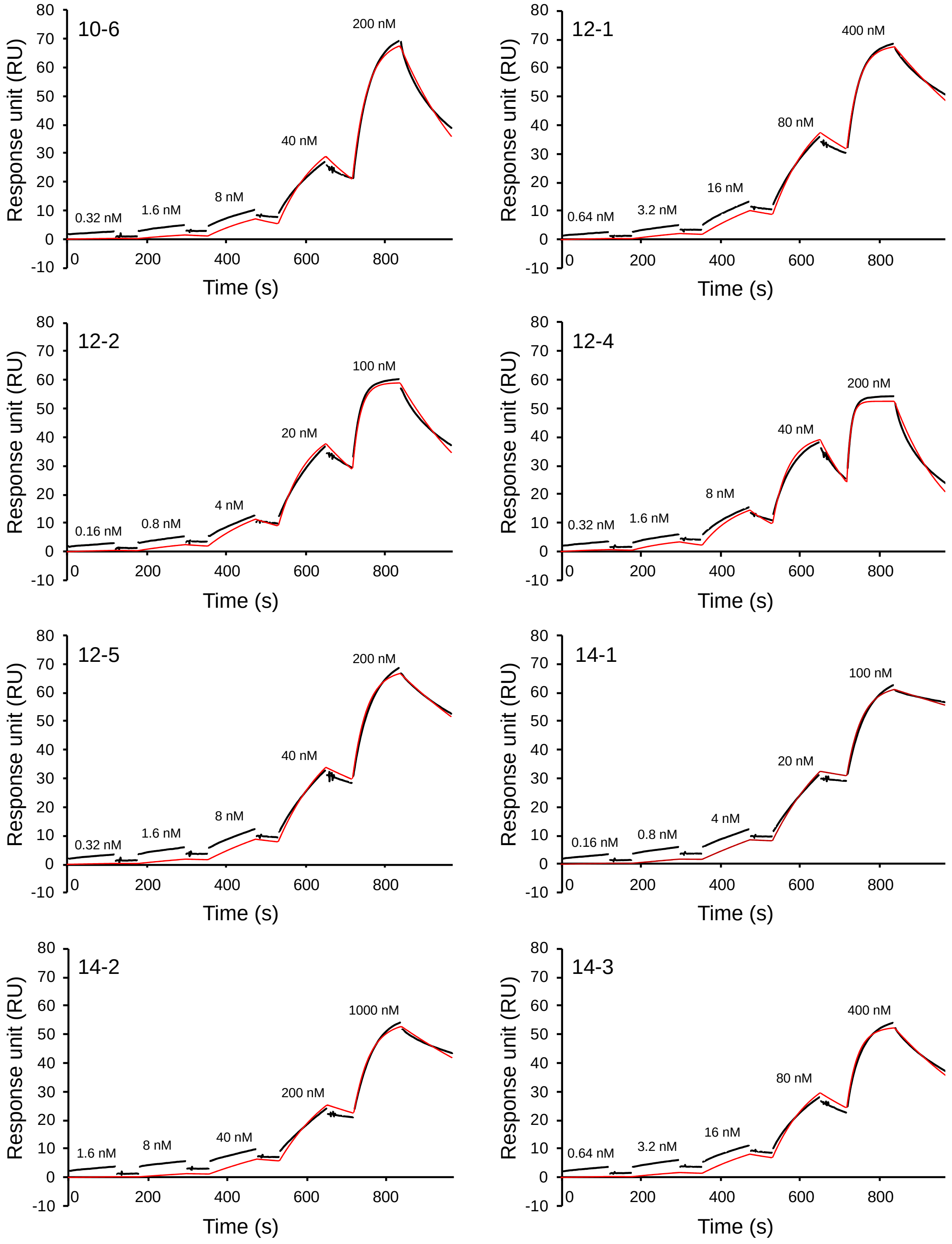

Figure S8. (Continued)

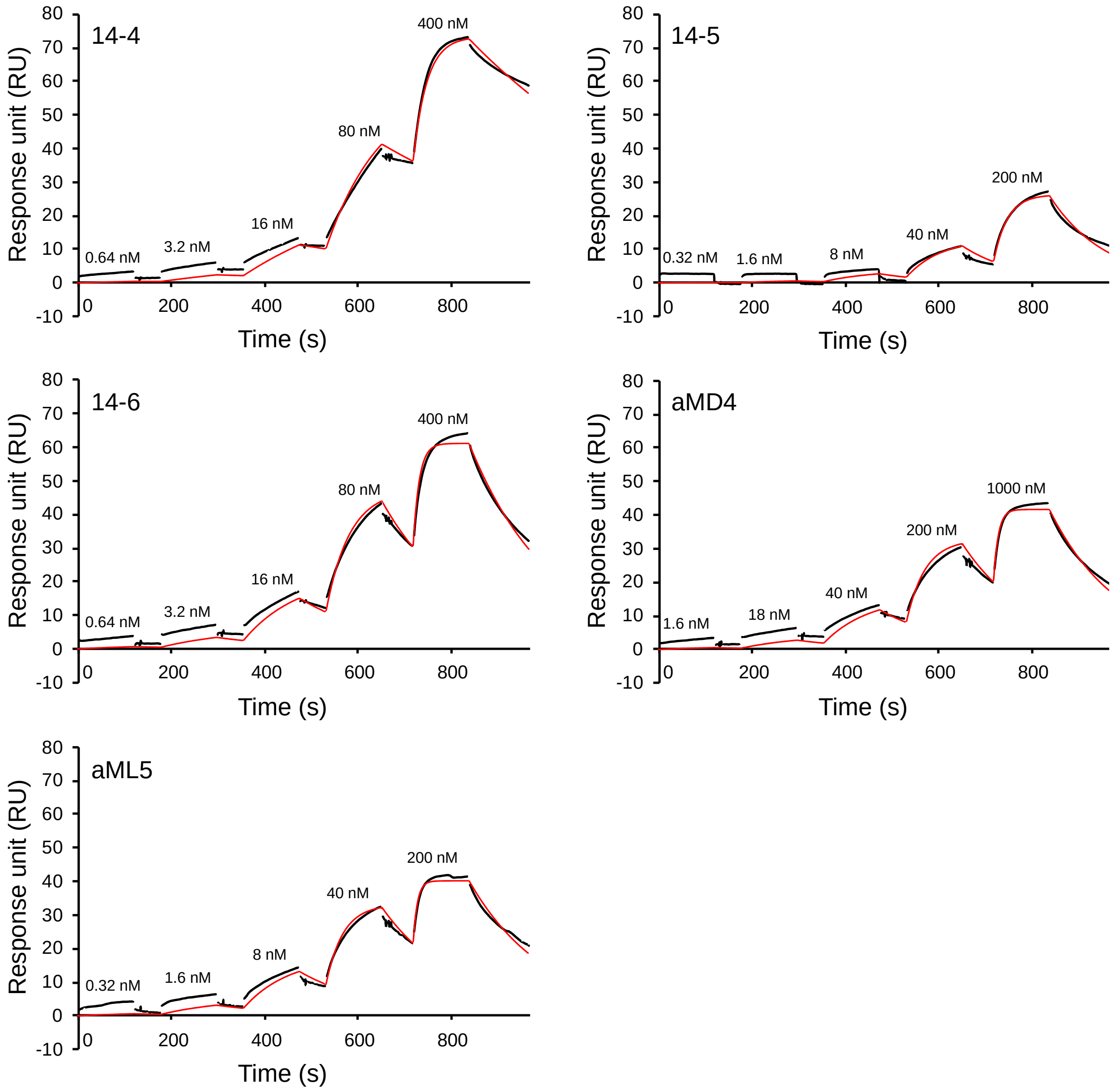

Figure S8. (Continued)

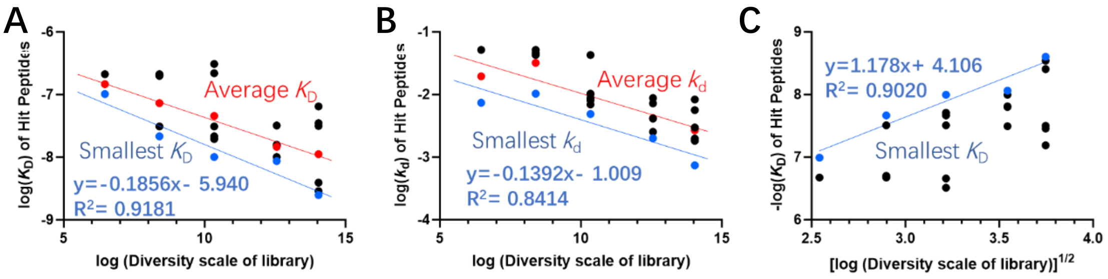

Figure S9. Correlation between K_Ds or $k$ds of hit peptides and the diversity scale of library. (A) Log(K_D) vs log(Diversity scale of library) plots, (B) Log(k_d) vs log(Diversity scale of library) plots, and (C) -Log(K_D) vs [log(Diversity scale of library)]$^{1/2}$ plots. A blue and red plot shows the smallest and the average K_D or k_d for each library, respectively.

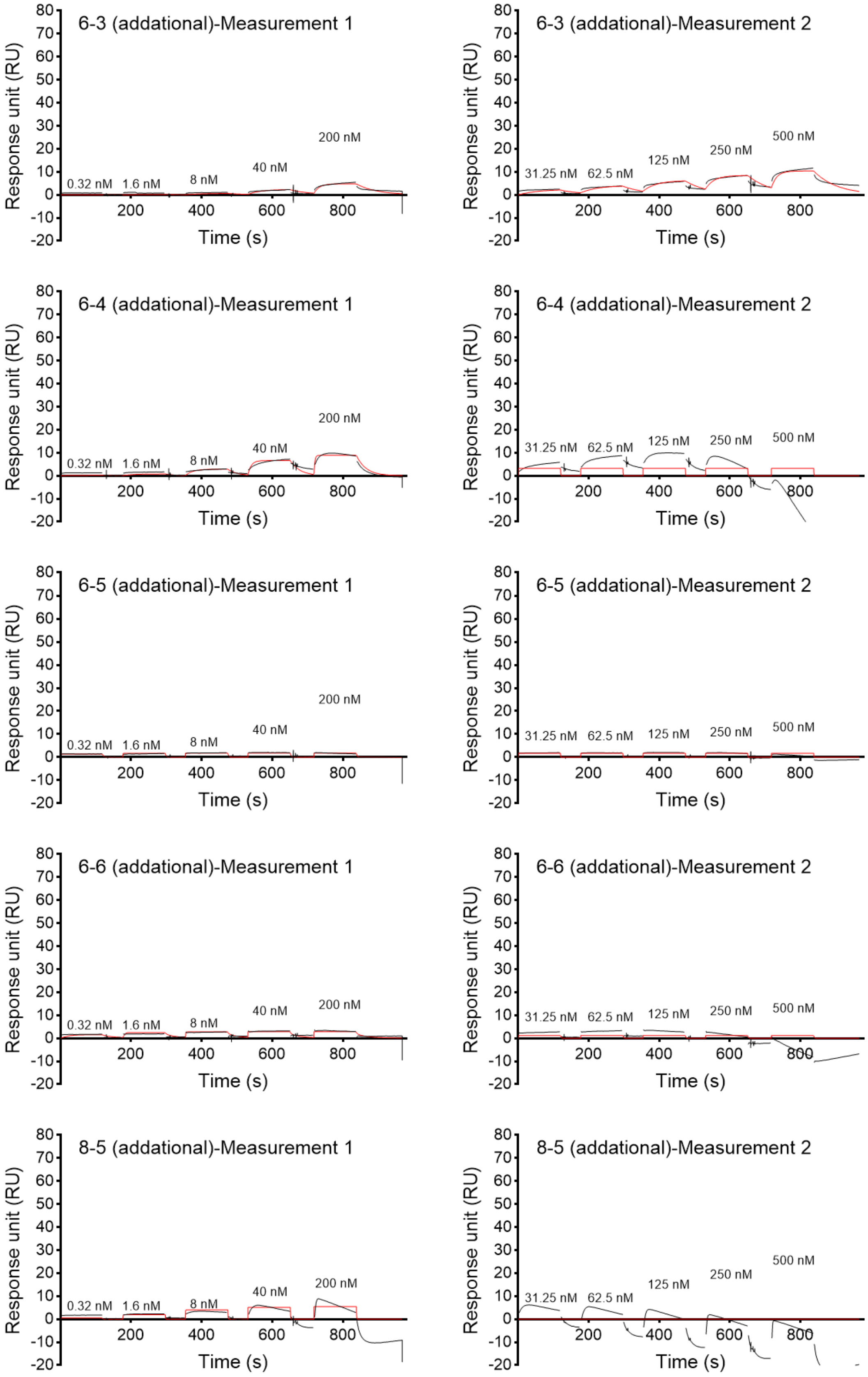
6-3 (addational)-Measurement 1
6-3 (addational)-Measurement 2
6-4 (addational)-Measurement 1
6-4 (addational)-Measurement 2
6-5 (addational)-Measurement 1
6-5 (addational)-Measurement 2
6-6 (addational)-Measurement 1
6-6 (addational)-Measurement 2
8-5 (addational)-Measurement 1
8-5 (addational)-Measurement 2
0.32 nM
1.6 nM
8 nM
40 nM
200 nM
31.25 nM
62.5 nM
125 nM
250 nM
500 nM
Response unit (RU)
Time (s)

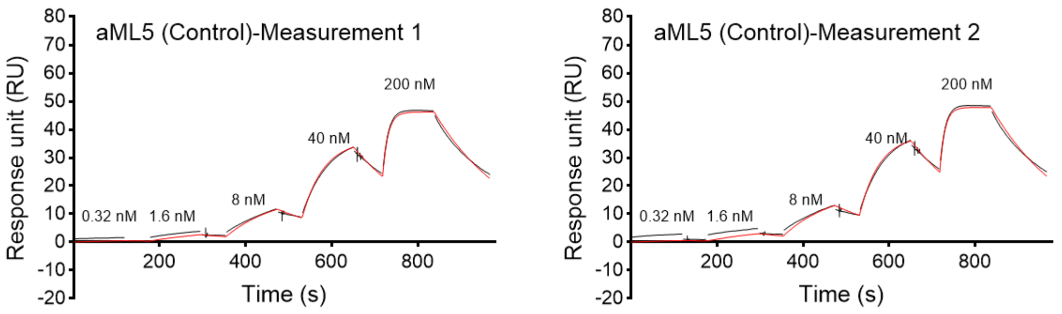

Figure S10. SPR analyses of the additional synthesized peptides binding to the MET. Two concentration ranges of peptides were tested to achieve better kinetics fitting. Peptide aML5 were also characterized as a positive control. The sequences and binding kinetic parameters of each peptide are,

6-3: cyclic-(^{Ac}YYIIYHAPLGWIKYLSC)G-NH_2, Measure 1: $k_a = 2.52 \times 10^5$ /Ms, $k_d = 1.76 \times 10^{-2}$ /s, K_D = 70.0 nM, Measure 2: : $k_a = 1.03 \times 10^5$ /Ms, $k_d = 1.51 \times 10^{-2}$ /s, K_D = 146 nM;

6-4: cyclic-(^{Ac}YRYFFVIRNRLVLTFHC)G-NH_2, Measure 1: $k_a = 1.88 \times 10^6$ /Ms, $k_d = 3.64 \times 10^{-2}$ /s; K_D = 19.4 nM, Measure 2: : $k_a = 2.31 \times 10^9$ /Ms, k_d = 1.76 /s, K_D = 0.827 nM; *These data were not used in the statistical analysis because of the low reproducibility.*

6-5: cyclic-(^{Ac}YFVVNNITGLVITLIKC)G-NH_2, Measure 1: $k_a = 4.01 \times 10^9$ /Ms, $k_d = 4.52 \times 10^{-1}$ /s, K_D = 0.113 nM, Measure 2: : $k_a = 5.28 \times 10^{11}$ /Ms, k_d = 3.79 /s, K_D = 0.00717 nM; *These data were not used in the statistical analysis because of the low signal intensity.*

6-6: cyclic-(^{Ac}YNFVLLYGTKFLSLRNC)G-NH_2, Measure 1: $k_a = 1.15\times 10^{12}$ /Ms, $k_d = 3.31\times 10^2$ /s, K_D = 0.288 nM, Measure 2: : $k_a = 9.54 \times 10^8$ /Ms, $k_d = 5.57 \times 10^{-1}$ /s, K_D = 0.583 nM; *These data were not used in the statistical analysis because of the low signal intensity.*

8-5: cyclic-(^{Ac}YWYYTFDGRWEEYGAAC)G-NH_2, Measure 1: $k_a = 5.44 \times 10^8$ /Ms, k_d = 1.57 /s, K_D = 2.89 nM, Measure 2: : $k_a = 9.65 \times 10^4$ /Ms, $k_d = 1.19 \times 10^{-3}$ /s, K_D = 12.3 nM; *These data were not used in the statistical analysis because of the negative signal.*

aML5: Measure 1: $k_a = 3.71 \times 10^5$ /Ms, $k_d = 5.54 \times 10^{-3}$ /s, K_D = 14.9 nM, Measure 2: $k_a = 4.05 \times 10^5$ /Ms, $k_d = 5.53 \times 10^{-3}$ /s, K_D = 13.7 nM.

As a result, the newly synthesized peptides, except for 6-3, were excluded from the further analysis due to poor curve fitting or lack of reproducibility at higher concentrations, indicating that they are unlikely to be active binders. The binding kinetics data from the first measurement of peptide 6-3, which showed a better curve fit than the second measurement, were included in the analysis presented in Table S5.

References

19. Hiroaki Suga, Yuki Goto, Takayuki Katoh et al. Preparation of materials for flexizyme reactions and genetic code reprogramming, 12 May 2011, PROTOCOL (Version 1) available at Protocol Exchange [https://doi.org/10.1038/protex.2011.209]
32. Yamagishi Y, Shoji I, Miyagawa S, Kawakami T, Katoh T, Goto Y, Suga H. Natural Product-Like Macrocyclic N-Methyl-Peptide Inhibitors against a Ubiquitin Ligase Uncovered from a Ribosome-Expressed De Novo Library. Chem Biol. 2011;18(12):1562-70.
33. Hayashi Y, Morimoto J, Suga H. In Vitro Selection of Anti-Akt2 Thioether-Macrocyclic Peptides Leading to Isoform-Selective Inhibitors. ACS Chem Biol. 2012;7(3):607-13.
34. Morimoto J, Hayashi Y, Suga H. Discovery of Macrocyclic Peptides Armed with a Mechanism-Based Warhead: Isoform-Selective Inhibition of Human Deacetylase SIRT2. Angew Chem-Int Edit. 2012;51(14):3423-7.
35. Kawakami T, Ishizawa T, Fujino T, Reid PC, Suga H, Murakami H. In Vitro Selection of Multiple Libraries Created by Genetic Code Reprogramming To Discover Macrocyclic Peptides That Antagonize VEGFR2 Activity in Living Cells. ACS Chem Biol. 2013;8(6):1205-14.
36. Iwasaki K, Goto Y, Katoh T, Yamashita T, Kaneko S, Suga H. A Fluorescent Imaging Probe Based on a Macrocyclic Scaffold That Binds to Cellular EpCAM. J Mol Evol. 2015;81(5-6):210-7.
37. Matsunaga Y, Bashiruddin NK, Kitago Y, Takagi J, Suga H. Allosteric Inhibition of a Semaphorin 4D Receptor Plexin B1 by a High-Affinity Macrocyclic Peptide. Cell Chem Biol. 2016;23(11):1341-50.
38. Jongkees SAK, Caner S, Tysoe C, Brayer GD, Withers SG, Suga H. Rapid Discovery of Potent and Selective Glycosidase-Inhibiting De Novo Peptides. Cell Chem Biol. 2017;24(3):381-90.
39. Yu H, Dranchak P, Li ZR, MacArthur R, Munson MS, Mehzabeen N, et al. Macrocycle peptides delineate locked-open inhibition mechanism for microorganism phosphoglycerate mutases. Nat Commun. 2017;8:13.
40. Kawamura A, Munzel M, Kojima T, Yapp C, Bhushan B, Goto Y, et al. Highly selective inhibition of histone demethylases by de novo macrocyclic peptides. Nat Commun. 2017;8:10.
41. Song X, Lu LY, Passioura T, Suga H. Macrocyclic peptide inhibitors for the protein-protein interaction of Zaire Ebola virus protein 24 and karyopherin alpha 5. Org Biomol Chem. 2017;15(24):5155-60.
42. Nishio K, Belle R, Katoh T, Kawamura A, Sengoku T, Hanada K, et al. Thioether Macrocyclic Peptides Selected against TET1 Compact Catalytic Domain Inhibit TET1 Catalytic Activity. ChemBioChem. 2018;19(9):979-85.
43. McAllister TE, Yeh TL, Abboud MI, Leung IKH, Hookway ES, King ONF, et al. Non-competitive cyclic peptides for targeting enzyme-substrate complexes. Chem Sci. 2018;9(20):4569-78.

44. Passioura T, Liu WY, Dunkelmann D, Higuchi T, Suga H. Display Selection of Exotic Macrocyclic Peptides Expressed under a Radically Reprogrammed 23 Amino Acid Genetic Code. J Am Chem Soc. 2018;140(37):11551-5.
45. Passioura T, Watashi K, Fukano K, Shimura S, Saso W, Morishita R, et al. De Novo Macrocyclic Peptide Inhibitors of Hepatitis B Virus Cellular Entry. Cell Chem Biol. 2018;25(7):906-+.
46. Nitsche C, Passioura T, Varava P, Mahawaththa MC, Leuthold MM, Klein CD, et al. De Novo Discovery of Nonstandard Macrocyclic Peptides as Noncompetitive Inhibitors of the Zika Virus NS2B-NS3 Protease. ACS Med Chem Lett. 2019;10(2):168-74.
47. Sakai K, Passioura T, Sato H, Ito K, Furuhashi H, Umitsu M, et al. Macrocyclic peptide-based inhibition and imaging of hepatocyte growth factor. Nat Chem Biol. 2019;15(6):598-606.
48. Nawatha M, Rogers JM, Bonn SM, Livneh I, Lemma B, Mali SM, et al. De novo macrocyclic peptides that specifically modulate Lys48-linked ubiquitin chains. Nat Chem. 2019;11(7):644-52.
49. Yin YZ, Ochi N, Craven TW, Baker D, Takigawa N, Suga H. De Novo Carborane-Containing Macrocyclic Peptides Targeting Human Epidermal Growth Factor Receptor. J Am Chem Soc. 2019;141(49):19193-7.
50. Huang YC, Nawatha M, Livneh I, Rogers JM, Sun H, Singh SK, et al. Affinity Maturation of Macrocyclic Peptide Modulators of Lys48-Linked Diubiquitin by a Twofold Strategy. Chem-Eur J. 2020;26(36):8022-7.
51. Otero-Ramirez ME, Matoba K, Mihara E, Passioura T, Takagi J, Suga H. Macrocyclic peptides that inhibit Wnt signalling via interaction with Wnt3a. RSC Chem Biol. 2020;1(1):26-34.
52. Johansen-Leete J, Passioura T, Foster SR, Bhusal RP, Ford DJ, Liu ML, et al. Discovery of Potent Cyclic Sulfopeptide Chemokine Inhibitors via Reprogrammed Genetic Code mRNA Display. J Am Chem Soc. 2020;142(20):9141-6.
53. Hazama D, Yin YZ, Murata Y, Matsuda M, Okamoto T, Tanaka D, et al. Macrocyclic Peptide-Mediated Blockade of the CD47-SIRP alpha Interaction as a Potential Cancer Immunotherapy. Cell Chem Biol. 2020;27(9):1181-+.
54. Katoh T, Sengoku T, Hirata K, Ogata K, Suga H. Ribosomal synthesis and de novo discovery of bioactive foldamer peptides containing cyclic beta-amino acids. Nat Chem. 2020;12(11):1081-+.
55. Katoh T, Suga H. In Vitro Selection of Foldamer-Like Macrocyclic Peptides Containing 2-Aminobenzoic Acid and 3-Aminothiophene-2-Carboxylic Acid. J Am Chem Soc. 2022;144(5):2069-72.
56. Zhang ZY, Gao R, Hu Q, Peacock H, Peacock DM, Dai SZ, et al. GTP-State-Selective Cyclic Peptide Ligands of K-Ras(G12D) Block Its Interaction with Raf. ACS Central Sci. 2020;6(10):1753-61.

57. Xie Q, Wiedmann MM, Zhao A, Pagan IR, Novick RP, Suga H, Muir TW. Discovery of quorum quenchers targeting the membrane-embedded sensor domain of theStaphylococcus aureusreceptor histidine kinase, AgrC. Chem Commun. 2020;56(76):11223-6.
58. Patel K, Walport LJ, Walshe JL, Solomon PD, Low JKK, Tran DH, et al. Cyclic peptides can engage a single binding pocket through highly divergent modes. Proc Natl Acad Sci U S A. 2020;117(43):26728-38.
59. Rogers JM, Nawatha M, Lemma B, Vamisetti GB, Livneh I, Barash U, et al. In vivo modulation of ubiquitin chains by N-methylated non-proteinogenic cyclic peptides. RSC Chem Biol. 2021;2(2):513-22.
60. Imanishi S, Katoh T, Yin YZ, Yamada M, Kawai M, Suga H. In Vitro Selection of Macrocyclic D/L-Hybrid Peptides against Human EGFR. J Am Chem Soc. 2021;143(15):5680-4.
61. Saito M, Itoh Y, Yasui F, Munakata T, Yamane D, Ozawa M, et al. Macrocyclic peptides exhibit antiviral effects against influenza virus HA and prevent pneumonia in animal models. Nat Commun. 2021;12(1):11.
62. Ford DJ, Duggan NM, Fry SE, Ripoll-Rozada J, Agten SM, Liu WY, et al. Potent Cyclic Peptide Inhibitors of FXIIa Discovered by mRNA Display with Genetic Code Reprogramming. J Med Chem. 2021;64(11):7853-76.
63. Stefan E, Obexer R, Hofmann S, Huu KV, Huang YC, Morgner N, et al. De novo macrocyclic peptides dissect energy coupling of a heterodimeric ABC transporter by multimode allosteric inhibition. eLife. 2021;10:24.
64. Haberman VA, Fleming SR, Leisner TM, Puhl AC, Feng E, Xie L, et al. Discovery and Development of Cyclic Peptide Inhibitors of CIB1. ACS Med Chem Lett. 2021;12(11):1832-9.
65. Liu WY, de Veer SJ, Huang YH, Sengoku T, Okada C, Ogata K, et al. An Ultrapotent and Selective Cyclic Peptide Inhibitor of Human beta-Factor XIIa in a Cyclotide Scaffold. J Am Chem Soc. 2021;143(44):18481-9.
66. Morgan M, Ikenoue T, Suga H, Wolberger C. Potent macrocycle inhibitors of the human SAGA deubiquitinating module. Cell Chem Biol. 2022;29(4):544-+.
67. Wakabayashi R, Kawai M, Katoh T, Suga H. In Vitro Selection of Macrocyclic alpha/beta 3-Peptides against Human EGFR. J Am Chem Soc. 2022;144(40):18504-10.
68. Dai SA, Hu Q, Gao R, Blythe EE, Touhara KK, Peacock H, et al. State-selective modulation of heterotrimeric G?s signaling with macrocyclic peptides. Cell. 2022;185(21):3950-+.
69. Vamisetti GB, Saha A, Huang YCJ, Vanjari R, Mann G, Gutbrod J, et al. Selective macrocyclic peptide modulators of Lys63-linked ubiquitin chains disrupt DNA damage repair. Nat Commun. 2022;13(1):12.
70. Vinogradov AA, Zhang Y, Hamada K, Chang JS, Okada C, Nishimura H, et al. De Novo Discovery of Thiopeptide Pseudo-natural Products Acting as

Potent and Selective TNIK Kinase Inhibitors. J Am Chem Soc. 2022:10.
71. Alteen MG, Peacock H, Meek RW, Busmann JA, Zhu S, Davies GJ, et al. Potent De Novo Macrocyclic Peptides That Inhibit O-GlcNAc Transferase through an Allosteric Mechanism. Angew Chem-Int Edit. 2022:10.
72. Ikenoue T, Oono M, So M, Yamakado H, Arata T, Takahashi R, et al. A RaPID Macrocyclic Peptide That Inhibits the Formation of alpha-Synuclein Amyloid Fibrils. ChemBioChem. 2023:8.
73. Pietz HL, Abbas A, Johnson ZL, Oldham ML, Suga H, Chen J. A macrocyclic peptide inhibitor traps MRP1 in a catalytically incompetent conformation. Proc Natl Acad Sci U S A. 2023;120(11):e2220012120.
74. Miura T, Malla TR, Owen CD, Tumber A, Brewitz L, McDonough MA, et al. In vitro selection of macrocyclic peptide inhibitors containing cyclic gamma(2,4)-amino acids targeting the SARS-CoV-2 main protease. Nat Chem. 2023;15(7):998-1005.
75. Karjalainen K, Jaalouk DE, Bueso-Ramos CE, Zurita AJ, Kuniyasu A, Eckhardt BL, et al. Targeting neuropilin-1 in human leukemia and lymphoma. Blood. 2011;117(3):920-7.
76. Zhu DL, Qin YS, Wang JJ, Zhang LW, Zou SJ, Zhu XH, Zhu L. Novel Glypican-3-Binding Peptide for in Vivo Hepatocellular Carcinoma Fluorescent Imaging. Bioconjugate Chem. 2016;27(3):831-9.
77. Chang HN, Liu BY, Qi YK, Zhou Y, Chen YP, Pan KM, et al. Blocking of the PD-1/PD-L1 Interaction by a D-Peptide Antagonist for Cancer Immunotherapy. Angew Chem-Int Edit. 2015;54(40):11760-4.
78. Bertoldo D, Khan MMG, Dessen P, Held W, Huelsken J, Heinis C. Phage Selection of Peptide Macrocycles against -Catenin To Interfere with Wnt Signaling. ChemMedChem. 2016;11(8):834-9.
79. Askoxylakis V, Marr A, Altmann A, Markert A, Mier W, Debus J, et al. Peptide-Based Targeting of the Platelet-Derived Growth Factor Receptor Beta. Molecular Imaging and Biology. 2013;15(2):212-21.
80. Cho JH, Ha NR, Koh SH, Yoon MY. Design of a PKC delta-specific small peptide as a theragnostic agent for glioblastoma. Analytical Biochemistry. 2016;496:63-70.
81. Paduano F, Ortuso F, Campiglia P, Raso C, Iaccino E, Gaspari M, et al. Isolation and Functional Characterization of Peptide Agonists of PTPRJ, a Tyrosine Phosphatase Receptor Endowed with Tumor Suppressor Activity. ACS Chem Biol. 2012;7(10):1666-76.
82. Ortuso F, Paduano F, Carotenuto A, Gomez-Monterrey I, Bilotta A, Gaudio E, et al. Discovery of PTPRJ Agonist Peptides That Effectively Inhibit in Vitro Cancer Cell Proliferation and Tube Formation. ACS Chem Biol. 2013;8(7):1497-506.
83. Qin X, Zhang C, Xue XC, Li M, Li WN, Hao Q, et al. Identification of a Novel Peptide Ligand of Human Transfrrin Receptor 1 for Targeted Tumor

Delivery Drug. Protein and Peptide Letters. 2013;20(1):96-101.
84. Mai JH, Song SX, Rui MJ, Liu D, Ding Q, Peng JL, Xu YH. A synthetic peptide mediated active targeting of cisplatin liposomes to Tie2 expressing cells. Journal of Controlled Release. 2009;139(3):174-81.
85. Ding H, Prodinger WM, Kopecek J. Identification of CD21-binding peptides with phage display and investigation of binding properties of HPMA copolymer-peptide conjugates. Bioconjugate Chem. 2006;17(2):514-23.
86. El-Mousawi M, Tchistiakova L, Yurchenko L, Pietrzynski G, Moreno M, Stanimirovic D, et al. A vascular endothelial growth factor high affinity receptor 1-specific peptide with antiangiogenic activity identified using a phage display peptide library. J Biol Chem. 2003;278(47):46681-91.
87. Naiyer MM, Saha S, Hemke V, Roy S, Singh S, Musti KV, Saha B. Identification and characterization of a human IL-10 receptor antagonist. Human Immunology. 2013;74(1):28-31.
88. Li ZH, Zhao RJ, Wu XH, Sun Y, Yao M, Li JJ, et al. Identification and characterization of a novel peptide ligand of epidermal growth factor receptor for targeted delivery of therapeutics. Faseb J. 2005;19(14):1978-85.
89. Navari M, Zare M, Javanmardi M, Asadi-Ghalehni M, Modjtahedi H, Rasaee MJ. Epitope mapping of epidermal growth factor receptor (EGFR) monoclonal antibody and induction of growth-inhibitory polyclonal antibodies by vaccination with EGFR mimotope. Immunopharmacology and Immunotoxicology. 2014;36(5):309-15.
90. Zhou J, Joshi BP, Duan XY, Pant A, Qiu Z, Kuick R, et al. EGFR Overexpressed in Colonic Neoplasia Can be Detected on Wide-Field Endoscopic Imaging. Clinical and Translational Gastroenterology. 2015;6.
91. Wang WH, Chen XL, Li T, Li YM, Wang RX, He D, et al. Screening a phage display library for a novel FGF8b-binding peptide with anti-tumor effect on prostate cancer. Exp Cell Res. 2013;319(8):1156-64.
92. Dai XY, Cai CZ, Xiao F, Xiong YL, Huang YD, Zhang QH, et al. Identification of a novel aFGF-binding peptide with anti-tumor effect on breast cancer from phage display library. Biochem Biophys Res Commun. 2014;445(4):795-801.
93. Wu XP, Yan QX, Huang YD, Huang HX, Su ZJ, Xiao J, et al. Isolation of a novel basic FGF-binding peptide with potent antiangiogenetic activity. Journal of Cellular and Molecular Medicine. 2010;14(1-2):351-6.
94. Su JL, Lai KP, Chen CA, Yang CY, Chen PS, Chang CC, et al. Novel peptide specifically binding to interleukin-beta receptor (gp80) inhibits angiogenesis and tumor growth. Cancer Res. 2005;65(11):4827-35.
95. Koivunen E, Wang BC, Ruoslahti E. ISOLATION OF A HIGHLY SPECIFIC

LIGAND FOR THE ALPHA(5)BETA(1) INTEGRIN FROM A PHAGE DISPLAY LIBRARY. J Cell Biol. 1994;124(3):373-80.
96. Murayama O, Nishida H, Sekiguchi K. Novel peptide ligands for integrin alpha 6 beta 1 selected from a phage display library. J Biochem. 1996;120(2):445-51.
97. Koivunen E, Wang BC, Ruoslahti E. PHAGE LIBRARIES DISPLAYING CYCLIC-PEPTIDES WITH DIFFERENT RING SIZES - LIGAND SPECIFICITIES OF THE RGD-DIRECTED INTEGRINS. Bio-Technology. 1995;13(3):265-70.
98. Arap W, Pasqualini R, Ruoslahti E. Cancer treatment by targeted drug delivery to tumor vasculature in a mouse model. Science. 1998;279(5349):377-80.
99. Kraft S, Diefenbach B, Mehta R, Jonczyk A, Luckenbach GA, Goodman SL. Definition of an unexpected ligand recognition motif for alpha nu beta 6 integrin. J Biol Chem. 1999;274(4):1979-85.
100. Koivunen E, Arap W, Valtanen H, Rainisalo A, Medina OP, Heikkila P, et al. Tumor targeting with a selective gelatinase inhibitor. Nature Biotechnology. 1999;17(8):768-74.
101. Devemy E, Blaschuk OW. Identification of novel N-cadherin antagonist. Peptides. 2008;29(11):1853-61.
102. Devemy E, Blaschuk OW. Identification of a novel dual E- and N-cadherin antagonist. Peptides. 2009;30(8):1539-47.
103. Aggarwal S, Singh P, Topaloglu O, Isaacs JT, Denmeade SR. A dimeric peptide that binds selectively to prostate-specific membrane antigen and inhibits its enzymatic activity. Cancer Res. 2006;66(18):9171-7.
104. Qin X, Wan Y, Li M, Xue XC, Wu SZ, Zhang C, et al. Identification of a novel peptide ligand of human vascular endothelia growth factor receptor 3 for targeted tumour diagnosis and therapy. J Biochem. 2007;142(1):79-85.
105. Shi LF, Wu Y, Li CY. Identification of high-affinity VEGFR3-binding peptides through a phage-displayed random peptide library. Journal of Gynecologic Oncology. 2015;26(4):327-35.
106. Yang L, Jiang H, Shi BZ, Wang HM, Li JJ, Wang H, et al. Identification and characterization of Ch806 mimotopes. Cancer Immunology Immunotherapy. 2010;59(10):1481-7.
107. Askoxylakis V, Garcia-Boy R, Rana S, Kramer S, Hebling U, Mier W, et al. A New Peptide Ligand for Targeting Human Carbonic Anhydrase IX, Identified through the Phage Display Technology. PLoS One. 2010;5(12).
108. Koolpe M, Dail M, Pasquale EB. An ephrin mimetic peptide that selectively targets the EphA2 receptor. J Biol Chem. 2002;277(49):46974-9.
109. Koolpe M, Burgess R, Dail M, Pasquale EB. EphB receptor-binding

peptides identified by phage display enable design of an antagonist with ephrin-like affinity. J Biol Chem. 2005;280(17):17301-11.
110. Thapa N, Kim S, So IS, Lee BH, Kwon IC, Choi K, Kim IS. Discovery of a phosphatidylserine-recognizing peptide and its utility in molecular imaging of tumour apoptosis. Journal of Cellular and Molecular Medicine. 2008;12(5A):1649-60.
111. Diderich P, Heinis C. Phage selection of bicyclic peptides binding Her2. Tetrahedron. 2014;70(42):7733-9.
112. Witsch EJ, Mahlknecht G, Wakim J, Sertchook R, Bublil E, Yarden Y, Sela M. Generation and characterization of peptide mimotopes specific for anti ErbB-2 monoclonal antibodies. International Immunology. 2011;23(6):391-403.
113. Karasseva NG, Glinsky VV, Chen NX, Komatireddy R, Quinn TP. Identification and characterization of peptides that bind human ErbB-2 selected from a bacteriophage display library. Journal of Protein Chemistry. 2002;21(4):287-96.
114. Houimel M, Schneider P, Terskikh A, Mach JP. Selection of peptides and synthesis of pentameric peptabody molecules reacting specifically with ERBB-2 receptor. International Journal of Cancer. 2001;92(5):748-55.
115. Pero SC, Shukla GS, Armstrong AL, Peterson D, Fuller SP, Godin K, et al. Identification of a small peptide that inhibits the phosphorylation of ErbB2 and proliferation of ErbB2 overexpressing breast cancer cells. International Journal of Cancer. 2004;111(6):951-60.
116. Shukla GS, Krag DN. Cancer cell-specific internalizing ligands from phage displayed beta-lactamase-peptide fusion libraries. Protein Engineering Design & Selection. 2010;23(6):431-40.
117. Shukla GS, Krag DN. Phage-displayed combinatorial peptide libraries in fusion to beta-lactamase as reporter for an accelerated clone screening: Potential uses of selected enzyme-linked affinity reagents in downstream applications. Comb Chem High Throughput Screen. 2010;13(1):75-87.
118. Rusckowski M, Gupta S, Liu GZ, Don SP, Hnatowich DJ. Evidence of speciricity of radiolabeled phage display peptides for the TAG-72 antigen. Cancer Biotherapy and Radiopharmaceuticals. 2007;22(4):564-72.
119. Chen L, Wang Y, Liu XR, Dou SP, Liu GZ, Hnatowich DJ, Rusckowski M. A new TAG-72 cancer marker peptide identified by phage display. Cancer Lett. 2008;272(1):122-32.
120. Zou J, Glinsky VV, Landon LA, Matthews L, Deutscher SL. Peptides specific to the galectin-3 carbohydrate recognition domain inhibit metastasis-associated cancer cell adhesion. Carcinogenesis.

2005;26(2):309-18.
121. Landon LA, Peletskaya EN, Glinsky VV, Karasseva N, Quinn TP, Deutscher SL. Combinatorial evolution of high-affinity peptides that bind to the Thomsen-Friedenreich carcinoma antigen. Journal of Protein Chemistry. 2003;22(2):193-204.
122. Landon LA, Zou J, Deutscher SL. Effective combinatorial strategy to increase affinity of carbohydrate binding by peptides. Molecular Diversity. 2004;8(1):35-50.
123. Peletskaya EN, Glinsky VV, Glinsky GV, Deutscher SL, Quinn TP. Characterization of peptides that bind the tumor-associated Thomsen-Friedenreich antigen selected from bacteriophage display libraries. J Mol Biol. 1997;270(3):374-84.
124. Pilch J, Brown DM, Komatsu M, Jarvinen TAH, Yang M, Peters D, et al. Peptides selected for binding to clotted plasma accumulate in tumor stroma and wounds. Proc Natl Acad Sci U S A. 2006;103(8):2800-4.
125. Ballinger MD, Shyamala V, Forrest LD, Deuter-Reinhard M, Doyle LV, Wang JX, et al. Semirational design of a potent, artificial agonist of fibroblast growth factor receptors. Nature Biotechnology. 1999;17(12):1199-204.
126. Maruta F, Parker AL, Fisher KD, Hallissey MT, Ismail T, Rowlands DC, et al. Identification of FGF receptor-binding peptides for cancer gene therapy. Cancer Gene Therapy. 2002;9(6):543-52.
127. McConnell SJ, Thon VJ, Spinella DG. Isolation of fibroblast growth factor receptor binding sequences using evolved phage display libraries. Combinatorial Chemistry & High Throughput Screening. 1999;2(3):155-63.
128. Fukuda MN, Ohyama C, Lowitz K, Matsuo O, Pasqualini R, Ruoslahti E, Fukuda M. A peptide mimic of E-selectin ligand inhibits sialyl Lewis X-dependent lung colonization of tumor cells. Cancer Res. 2000;60(2):450-6.
129. Mueller J, Gaertner FC, Blechert B, Janssen KP, Essler M. Targeting of Tumor Blood Vessels: A Phage-Displayed Tumor-Homing Peptide Specifically Binds to Matrix Metalloproteinase-2-Processed Collagen IV and Blocks Angiogenesis In vivo. Molecular Cancer Research. 2009;7(7):1078-85.
130. Wu P, Leinonen J, Koivunen E, Lankinen H, Stenman UH. Identification of novel prostate-specific antigen-binding peptides modulating its enzyme activity. European Journal of Biochemistry. 2000;267(20):6212-20.
131. Urech-Varenne C, Radtke F, Heinis C. Phage Selection of Bicyclic Peptide Ligands of the Notch1 Receptor. ChemMedChem. 2015;10(10):1754-61.
132. Zhang D, Jia H, Li W, Hou Y, Lu S, He S. Screening and

Identification of a Phage Display Derived Peptide That Specifically Binds to the CD44 Protein Region Encoded by Variable Exons. J Biomol Screen. 2016;21(1):44-53.
133. Zhang D, Jia H, Wang Y, Li WM, Hou YC, Yin SW, et al. A CD44 specific peptide developed by phage display for targeting gastric cancer. Biotechnology Letters. 2015;37(11):2311-20.
134. Wang W, Chen T, Li HC, Chen YH, Wu ZL, Feng TM, et al. Screening a novel FGF3 antagonist peptide with anti-tumor effects on breast cancer from a phage display library. Molecular Medicine Reports. 2015;12(5):7051-8.
135. Han Z, Zhou ZX, Shi XY, Wang JP, Wu XH, Sun D, et al. EDB Fibronectin Specific Peptide for Prostate Cancer Targeting. Bioconjugate Chem. 2015;26(5):830-8.
136. He XQ, Guan J, Liu F, Li J, He MR. Identification of the sAPRIL Binding Peptide and Its Growth Inhibition Effects in the Colorectal Cancer Cells. PLoS One. 2015;10(3).
137. Khemthongcharoen N, Ruangpracha A, Sarapukdee P, Rattanavarin S, Jolivot R, Jarujareet U, et al. Novel p16 binding peptide development for p16-overexpressing cancer cell detection using phage display. J Pept Sci. 2015;21(4):265-73.
138. Bose D, Nahar S, Rai MK, Ray A, Chakraborty K, Maiti S. Selective inhibition of miR-21 by phage display screened peptide. Nucleic Acids Res. 2015;43(8):4342-52.
139. Shadidi M, Sioud M. Identification of novel carrier peptides for the specific delivery of therapeutics into cancer cells. Faseb J. 2002;16(14):256-+.
140. Wang XF, Birringer M, Dong LF, Veprek P, Low P, Swettenham E, et al. A peptide conjugate of vitamin E succinate targets breast cancer cells with high ErbB2 expression. Cancer Res. 2007;67(7):3337-44.
141. Wu CH, Kuo YH, Hong RL, Wu HC. α-Enolase-binding peptide enhances drug delivery efficiency and therapeutic efficacy against colorectal cancer. Sci Transl Med. 2015;7(290):290ra91.
142. Jeong MH, Kim K, Kim EM, Cheong SJ, Lee CM, Jeong HJ, et al. In vivo and in vitro evaluation of Cy5.5 conjugated epidermal growth factor receptor binding peptide. Nuclear Medicine and Biology. 2012;39(6):805-12.
143. Hamzeh-Mivehroud M, Mahmoudpour A, Dastmalchi S. Identification of New Peptide Ligands for Epidermal Growth Factor Receptor Using Phage Display and Computationally Modeling their Mode of Binding. Chemical Biology & Drug Design. 2012;79(3):246-59.
144. Staquicini FI, Tandle A, Libutti SK, Sun J, Zigler M, Bar-Eli M, et al. A Subset of Host B Lymphocytes Controls Melanoma Metastasis

through a Melanoma Cell Adhesion Molecule/MUC18-Dependent Interaction: Evidence from Mice and Humans. Cancer Res. 2008;68(20):8419-28.
145. Wang T, D'Souza GGM, Bedi D, Fagbohun OA, Potturi LP, Papahadjopoulos-Sternberg B, et al. Enhanced binding and killing of target tumor cells by drug-loaded liposomes modified with tumor-specific phage fusion coat protein. Nanomedicine. 2010;5(4):563-74.
146. Fagbohun OA, Bedi D, Grabchenko NI, Deinnocentes PA, Bird RC, Petrenko VA. Landscape phages and their fusion proteins targeted to breast cancer cells. Protein Engineering Design & Selection. 2012;25(6):271-83.
147. Wu CX, Lo SL, Boulaire J, Hong MLW, Beh HM, Leung DSY, Wang S. A peptide-based carrier for intracellular delivery of proteins into malignant glial cells in vitro. Journal of Controlled Release. 2008;130(2):140-5.
148. Beck S, Jin X, Yin J, Kim SH, Lee NK, Oh SY, et al. Identification of a peptide that interacts with Nestin protein expressed in brain cancer stem cells. Biomaterials. 2011;32(33):8518-28.
149. Matsuo AL, Tanaka AS, Juliano MA, Rodrigues EG, Travassos LR. A novel melanoma-targeting peptide screened by phage display exhibits antitumor activity. Journal of Molecular Medicine-Jmm. 2010;88(12):1255-64.
150. Kelly K, Alencar H, Funovics M, Mahmood U, Weissleder R. Detection of invasive colon cancer using a novel, targeted, library-derived fluorescent peptide. Cancer Res. 2004;64(17):6247-51.
151. Kelly KA, Jones DA. Isolation of a colon tumor specific binding peptide using phage display selection. Neoplasia. 2003;5(5):437-44.
152. Elayadi AN, Samli KN, Prudkin L, Liu YH, Bian AH, Xie XJ, et al. A peptide selected by biopanning identifies the integrin alpha(v)beta(6) as a prognostic biomarker for nonsmall cell lung cancer. Cancer Res. 2007;67(12):5889-95.
153. Oyama T, Sykes KF, Samli KN, Minna JD, Johnston SA, Brown KC. Isolation of lung tumor specific peptides from a random peptide library: generation of diagnostic and cell-targeting reagents. Cancer Lett. 2003;202(2):219-30.
154. Nothelfer EM, Zitzmann-Kolbe S, Garcia-Boy R, Kramer S, Herold-Mende C, Altmann A, et al. Identification and Characterization of a Peptide with Affinity to Head and Neck Cancer. Journal of Nuclear Medicine. 2009;50(3):426-34.
155. Witt H, Hajdin K, Iljin K, Greiner O, Niggli FK, Schafer BW, Bernasconi M. Identification of a rhabdomyosarcoma targeting peptide by phage display with sequence similarities to the tumour lymphatic-homing peptide LyP-1. International Journal of Cancer. 2009;124(9):2026-32.

156. Pandya H, Gibo DM, Garg S, Kridel S, Debinski W. An interleukin 13 receptor alpha 2-specific peptide homes to human Glioblastoma multiforme xenografts. Neuro-Oncology. 2012;14(1):6-18.
157. Tang B, Li ZX, Huang DD, Zheng L, Li QW. Screening of a Specific Peptide Binding to VPAC1 Receptor from a Phage Display Peptide Library. PLoS One. 2013;8(1).
158. Choi JH, Lee WK, Han SH, Ha S, Ahn SM, Kang JS, et al. Identification and characterization of nonapeptide targeting a human B cell lymphoma, Raji. International Immunopharmacology. 2008;8(6):852-8.
159. Sun XL, Niu G, Yan YJ, Yang M, Chen K, Ma Y, et al. Phage Display-Derived Peptides for Osteosarcoma Imaging. Clinical Cancer Research. 2010;16(16):4268-77.
160. Wang DY, Li WB, Zhang H, Mao QW, Xia HB. A targeting peptide improves adenovirus-mediated transduction of a glioblastoma cell line. Oncology Reports. 2014;31(5):2093-8.
161. Kim Y, Lillo AM, Steiniger SCJ, Liu Y, Ballatore C, Anichini A, et al. Targeting heat shock proteins on cancer cells: Selection, characterization, and cell-penetrating properties of a peptidic GRP78 ligand. Biochemistry. 2006;45(31):9434-44.
162. Kang JQ, Zhao GH, Lin T, Tang SH, Xu GH, Hu SJ, et al. A peptide derived from phage display library exhibits anti-tumor activity by targeting GRP78 in gastric cancer multidrug resistance cells. Cancer Lett. 2013;339(2):247-59.
163. Passarella RJ, Spratt DE, van der Ende AE, Phillips JG, Wu HM, Sathiyakumar V, et al. Targeted Nanoparticles That Deliver a Sustained, Specific Release of Paclitaxel to Irradiated Tumors. Cancer Res. 2010;70(11):4550-9.
164. Essler M, Ruoslahti E. Molecular specialization of breast vasculature: A breast-homing phage-displayed peptide binds to aminopeptidase P in breast vasculature. Proc Natl Acad Sci U S A. 2002;99(4):2252-7.
165. Arap W, Kolonin MG, Trepel M, Lahdenranta J, Cardo-Vila M, Giordano RJ, et al. Steps toward mapping the human vasculature by phage display. Nat Med. 2002;8(2):121-7.
166. Joyce JA, Laakkonen P, Bernasconi M, Bergers G, Ruoslahti E, Hanahan D. Stage-specific vascular markers revealed by phage display in a mouse model of pancreatic islet tumorigenesis. Cancer Cell. 2003;4(5):393-403.
167. Fogal V, Zhang L, Krajewski S, Ruoslahti E. Mitochondrial/cell-surface protein p32/gC1qR as a molecular target in tumor cells and tumor stroma. Cancer Res. 2008;68(17):7210-8.
168. Laakkonen P, Porkka K, Hoffman JA, Ruoslahti E. A tumor-homing

peptide with a targeting specificity related to lymphatic vessels. Nat Med. 2002;8(7):751-5.
169. Hariri G, Yan HP, Wang HL, Han ZZ, Hallahan DE. Radiation-Guided Drug Delivery to Mouse Models of Lung Cancer. Clinical Cancer Research. 2010;16(20):4968-77.
170. Han Z, Fu A, Wang H, Diaz R, Geng L, Onishko H, Hallahan DE. Noninvasive assessment of cancer response to therapy. Nat Med. 2008;14(3):343-9.
171. Hallahan D, Geng L, Qu SM, Scarfone C, Giorgio T, Donnelly E, et al. Integrin-mediated targeting of drug delivery to irradiated tumor blood vessels. Cancer Cell. 2003;3(1):63-74.
172. Akita N, Maruta F, Seymour LW, Kerr DJ, Parker AL, Asai T, et al. Identification of oligopeptides binding to peritoneal tumors of gastric cancer. Cancer Sci. 2006;97(10):1075-81.
173. Sugahara KN, Teesalu T, Karmali PP, Kotamraju VR, Agemy L, Girard OM, et al. Tissue-Penetrating Delivery of Compounds and Nanoparticles into Tumors. Cancer Cell. 2009;16(6):510-20.
174. Wang JP, Liu YL, Teesalu T, Sugahara KN, Kotamrajua VR, Adams JD, et al. Selection of phage-displayed peptides on live adherent cells in microfluidic channels. Proc Natl Acad Sci U S A. 2011;108(17):6909-14.
175. Teesalu T, Sugahara KN, Kotamraju VR, Ruoslahti E. C-end rule peptides mediate neuropilin-1-dependent cell, vascular, and tissue penetration. Proc Natl Acad Sci U S A. 2009;106(38):16157-62.
176. Liu JK, Lubelski D, Schonberg DL, Wu Q, Hale JS, Flavahan WA, et al. Phage display discovery of novel molecular targets in glioblastoma-initiating cells. Cell Death and Differentiation. 2014;21(8):1325-39.
177. Mintz PJ, Cardo-Vila M, Ozawa MG, Hajitou A, Rangel R, Guzman-Rojas L, et al. An unrecognized extracellular function for an intracellular adapter protein released from the cytoplasm into the tumor microenvironment. Proc Natl Acad Sci U S A. 2009;106(7):2182-7.
178. Kelly KA, Bardeesy N, Anbazhagan R, Gurumurthy S, Berger J, Alencar H, et al. Targeted nanoparticles for imaging incipient pancreatic ductal adenocarcinoma. Plos Medicine. 2008;5(4):657-68.
179. Foight GW, Ryan JA, Gulla SV, Letai A, Keating AE. Designed BH3 Peptides with High Affinity and Specificity for Targeting Mcl-1 in Cells. ACS Chem Biol. 2014;9(9):1962-8.
180. Dutta S, Gulla S, Chen TS, Fire E, Grant RA, Keating AE. Determinants of BH3 Binding Specificity for Mcl-1 versus BcI-x(L). J Mol Biol. 2010;398(5):747-62.
181. Dutta S, Chen TS, Keating AE. Peptide ligands for pro-survival protein Bfl-1 from computationally guided library screening. ACS Chem Biol. 2013;8(4):778-88.

182. Dutta S, Ryan J, Chen TS, Kougentakis C, Letai A, Keating AE. Potent and specific peptide inhibitors of human pro-survival protein Bcl-xL. J Mol Biol. 2015;427(6 Pt B):1241-53.
183. Jenson JM, Ryan JA, Grant RA, Letai A, Keating AE. Epistatic mutations in PUMA BH3 drive an alternate binding mode to potently and selectively inhibit anti-apoptotic Bfl-1. eLife. 2017;6.
184. Rezaei Araghi R, Bird GH, Ryan JA, Jenson JM, Godes M, Pritz JR, et al. Iterative optimization yields Mcl-1-targeting stapled peptides with selective cytotoxicity to Mcl-1-dependent cancer cells. Proc Natl Acad Sci U S A. 2018;115(5):E886-E95.
185. Adams JJ, Narayanan S, Liu B, Birnbaum ME, Kruse AC, Bowerman NA, et al. T cell receptor signaling is limited by docking geometry to peptide-major histocompatibility complex. Immunity. 2011;35(5):681-93.
186. Adams JJ, Narayanan S, Birnbaum ME, Sidhu SS, Blevins SJ, Gee MH, et al. Structural interplay between germline interactions and adaptive recognition determines the bandwidth of TCR-peptide-MHC cross-reactivity. Nat Immunol. 2016;17(1):87-94.
187. Sibener LV, Fernandes RA, Kolawole EM, Carbone CB, Liu F, McAffee D, et al. Isolation of a Structural Mechanism for Uncoupling T Cell Receptor Signaling from Peptide-MHC Binding. Cell. 2018;174(3):672-87 e27.
188. Gee MH, Sibener LV, Birnbaum ME, Jude KM, Yang X, Fernandes RA, et al. Stress-testing the relationship between T cell receptor/peptide-MHC affinity and cross-reactivity using peptide velcro. Proc Natl Acad Sci U S A. 2018;115(31):E7369-e78.
189. Puthenveetil S, Liu DS, White KA, Thompson S, Ting AY. Yeast Display Evolution of a Kinetically Efficient 13-Amino Acid Substrate for Lipoic Acid Ligase. J Am Chem Soc. 2009;131(45):16430-8.
190. van Rosmalen M, Janssen BM, Hendrikse NM, van der Linden AJ, Pieters PA, Wanders D, et al. Affinity Maturation of a Cyclic Peptide Handle for Therapeutic Antibodies Using Deep Mutational Scanning. J Biol Chem. 2017;292(4):1477-89.
191. Hetrick KJ, Walker MC, van der Donk WA. Development and Application of Yeast and Phage Display of Diverse Lanthipeptides. ACS Central Sci. 2018;4(4):458-67.
192. Kimura RH, Levin AM, Cochran FV, Cochran JR. Engineered cystine knot peptides that bind alpha v beta 3, alpha v beta 5, and alpha 5 beta 1 integrins with low-nanomolar affinity. Proteins-Structure Function and Bioinformatics. 2009;77(2):359-69.
193. Kimura RH, Jones DS, Jiang L, Miao Z, Cheng Z, Cochran JR. Functional mutation of multiple solvent-exposed loops in the Ecballium elaterium trypsin inhibitor-II cystine knot miniprotein. PLoS One.

2011;6(2):e16112.
194. Silverman AP, Levin AM, Lahti JL, Cochran JR. Engineered cystine-knot peptides that bind alpha(v)beta(3) integrin with antibody-like affinities. J Mol Biol. 2009;385(4):1064-75.
195. Silverman AP, Kariolis MS, Cochran JR. Cystine-knot peptides engineered with specificities for α(IIb)β(3) or α(IIb)β(3) and α(v)β(3) integrins are potent inhibitors of platelet aggregation. J Mol Recognit. 2011;24(1):127-35.
196. Maass F, Wustehube-Lausch J, Dickgiessr S, Valldorf B, Reinwarth M, Schmoldt HU, et al. Cystine-knot peptides targeting cancer-relevant human cytotoxic T lymphocyte-associated antigen 4 (CTLA-4). J Pept Sci. 2015;21(8):651-60.
197. Glotzbach B, Reinwarth M, Weber N, Fabritz S, Tomaszowski M, Fittler H, et al. Combinatorial optimization of cystine-knot peptides towards high-affinity inhibitors of human matriptase-1. PLoS One. 2013;8(10):e76956.
198. Kruziki MA, Bhatnagar S, Woldring DR, Duong VT, Hackel BJ. A 45-Amino-Acid Scaffold Mined from the PDB for High-Affinity Ligand Engineering. Chem Biol. 2015;22(7):946-56.
199. Chan JY, Hackel BJ, Yee D. Targeting Insulin Receptor in Breast Cancer Using Small Engineered Protein Scaffolds. Mol Cancer Ther. 2017;16(7):1324-34.
200. McGee JH, Shim SY, Lee SJ, Swanson PK, Jiang SY, Durney MA, Verdine GL. Exceptionally high-affinity Ras binders that remodel its effector domain. J Biol Chem. 2018;293(9):3265-80.
201. Wang WZ, Wei ZW, Zhang D, Ma HL, Wang ZH, Bu XL, et al. Rapid Screening of Peptide Probes through In Situ Single-Bead Sequencing Microarray. Anal Chem. 2014;86(23):11854-9.
202. Aina OH, Marik J, Liu RW, Lau DH, Lam KS. Identification of novel targeting peptides for human ovarian cancer cells using "one-bead one-compound" combinatorial libraries. Mol Cancer Ther. 2005;4(5):806-13.
203. Xiao WW, Yao NH, Peng L, Liu RW, Lam KS. Near-infrared optical imaging in glioblastoma xenograft with ligand-targeting alpha 3 integrin. European Journal of Nuclear Medicine and Molecular Imaging. 2009;36(1):94-103.
204. Yao NH, Xiao WW, Wang XB, Marik J, Park SH, Takada Y, Lam KS. Discovery of Targeting Ligands for Breast Cancer Cells Using the One-Bead One-Compound Combinatorial Method. J Med Chem. 2009;52(1):126-33.
205. Xiao WW, Li TH, Bononi FC, Lac D, Kekessie IA, Liu YL, et al. Discovery and characterization of a high-affinity and high-specificity peptide ligand LXY30 for in vivo targeting of alpha 3 integrin-expressing human tumors. Ejnmmi Research. 2016;6.

206. Peng L, Liu RW, Marik J, Wang XB, Takada Y, Lam KS. Combinatorial chemistry identifies high-affinity peptidomimetics against alpha(4)beta(1) integrin for in vivo tumor imaging. Nat Chem Biol. 2006;2(7):381-9.
207. Xiao WW, Wang Y, Lau EY, Luo JT, Yao NH, Shi CY, et al. The Use of One-Bead One-Compound Combinatorial Library Technology to Discover High-Affinity alpha v beta 3 Integrin and Cancer Targeting Arginine-Glycine-Aspartic Acid Ligands with a Built-in Handle. Mol Cancer Ther. 2010;9(10):2714-23.
208. Wang Y, Xiao WW, Zhang YH, Meza L, Tseng H, Takada Y, et al. Optimization of RGD-Containing Cyclic Peptides against alpha nu beta 3 Integrin. Mol Cancer Ther. 2016;15(2):232-40.
209. Cho CF, Amadei GA, Breadner D, Luyt LG, Lewis JD. Discovery of Novel Integrin Ligands from Combinatorial Libraries Using a Multiplex "Beads on a Bead" Approach. Nano Letters. 2012;12(11):5957-65.
210. Ding H, Prodinger WM, Kopecek J. Two-step fluorescence screening of CD21-binding peptides with one-bead one-compound library and investigation of binding properties of N-(2-hydroxypropyl)methacrylamide copolymer-peptide conjugates. Biomacromolecules. 2006;7(11):3037-46.
211. Bansal S, Vu K, Liu R, Ajena Y, Xiao W, Menon SM, et al. Discovery and Characterization of a Potent Antifungal Peptide through One-Bead, One-Compound Combinatorial Library Screening. ACS Infect Dis. 2022;8(7):1291-302.
212. Kenrick SA, Daugherty PS. Bacterial display enables efficient and quantitative peptide affinity maturation. Protein Engineering Design & Selection. 2010;23(1):9-17.
213. Li WH, Lei P, Yu B, Wu S, Peng JL, Zhao XP, et al. Screening and identification of a novel target specific for hepatoma cell line HepG2 from the FliTrx bacterial peptide library. Acta Biochimica Et Biophysica Sinica. 2008;40(5):443-51.
214. Zitzmann S, Kramer S, Mier W, Mahmut M, Fleig J, Altmann A, et al. Identification of a new prostate-specific cyclic peptide with the bacterial FliTrx system. Journal of Nuclear Medicine. 2005;46(5):782-5.
215. Yang WH, Luo DF, Wang SX, Wang R, Chen R, Liu Y, et al. TMTP1, a novel tumor-homing peptide specifically targeting metastasis. Clinical Cancer Research. 2008;14(17):5494-502.
216. Dane KY, Chan LA, Rice JJ, Daugherty PS. Isolation of cell specific peptide ligands using fluorescent bacterial display libraries. Journal of Immunological Methods. 2006;309(1-2):120-9.
217. Dane KY, Gottstein C, Daugherty PS. Cell surface profiling with peptide libraries yields ligand arrays that classify breast tumor subtypes. Mol Cancer Ther. 2009;8(5):1312-8.

218. Brown CK, Modzelewski RA, Johnson CS, Wong MKK. A novel approach for the identification of unique tumor vasculature binding peptides using an E-coli peptide display library. Annals of Surgical Oncology. 2000;7(10):743-9.
219. Dong B, Wang AX, Yuan LH, Chen LS, Pu KF, Duan W, et al. Peptide-Fluorescent Bacteria Complex as Luminescent Reagents for Cancer Diagnosis. PLoS One. 2013;8(1).
