# Supplementary material for: Diversity Scale of Library Matters: Impact of mRNA Library Diversity Scales on the Discovery of Macrocyclic Peptides Targeting a Protein by the RaPID System
Source: ACS Cent Sci. 2025 Mar 10;11(3):431–40. doi: 10.1021/acscentsci.4c01021 (PMC11950852; doi:10.1021/acscentsci.4c01021)
Supplement: Supplementary file 3 — oc4c01021_si_003.pdf [file oc4c01021_si_003.pdf]

oc-2024-01021v.R1

Name: Peer Review Information for "Library sizes matter: Impact of mRNA library size on the discovery of macrocyclic peptides by the RaPID system"

First Round of Reviewer Comments

Reviewer: 1

Comments to the Author

Review of Suga et al.

In this work, Suga et al. experimentally address an important question regarding the benefits of increasing diversity in combinatorial library screening: "How do library diversity sizes affect the selection results?" By selecting a range of sample sizes from a single large mRNA display library, the authors empirically demonstrate that higher affinity clones are isolated from larger samples.

These results are of qualitative value and consistent with the intuitive notion that better clones are isolated from bigger libraries. However, the authors have an opportunity to go further by placing their results in the context of published theories. Specifically, they could demonstrate consistency with the predictions of extreme value theory, which provides a quantitative answer to the question. Two citations are particularly important:

Optimum Utilization of a Compound Collection or Chemical Library for Drug Discovery, J. Chem. Inf. Comput. Sci. 1997, 37, 5, 892–899

And

High affinity extremes in combinatorial libraries and repertoires, Journal of Theoretical Biology 261 (2009) 260–265

These works demonstrate that screening chemical or combinatorial libraries follows "extreme value theory," in which the probability distribution for extreme values (e.g., the maximum affinity in a screen) follows one of only three possible distributions: Fréchet, Gumbel, or Weibull. The logarithm of the maximum affinity is expected to be roughly proportional to the logarithm of the sample size. The data in the present manuscript was plotted in this way, with the result shown here. The authors should consider reproducing this analysis in their manuscript to generalize their findings and place them in the context of existing theory.

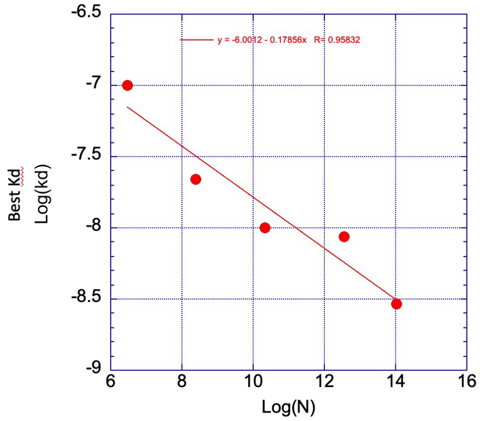

The first reference above analyzes the return on investment in screening larger libraries in an interesting way, which could be loosely applied to the present data set. This approach addresses the question: "By how much is the affinity of the best clone improved for each order of magnitude increase in library size?" Based on the data presented, the answer appears to be that a 4-log increase in library size improves the affinity of the best obtained clone by 0.5-1 log.

Reviewer: 2

Comments to the Author

This study by Hiro Suga and co-workers compares the relationship of library size and binding affinity of cyclic peptides isolated by mRNA. The team systematically prepared libraries of 5 different sizes, the largest one 1e14 members and the smallest one 1e6 members, all containing cyclic peptides with 15 random amino acids. They panned them against the target MET and characterized a larger number of isolated binders by SPR (including bidning kinetics), grouped them into consensus families, and discussed the relationship between library size and peptide affinity. As expected, there was a clear correlation, with the larger libraries yielding better binders.

This is a nice study and to my knowledge the first one that investigated so carefully and thoroughly the relationship between library size and binding affinity. The study is very detailed and while done with mRNA display, the results likely apply to any type of library, including e.g. peptide phage display libraries or also different peptide formats. While the outcome was not too surprising, I was still a bit surprised that the largest libraries (1e14 or 1e12) did not perform much better than e.g. the smallest one (1e6). Overall, I found it a bit a pity that the length of the random peptides was kept constant (always 15 random amino acids), and that the design/choice of the library was biased by

previous MET binders isolated by mRNA display, but this is a minor point. I can clearly recommend publication of this work. I encourage to consider the points below to improve it.

Suggestions for improvements:

- The first 3 paragraphs of the results section, and in particular the first one are a bit confusing, and it might be best to delete them or move to SI.

- I am not fully convinced that the largest library has truly the size of the number of ribosomes used. I recommend assessing this experimentally.

- Some peptides are highly similar (e.g. family III) and it would be interesting to see the DNA sequence, to see if they have the same codons or different ones, to learn if they mutated during the rounds of selection/amplification, or if they derive from a single clone.

Reviewer: 3

Comments to the Author

The manuscript by Hiroaki Suga and coworkers addresses a significant issue in the field of molecular discovery: they investigate the strength of binding of molecules originating from encoded libraries of varying diversity. The problem dates back to the early 90s, coinciding with the advent of high-throughput screening (HTS), DNA-encoded chemistry, and a spectrum of display technologies (phage, yeast, mRNA, bacteria, etc.). This issue has often been considered "common sense" and perpetuated by anecdotes that "screening larger libraries can find more potent binders." However, finding definitive publications that confirm or deny this statement is challenging. Thus, I applaud the Suga lab for tackling this issue, as it is crucial.

My immediate concern, however, is that the paper appears to prove a predetermined point without any counter-arguments. It is troubling that no financial disclosures were made, especially when the authors clearly stand to benefit from publishing this article (see below).

The issues begin with an "n=1" exploration of a highly complex multidimensional space. The authors used one library (n=1), composed of 20 natural amino acids, and effectively diluted it

several times to create five libraries with logarithmic diversity [6, 8, 10, 12, 14]. They selected these libraries only once (n=1) using a single target (n=1, MET hepatocyte growth factor receptor) and synthesized six or fewer molecules from each selection. With this very narrow data set, the authors draw a potentially fundamental population-wide conclusion that all kinds of peptides from all types of libraries with different diversities are (always) better if the libraries are larger. There appears to be a conscious or unconscious bias against "less diverse libraries," as the number of ligands evaluated from libraries with logarithmic diversity [6, 8, 10, 12, 14] ranges as [2, 4, 6, 5, 6] (i.e., only 2 and 4 peptides were tested from the "small, uninteresting libraries"). The publication includes some analysis of next-generation sequencing (NGS) data from each library, but it shares no raw data.

I am reluctant to recommend publishing this manuscript in its current form. I see two pathways: (1) accumulating a statistically significant and unbiased set of measurements across n>1 (see below) or (2) publishing the data as is in a different journal specializing in data disclosure from selections, with a significantly toned-down "salesmanship." The authors must caution readers that the data is biased, limited, "n-of-one-type," and should not be extrapolated to other systems (not other discovery platforms, libraries, proteins, or discovery campaigns).

Major Considerations:

1. The problem is not novel, and there must be other work in other classes of libraries (HTS, DNA-encoded, phage, antibody libraries) where this type of analysis was performed and either confirmed or debunked. It is important to review these works rather than writing a traditional, not-very-useful introduction about how "peptides are important" and "here are library sizes for OBOC and phage display."

2. "Greater diversity" does not equal "larger library." Among all people, Hiroaki Suga is in the best position to correctly formulate this question. The Suga lab can make libraries with both natural and unnatural amino acids. It can easily be argued, as Hiroaki Suga has for 20 years, that a billion-scale library made of 20 amino acids is not as diverse as a billion-scale library made of 30 amino acids. In other words, enhanced diversity of composition is a much more important argument than a mere increase in size. It is a tremendous loss of opportunity that libraries with and without UAA were not assessed.

3. While one might intuitively believe that point (2) is correct based on "common sense," this is why science is important, as evidence can trump common sense. My favorite example is from Sachdev Sidhu. Again, this is scientific evidence from the scientific literature that shows:

- Sometimes even a rudimentary diversity can find great binders
- What is true for one protein might not be true for another, and what is true for a subset of proteins is definitely, 100% NOT true for all proteins. Any generalization is very dangerous.

I think this publication, and similar publications, should be discussed in this paper. Also, this is a great example of the dangers of generalization (and authors should include this point into their publication).

4. The completely non-overlapping composition is a very inconvenient feature of the design employed by the authors. Ideally, I would expect the E6 library to be an inclusive subset of E8, the E8 library to be an inclusive subset of E10, and so on, with the E14 library containing members of E6, E8, E10, and E12. This design would allow one to determine whether the same ligand in E6, E8, and E10 is outcompeted by "better" ligands present in E12 or E14. Such a design makes it easier to test the hypotheses the authors aim to examine. Reproducibility could then serve as a powerful argument. In the current setup, the discoveries start from entirely different points, requiring reliance on a VERY large cross-sectional sample from all libraries. I simply do not believe that data extracted from a handful of measurements is representative. Long story short: the authors need more measurements of Kd.

5. Speaking of reproducibility, how consistent are these findings? If selection is re-run, does it discover the same trend? Is it possible that repeating the selection of the same E6 or E8 library for exactly the same target, but under "more optimal screening conditions," might yield better binders? There are certainly some publications out there suggesting that "knowing how to do a selection is more important than diversity."

6. It is public knowledge that Hiroaki Suga owns substantial shares of PeptiDream and is the founder and majority shareholder of MiraBiologics—both companies stand to benefit financially from claims that larger mRNA libraries called "RaPiD" are better than different or smaller libraries from competitors. PeptiDream and MiraBiologics companies will benefit financially from the advertisement of the RaPID trademark. I was surprised not to find financial disclosures in this manuscript. I keep being surprised to see that a traditional mRNA display technique, nearly indistinguishable from hundreds of other mRNA display techniques, is still referred to as "RaPID" in scientific papers. It seems like a free advertisement for an exclusively licensed platform of PeptiDream rather than a scientific demonstration of the scientific value of encoded platform technologies. Here's how other ACS publications handle it (Acct. Chem. Res.): "The authors declare the following competing financial interest(s): The FIT and RaPID systems discussed in this Account have been exclusively licensed to PeptiDream Inc. and sublicensed from PeptiDream to various pharmaceutical companies. H.S. is a shareholder of PeptiDream."

7. NGS-based exploration is potentially a saving element of this story but, I have two concerns in this regard:

7.1. NGS data should be shared as is or in some pre-processed fashion (sequences and their copy numbers). This is EXACTLY the type of publication where NGS data must be shared, allowing

both reviewers and future readers the opportunity to reassess whether the selection trajectories and NGS-derived selection pressures align with the argument that “bigger is better.”

7.2. Figure 3 is intriguing: when I look at the E6 and E14 selection NGS profile, I keep thinking: “How can one compare seven rounds of selection from a million-scale library and seven rounds from a trillion-scale library?” One of them must be not done, and the other is “overdone”, meaning undesired bias could have taken over. Again, access to NGS data is critically needed to examine these issues. We all know from work of Gerald Joyce and others (e.g., https://www.science.org/doi/10.1126/science.aag1582 DOI: 10.1126/science.aag1582) that amplification of libraries of RNA molecules is prone to the emergence of biases. As the Suga group has shown in many of their publications, repetitive selection-amplification might not be productive, and selection needs to be stopped “at the right time.” How was this knowledge taken into account when these selections were executed? Is it possible that small libraries were “unconsciously” biased and exposed to suboptimal selection conditions? How can the authors rule this out?

Author's Response to Peer Review Comments:

Hiroaki Suga, Ph.D. Professor
Bioorganic Chemistry Lab (Chemistry West Wing 2208)
Department of Chemistry, Graduate School of Science
The University of Tokyo
7-3-1 Hongo, Bunkyo-ku, Tokyo, 113-8654, JAPAN
hsuga@chem.s.u-tokyo.ac.jp
TEL&FAX: +81-3-5841-8372

ACS Central Science
Senior Editor

September 14, 2024

Manuscript ID: oc-2024-01021v
"Diversity sizes matter: Impact of mRNA library diversities on the discovery of macrocyclic peptides by the RaPID system"

Dear Editor

Thank you for the time spent on the above-referenced manuscript. Enclosed please find our revised manuscript entitled, "**Library sizes matter: Impact of mRNA library size on the discovery of macrocyclic peptides by the RaPID system**".

We have prepared our point-by-point responses to the reviewers' comments below to address their concerns, including an additional analysis. We have also revised the manuscript and formatted some sections based on the editorial instructions. The major points are below:

(1) We have added the NGS results as an additional file and an uploaded the data to the NCBI Bioproject database along with this revised manuscript (Supporting Information 2 and 3).
(2) We have revised the manuscript and changed the expressions that over-generalized the conclusion of the study in the abstract and the conclusion sections in the original manuscript.
(3) We have added a new analysis in **Figure S9**, as suggested by reviewer 1.
(4) To respond to reviewer 3, we modified the last paragraph of Introduction to clarify the question addressed in this study.
(5) The first section of Results has been simplified and modified as suggested by reviewer 2. The details of our previous work have been moved to the Supporting Information.

Although we responded to the critiques as best as we can, regarding the comments by reviewer 3, we were unable to respond to many of them scientifically because the comments are based on her/his opinions of what s/he is interested in. Many comments are not constructive, which do not criticize what we reported; i.e. critiques were given to the questions which the reviewer sets up with own reservation or opinion. Such questions were not addressed in this work. Moreover, we felt that reviewer 3 did not read the Supporting Information nor even the main text carefully. Besides, the other two reviewers of 1 and 2 recognized our work's novelty, impact, and quality of data, which contradicted to the comments or opinions by reviewer 3. If this revised manuscript is sent to the reviewer 3, I am afraid that he would not give constructive critiques or even a fair or mature review. Nevertheless, we revised the manuscript as best as possible; particularly the points shown (2) and (4) above are addressed her/his critiques.

Nevertheless, we sincerely appreciate your efforts on handling and review of our manuscript. We hope that our manuscript is now suitable for publication in *ACS Central Science*. Please feel free to contact me if you have any further questions regarding our submission.

Sincerely,

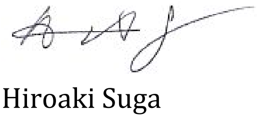

Hiroaki Suga
Professor, Department of Chemistry, Graduate School of Science
The University of Tokyo,
Tokyo, Japan

Hiroaki Suga, Ph.D. Professor
Bioorganic Chemistry Lab (Chemistry West Wing 2208)
Department of Chemistry, Graduate School of Science
The University of Tokyo
7-3-1 Hongo, Bunkyo-ku, Tokyo, 113-8654, JAPAN
hsuga@chem.s.u-tokyo.ac.jp
TEL&FAX: +81-3-5841-8372

<u>Response to Reviewer: 1</u>

Reviewer 1:

> In this work, Suga et al. experimentally address an important question regarding the benefits of increasing diversity in combinatorial library screening: "How do library diversity sizes affect the selection results?" By selecting a range of sample sizes from a single large mRNA display library, the authors empirically demonstrate that higher affinity clones are isolated from larger samples.

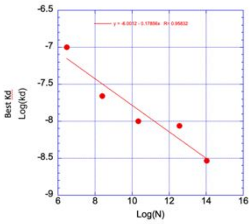

These results are of qualitative value and consistent with the intuitive notion that better clones are isolated from bigger libraries. However, the authors have an opportunity to go further by placing their results in the context of published theories. Specifically, they could demonstrate consistency with the predictions of extreme value theory, which provides a quantitative answer to the question. Two citations are particularly important:

Optimum Utilization of a Compound Collection or Chemical Library for Drug Discovery, J. Chem. Inf. Comput. Sci. 1997, 37, 5, 892–899

And

High affinity extremes in combinatorial libraries and repertoires, Journal of Theoretical Biology 261 (2009) 260–265

These works demonstrate that screening chemical or combinatorial libraries follows "extreme value theory," in which the probability distribution for extreme values (e.g., the maximum affinity in a screen) follows one of only three possible distributions: Fréchet, Gumbel, or Weibull. The logarithm of the maximum affinity is expected to be roughly proportional to the logarithm of the sample size. The data in the present manuscript was plotted in this way, with the result shown here. The authors should consider reproducing this analysis in their manuscript to generalize their findings and place them in the context of existing theory.

The first reference above analyzes the return on investment in screening larger libraries in an interesting way, which could be loosely applied to the present data set. This approach addresses the question: "By how much is the affinity of the best clone improved for each order of magnitude increase in library size?" Based on the data presented, the answer appears to be that a 4-log increase in library size improves the affinity of the best obtained clone by 0.5-1 log.

Response: We thank the reviewer for his/her insightful comments. We agree that integrating the extreme value theory into our analysis would provide a quantitative framework for our findings. We have attempted such an analysis suggested by reviewer 1, which is included in the revised manuscript on Page 19, lines 24-29, with references #20 and 21, and showed the results in **Figure S9**.

"By how much is the affinity of the best clone improved for each order of magnitude increase in library size?" This is an absolutely interesting question, but unfortunately, the higher library size more than 10^{14} cannot be examined at least by our system (or, to the best of our knowledge, no other system can do) due to the economical prohibition, i.e. experiment by increase in 10- or 100 more-fold of 10^{14} library size becomes terribly expensive. Moreover, experiments often suffer from some biases (e.g. PCR or/and contamination) when the experimental scale is too big or too small, which may skew the results that do not fit to the theory. Nevertheless, we have cited the suggested references and added a new analysis of **Figure S9** in the revised manuscript based on the reviewer's comment.

Hiroaki Suga, Ph.D. Professor
Bioorganic Chemistry Lab (Chemistry West Wing 2208)
Department of Chemistry, Graduate School of Science
The University of Tokyo
7-3-1 Hongo, Bunkyo-ku, Tokyo, 113-8654, JAPAN
hsuga@chem.s.u-tokyo.ac.jp
TEL&FAX: +81-3-5841-8372

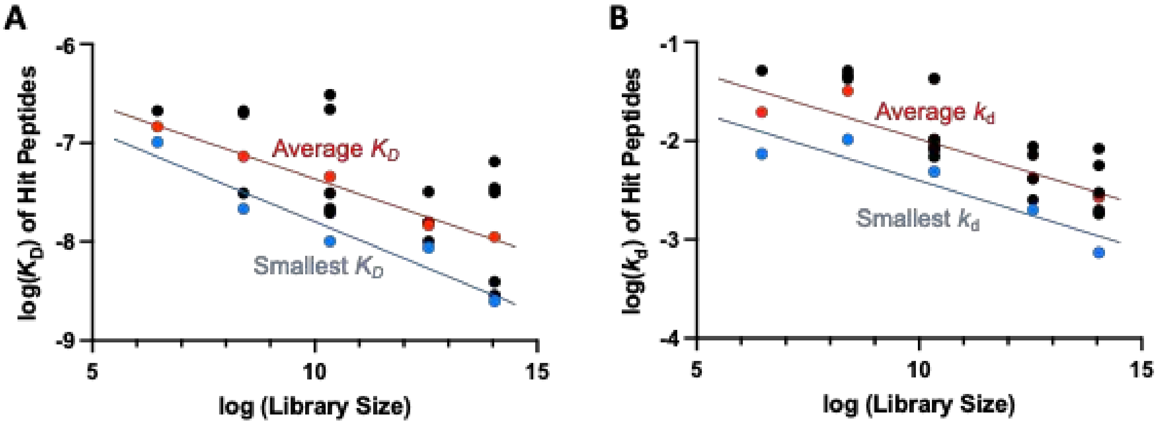

Figure S9. Correlation between K_Ds or $k$ds of hit peptides and the library size. (A) Log(K_D) vs log(Library Size) plots and (B) Log(k_d) vs log(Library Size) plots. A blue and red plot shows the smallest and the average K_D or k_d for each library, respectively.

Hiroaki Suga, Ph.D. Professor
Bioorganic Chemistry Lab (Chemistry West Wing 2208)
Department of Chemistry, Graduate School of Science
The University of Tokyo
7-3-1 Hongo, Bunkyo-ku, Tokyo, 113-8654, JAPAN
hsuga@chem.s.u-tokyo.ac.jp
TEL&FAX: +81-3-5841-8372

Response to Reviewer: 2

> This study by Hiro Suga and co-workers compares the relationship of library size and binding affinity of cyclic peptides isolated by mRNA. The team systematically prepared libraries of 5 different sizes, the largest one 1e14 members and the smallest one 1e6 members, all containing cyclic peptides with 15 random amino acids. They panned them against the target MET and characterized a larger number of isolated binders by SPR (including binding kinetics), grouped them into consensus families, and discussed the relationship between library size and peptide affinity. As expected, there was a clear correlation, with the larger libraries yielding better binders.

This is a nice study and to my knowledge the first one that investigated so carefully and thoroughly the relationship between library size and binding affinity. The study is very detailed and while done with mRNA display, the results likely apply to any type of library, including e.g. peptide phage display libraries or also different peptide formats. While the outcome was not too surprising, I was still a bit surprised that the largest libraries (1e14 or 1e12) did not perform much better than e.g. the smallest one (1e6). Overall, I found it a bit a pity that the length of the random peptides was kept constant (always 15 random amino acids), and that the design/choice of the library was biased by previous MET binders isolated by mRNA display, but this is a minor point. I can clearly recommend publication of this work. I encourage to consider the points below to improve it.

Response: We thank the reviewer for his/her insightful and constructive comments. We fixed the length to avoid a bias of selection by the variation of peptide length and facilitate the analysis. I guess that this reviewer fully understands this reason.

Suggestions for improvements:
> The first 3 paragraphs of the results section, and in particular the first one are a bit confusing, and it might be best to delete them or move to SI.

Response: Thank you for pointing out this issue; we agree with this comment. The first section of result was simplified and modified suggested by reviewer 2. The details of our previous work have moved to the Supporting Information.

> I am not fully convinced that the largest library has truly the size of the number of ribosomes used. I recommend assessing this experimentally.

Response: In the revised manuscript, we added the following sentence:

We calculated the diversity based on the assumption where ribosome does not act as polysome on the mRNA but rather act as monosome in our system due to the short length of the mRNA consisting of <100 nucleotides available for ribosome binding.[17, 18] (Page 8, lines 1-4)

It is important to note that our peptide is only a total of 17-mer macrocycle + 6 linker peptide (69 nucleotides). Its mRNA template including non-translated region and peptide-coding region together consists of <100 nt single chain region. Due to such a short length of mRNA, polysome should not be able to form, so that 1:1 complex of mRNA-ribosome should be formed. Nevertheless, the papers below have similar discussions related to this issue even though they use longer mRNA template.

17 Douthwaite, J.; Jackson, R. Ribosome Display and Related Technologies: Methods and Protocols. *Ribosome Display and Related Technologies: Methods and Protocols* **2012**, *805*, 1-424, Book.
18 Schaffitzel, C.; Hanes, J.; Jermutus, L.; Plückthun, A. Ribosome display:: an in vitro method for selection and evolution of antibodies from libraries. *Journal of Immunological Methods* **1999**,

Hiroaki Suga, Ph.D. Professor
Bioorganic Chemistry Lab (Chemistry West Wing 2208)
Department of Chemistry, Graduate School of Science
The University of Tokyo
7-3-1 Hongo, Bunkyo-ku, Tokyo, 113-8654, JAPAN
hsuga@chem.s.u-tokyo.ac.jp
TEL&FAX: +81-3-5841-8372

231 (1-2), 119-135, Article.

> Some peptides are highly similar (e.g. family III) and it would be interesting to see the DNA sequence, to see if they have the same codons or different ones, to learn if they mutated during the rounds of selection/amplification, or if they derive from a single clone.

Response: We have added the NGS data files as Supporting Information 2 and 3. According to the sequencing data, peptides having similar peptide sequences (e.g. 12-2 vs 12-3, and 8-1 vs 8-3) are actually similar in terms of their DNA sequences as shown below (top 2 DNA sequences were shown), in which some clones should not be included in the initial NNK library. We have added further explanations in the revised manuscript (Page 16, lines 17-22, and Page 17, lines 4-6).

- 12-2: YYSWSGNVSESVKWNSC(G)
  - ATG TAT AGT TGG AGT GGT AAT GTG AGT GAG TCG GTG AAG TGG AAT AGT TGT (96463 reads)
  - ATG TAT AGT TGG AGT GGT AAT GTG AGT GAG TCA GTG AAG TGG AAT AGT TGT (93 reads)
- 12-3: YYSWSGSVSESVKWNSC(G) *same peptide as 10-1*
  - ATG TAT AGT TGG AGT GGT AGT GTG AGT GAG TCG GTG AAG TGG AAT AGT TGT (37711 reads)
  - ATG TAT AGT TGG AGT GGT AGT GTG AGT GAG TCA GTG AAG TGG AAT AGT TGT (50 reads)
- 10-1: YYSWSGSVSESVKWNSC(G) *same peptide as 12-3*
  - ATG TAT AGT TGG AGT GGT AGT GTG AGT GAG TCG GTG AAG TGG AAT AGT TGT (161041 reads)
  - ATG TAT AGC TGG AGT GGT AGT GTG AGT GAG TCG GTG AAG TGG AAT AGT TGT (447 reads)

- 8-1: YWYYTFDGRWKEYGAAC(G) *same peptide as 10-2*
  - ATG TGG TAT TAT ACG TTT GAT GGG CGT TGG AAG GAG TAT GGT GCG GCT TGT (44421 reads)
  - ATG TGG TAT TAT ACG TTT GAT GGG CGT TGG AAG GAG TAT GGA GCG GCT TGT (92 reads)
- 8-3: YWYYTFDGRWEEYDAAC(G)
  - ATG TGG TAT TAT ACG TTT GAT GGG CGT TGG GAG GAG TAT GAT GCG GCT TGT (5540 reads)
  - ATG TGG TAT TAT ACG TTT GAT GGG CGT TGG GAG GAG TAT GAC GCG GCT TGT (9 reads)
- 10-2: YWYYTFDGRWKEYGAAC(G) *same peptide as 8-1*
  - ATG TGG TAT TAT ACG TTT GAT GGG CGT TGG AAG GAG TAT GGT GCG GCT TGT (14038 reads)
  - ATG TGG TAT TAT ACG TTT GAT GGG CGT TGG AAG GAG TAT GGT GCA GCT TGT (29 reads)

Reviewer link of NGS data (Supporting Information 3):
https://apac01.safelinks.protection.outlook.com/?url=https%3A%2F%2Fdataview.ncbi.nlm.nih.gov%2Fobject%2FPRJNA1151868%3Freviewer%3D9kfgpvq01qdvsojntfu8esjq81&data=05%7C02%7C%7C14772690d2ca4773107f08dcc5bdea90%7C84df9e7fe9f640afb435aaaaaaaaaaaa%7C1%7C0%7C638602666681286189%7CUnknown%7CTWFpbGZsb3d8eyJWIjoiMC4wLjAwMDAiLCJQIjoiV2luMzIiLCJBTiI6Ik1haWwiLCJXVCI6Mn0%3D%7C0%7C%7C%7C&sdata=gTU7uO0lrnKqEzJvIW4qbKsWg1hevcPkH4KGaRB0ITw%3D&reserved=0

Hiroaki Suga, Ph.D. Professor
Bioorganic Chemistry Lab (Chemistry West Wing 2208)
Department of Chemistry, Graduate School of Science
The University of Tokyo
7-3-1 Hongo, Bunkyo-ku, Tokyo, 113-8654, JAPAN
hsuga@chem.s.u-tokyo.ac.jp
TEL&FAX: +81-3-5841-8372

Responses to Reviewer: 3

Reviewer 3:
> The manuscript by Hiroaki Suga and coworkers addresses a significant issue in the field of molecular discovery: they investigate the strength of binding of molecules originating from encoded libraries of varying diversity. The problem dates back to the early 90s, coinciding with the advent of high-throughput screening (HTS), DNA-encoded chemistry, and a spectrum of display technologies (phage, yeast, mRNA, bacteria, etc.). This issue has often been considered "common sense" and perpetuated by anecdotes that "screening larger libraries can find more potent binders." However, finding definitive publications that confirm or deny this statement is challenging. Thus, I applaud the Suga lab for tackling this issue, as it is crucial.

> My immediate concern, however, is that the paper appears to prove a predetermined point without any counter-arguments. It is troubling that no financial disclosures were made, especially when the authors clearly stand to benefit from publishing this article (see below).

Response: We thank this argumental comment. Please see the responses to the individual comments below.

Major Considerations:
> Comment 1: The problem is not novel, and there must be other work in other classes of libraries (HTS, DNA-encoded, phage, antibody libraries) where this type of analysis was performed and either confirmed or debunked. It is important to review these works rather than writing a traditional, not-very-useful introduction about how "peptides are important" and "here are library sizes for OBOC and phage display."

Response 1: Even though this reviewer criticizes the novelty of our findings, to the best of our knowledge, there is no report experimentally showing systematic validations of the outcomes from different diversity of libraries in the range of 10^6–10^{14}, including OBOC, DEL, phage and mRNA displays. We appreciate if the reviewer knew such reports, please point the report(s) and explain what exact reason s/he thinks lacking the novelty of our work.

In fact, the reviewer 2 gave a comment, “This is a nice study and to my knowledge the first one that investigated so carefully and thoroughly the relationship between library size and binding affinity”. To the best of our knowledge, this work represents for the first time that a mRNA-display selection of binders against a target using libraries with the diversity from low (10^6) to as high as 10^{14} (or even 10^{12}) were conducted and the data were thoroughly analyzed. Other technologies, such as phage display or DEL, cannot deal with such high diverse libraries, and therefore no report should exist in the literature for the level of library diversities discussed in this study. Most importantly, the systematic study reported in this manuscript also revealed the correlation between the properties of hits and the library size. This reviewer should recognize that (1) no report available for reproducing the selection with smaller sizes of the library (because it will be likely unsuccessful) or (2) a smart scientist like this reviewer <u>intuitively (not empirically) believes</u> that a larger library always could provide better results. In this work, we did validate experimentally such a notion, showing the experimental evidence. For this reason, we consider that this work is undoubtedly novel.

> comment 2: "Greater diversity" does not equal "larger library." Among all people, Hiroaki Suga is in the best position to correctly formulate this question. The Suga lab can make libraries with both natural and unnatural amino acids. It can easily be argued, as Hiroaki Suga has for 20 years,

Hiroaki Suga, Ph.D. Professor
Bioorganic Chemistry Lab (Chemistry West Wing 2208)
Department of Chemistry, Graduate School of Science
The University of Tokyo
7-3-1 Hongo, Bunkyo-ku, Tokyo, 113-8654, JAPAN
hsuga@chem.s.u-tokyo.ac.jp
TEL&FAX: +81-3-5841-8372

that a billion-scale library made of 20 amino acids is not as diverse as a billion-scale library made of 30 amino acids. In other words, enhanced diversity of composition is a much more important argument than a mere increase in size. It is a tremendous loss of opportunity that libraries with and without UAA were not assessed.

Response 2: First of all, we don't understand why this reviewer made the comment of "Greater diversity" does not equal "larger library." We did not discuss "Greater diversity" equals to "larger library" in this manuscript at all. Thus, this comment is nothing to do with our work, or at least the aims we set do not relate to this comment.

His/her comment of "a billion-scale library made of 20 amino acids is not as diverse as a billion-scale library made of 30 amino acids" is incorrect. The experimental diversity size of this level of length should be determined by the experimental scale, at least mRNA display system, since the diversity size of all combination of more than 20 amino acids exceeds more than 10^{14}. Regarding the comment of "In other words, enhanced diversity of composition is a much more important argument than a mere increase in size", to the best of our knowledge there is no experimental evidence to claim this comment.

In any case, we disagree with the reviewer's comment that a library incorporating unnatural amino acids (UAAs) has more chance to give better (or more potent) binders. I assume that this is this reviewer's opinion or expectation. However, to the best of our knowledge, there is no evidence saying such. We do work on peptides containing UAAs as this reviewer recognizes, and we can say that the incorporation of UAAs in the peptide chain could contribute to improving protease resistance and cell membrane permeability. However, the fact is that even without UAA, we are still able to obtain potent (single digit nM K_Ds) binders against various targets, including MET as demonstrated in this work. This work was not designed to aim at proving if the incorporation of UAAs can generate more potent binders. Therefore, we think that this comment is out of the scope of the current work even though we are able to work on UAAs-incorporated peptide selections. We are indeed interested in exploring such investigations in the future, and this reviewer needs to wait for our next findings.

> comment 3: While one might intuitively believe that point (2) is correct based on "common sense," this is why science is important, as evidence can trump common sense. My favorite example is from Sachdev Sidhu. Again, this is scientific evidence from the scientific literature that shows:
• Sometimes even a rudimentary diversity can find great binders
• What is true for one protein might not be true for another, and what is true for a subset of proteins is definitely, 100% NOT true for all proteins. Any generalization is very dangerous.
I think this publication, and similar publications, should be discussed in this paper. Also, this is a great example of the dangers of generalization (and authors should include this point into their publication).

Response 3: We never state that our data reported in this work allows to generalize all selection outcomes using all selection platform (see the title of this work). In introduction in the original manuscript, we stated as follows:

"Despite the apparent advantage of library diversity in discovering potent peptides, the selection outcome is not solely dependent on the scale of library diversity, still shaped by various factors such as library design, amino acid selection, reproducibility of methods, and the expertise of the researchers."

This statement says that the selection outcomes largely rely on such factors. Common sense, which this reviewer said, is "intuitively believe". This is why we performed this series of experiment with

Hiroaki Suga, Ph.D. Professor
Bioorganic Chemistry Lab (Chemistry West Wing 2208)
Department of Chemistry, Graduate School of Science
The University of Tokyo
7-3-1 Hongo, Bunkyo-ku, Tokyo, 113-8654, JAPAN
hsuga@chem.s.u-tokyo.ac.jp
TEL&FAX: +81-3-5841-8372

consideration that there are many factors influencing the selection outcomes. In our work, we minimize such potential variation factors and test more specific question if the diversity sizes matter for the selection outcomes. The fact is that there would not be an experimentally supported her/his "common sense". This "assumption or believe" might be that higher diverse libraries should give "better results". However, to the best of our knowledge, there is no systematic study and no experimental analysis of selection outcomes using 10^6–10^{14} diverse libraries.

> comment 4: The completely non-overlapping composition is a very inconvenient feature of the design employed by the authors. Ideally, I would expect the E6 library to be an inclusive subset of E8, the E8 library to be an inclusive subset of E10, and so on, with the E14 library containing members of E6, E8, E10, and E12. This design would allow one to determine whether the same ligand in E6, E8, and E10 is outcompeted by "better" ligands present in E12 or E14. Such a design makes it easier to test the hypotheses the authors aim to examine. Reproducibility could then serve as a powerful argument. In the current setup, the discoveries start from entirely different points, requiring reliance on a VERY large cross-sectional sample from all libraries. I simply do not believe that data extracted from a handful of measurements is representative. Long story short: the authors need more measurements of Kd.

Response 4: Again, we consider that this criticism is based on her/his opinion or intuitive belief rather than analysis of our experimental data without reservation. Our experiments were designed to investigate following two things under the limited resource; (1) to see how binders were competed out by better binders using "overlap libraries" (E14, E10, E8) as the reviewer suggested, and (2) to see a reproducibility using "non-overlap libraries" (Group 1 and 2). The comment by this reviewer "This design would allow one to determine whether the same ligand in E6, E8, and E10 is outcompeted by "better" ligands present in E12 or E14. Such a design makes it easier to test the hypotheses the authors aim to examine. Reproducibility could then serve as a powerful argument" is correct as we reported in this work. Importantly, this precious "experimental" example allows for readers to consider how their selection system or conditions can be set and improved.

In worse, the reviewer "believes" that data extracted from a handful of measurements are not representative, but on the other hand there is no reason to deny our outcomes as experimental evidence. Regarding the reproducibility, we had previously reported the selection against MET using a different library by a different person, and thus this work is a kind of reproduction of the previous selection. Note that when we performed the previous selection against MET, we did not have the capability of deep sequencing so that only a few sequences picked by cloning could be tested for activity. The fact is that we did find the family of F-II to which the previously discovered aML5 belongs, and thus discovery of the important peptide family was reproduced. On the other hand, we also found new families and independent species with a wider range of K_Ds by means of NGS since the library is different from the previous work. Therefore, we were able to shed light on the importance of having high diverse libraries as well as low diverse libraries where weak affinity species were completely lost during the selection using only high diverse libraries. Thus, this reviewer should recognize our outcomes without own opiniated reservation.

> comment 5: Speaking of reproducibility, how consistent are these findings? If selection is re-run, does it discover the same trend? Is it possible that repeating the selection of the same E6 or E8 library for exactly the same target, but under "more optimal screening conditions," might yield better binders? There are certainly some publications out there suggesting that "knowing how to do a selection is more important than diversity."

Response 5: First of all, the last sentence is her/his opinion rather than criticism to our work. Again,

Hiroaki Suga, Ph.D. Professor
Bioorganic Chemistry Lab (Chemistry West Wing 2208)
Department of Chemistry, Graduate School of Science
The University of Tokyo
7-3-1 Hongo, Bunkyo-ku, Tokyo, 113-8654, JAPAN
hsuga@chem.s.u-tokyo.ac.jp
TEL&FAX: +81-3-5841-8372

as stated in the responses 3 and 4, we stated the selection outcomes rely on many factors but yet reproduced to an extent. Moreover, our questions were directed to:

"Do diversity sizes really matter?" and "How do the library diversity sizes affect the selection results?"

We are not asking the question(s) what this reviewer asked in our experiments. We cannot accept criticisms which we did not attempt to achieve in this work. It seems that this reviewer gives us criticisms aiming at postponing or prohibiting the publication by unnecessary or nearly infeasible additional experiments.

> comment 6: It is public knowledge that Hiroaki Suga owns substantial shares of PeptiDream and is the founder and majority shareholder of MiraBiologics—both companies stand to benefit financially from claims that larger mRNA libraries called "RaPiD" are better than different or smaller libraries from competitors. PeptiDream and MiraBiologics companies will benefit financially from the advertisement of the RaPID trademark. I was surprised not to find financial disclosures in this manuscript. I keep being surprised to see that a traditional mRNA display technique, nearly indistinguishable from hundreds of other mRNA display techniques, is still referred to as "RaPID" in scientific papers. It seems like a free advertisement for an exclusively licensed platform of PeptiDream rather than a scientific demonstration of the scientific value of encoded platform technologies. Here's how other ACS publications handle it (Acct. Chem. Res.): "The authors declare the following competing financial interest(s): The FIT and RaPID systems discussed in this Account have been exclusively licensed to PeptiDream Inc. and sublicensed from PeptiDream to various pharmaceutical companies. H.S. is a shareholder of PeptiDream."

Response 6: Even though the corresponding author funded the companies (by the way, he is no longer affiliating to PeptiDream), this work was neither patented nor supported by a budget provided by neither company. This work occurred by a pure academic interest and supported by a public grant. Therefore, there is no competing financial interest. Even though the systems have been licensed, this work does not lead any financial benefits to neither company. This reviewer should recognize what "competing financial interest" means.

> comment 7: NGS-based exploration is potentially a saving element of this story but, I have two concerns in this regard:
Comment 7-1: NGS data should be shared as is or in some pre-processed fashion (sequences and their copy numbers). This is EXACTLY the type of publication where NGS data must be shared, allowing both reviewers and future readers the opportunity to reassess whether the selection trajectories and NGS-derived selection pressures align with the argument that "bigger is better."

Response 7-1: First of all, our conclusion is not "bigger is better." As stated in the title, "Library sizes matter" is the take-home message of this work. This reviewer should read our paper more carefully without own opiniated reservation.

Nevertheless, we have uploaded NGS result of the initial library and enriched library of every selection round.

Comment 7-2: Figure 3 is intriguing: when I look at the E6 and E14 selection NGS profile, I keep thinking: "How can one compare seven rounds of selection from a million-scale library and seven rounds from a trillion-scale library?" One of them must be not done, and the other is "overdone", meaning undesired bias could have taken over. Again, access to NGS data is critically needed to examine these issues. We all know from work of Gerald Joyce and others

Hiroaki Suga, Ph.D. Professor
Bioorganic Chemistry Lab (Chemistry West Wing 2208)
Department of Chemistry, Graduate School of Science
The University of Tokyo
7-3-1 Hongo, Bunkyo-ku, Tokyo, 113-8654, JAPAN
hsuga@chem.s.u-tokyo.ac.jp
TEL&FAX: +81-3-5841-8372

(e.g., https://www.science.org/doi/10.1126/science.aag1582 DOI: 10.1126/science.aag1582) that amplification of libraries of RNA molecules is prone to the emergence of biases. As the Suga group has shown in many of their publications, repetitive selection-amplification might not be productive, and selection needs to be stopped "at the right time." How was this knowledge taken into account when these selections were executed? Is it possible that small libraries were "unconsciously" biased and exposed to suboptimal selection conditions? How can the authors rule this out?

Response 7-2: First of all, thank you for these well thought comments. We appreciate her/his knowledge in this matter. However, to address these questions, a completely different set of experiments must be designed and performed. Regardless the issue if the selection was stopped at the right time or not, our NGS analyses have covered the progress (or evolution) of sequences appeared in each round of selection. Therefore, we do not need to judge if the selection needs to be stopped "at the right time" or not. Please read our Supporting Information more carefully.

Regarding the questions of "Is it possible that small libraries were "unconsciously" biased and exposed to suboptimal selection conditions?", and "How can the authors rule this out?", unfortunately we do not understand why such questions were raised by this reviewer. We simply built the libraries of 10^6–10^{10} diversities by the dilution of mother libraries. We therefore cannot control such a bias regardless of conscious nor unconscious manner at all. This reviewer should read our experimental method more carefully or even the main text describing the method.

Reviewer link of NGS data (Supporting Information 3):
https://apac01.safelinks.protection.outlook.com/?url=https%3A%2F%2Fdataview.ncbi.nlm.nih.gov%2Fobject%2FPRJNA1151868%3Freviewer%3D9kfgpvq01qdvsojntfu8esjq81&data=05%7C02%7C%7C14772690d2ca4773107f08dcc5bdea90%7C84df9e7fe9f640afb435aaaaaaaaaaaa%7C1%7C0%7C638602666681286189%7CUnknown%7CTWFpbGZsb3d8eyJWIjoiMC4wLjAwMDAiLCJQIjoiV2luMzIiLCJBTiI6Ik1haWwiLCJXVCI6Mn0%3D%7C0%7C%7C%7C&sdata=gTU7uO0lrnKqEzJvIW4qbKsWg1hevcPkH4KGaRB0ITw%3D&reserved=0

oc-2024-01021v.R2

Name: Peer Review Information for "Diversity scale of library matters: Impact of mRNA library diversity scales on the discovery of macrocyclic peptides targeting a protein by the RaPID system"

Second Round of Reviewer Comments

Reviewer: 2

Comments to the Author

The authors have followed all suggestion to improve the manuscript. Most important, they now provide the NGS data. I can recommend publication.

Reviewer: 3

Comments to the Author

To recap the items raised in my first review:

1. The authors must cite prior work that looked at the problem that they are looking. 2. The authors should acknowledge the difference between "size" and "diversity"
3. The authors must not generalize their local n-of-1 case.
4. The authors need to discuss the reproducibility and statistical power of their findings
5. The authors need to acknowledge that their n-of-1 selection under uniform conditions is not optimal for each library.
6. Conflict of interest must exist in this manuscript.
7. NGS data must be shared this manuscript
 - 7.2: NGS data may confirm (and it indeed confirmed) my concerns from Question 5.

My major concern was (and remains) in Question 4. Remedy to these concerns exist in **Questions 5** and **Question 7.2**.
I suggested testing of additional number of peptides emanating from smaller libraries (E6, E8) because the study is biased towards testing of fewer outcomes from selection from lower diversity libraries (E6: 2 hits, E8: 4 hits; E10: 6 hits; E12: 5 hits; E14: 6 hits). Concurrently, such study concludes that lower diversity libraries deliver less potent hits. The authors need to validate the statistical power of their conclusion and reject the "null hypothesis". Below I prove this "null

hypothesis" that screening of low diversity libraries and higher diversity libraries produce the same distribution of affinity and I prove it with 6-30% confidence.

I am simply asking for a standard "pre-submission checklist" analogous to checklists that are mandatory for submission of any publications to Nature Publishing Group or Cell Publishing Group. Questions on this check lists are: "have you calculated statistical significance of your findings / have you proven the normality of distributions if your significance tests assume normality / etc.". While this is not a mandate for submission to ACS publications, I see no reason to omit testing of statistical significance of findings from this **scientific publication**. Scientific hypothesis needs to uphold statistical test. I recommended for analysis is performed by the authors.

To avoid any doubt, I ran the basic statistical analysis of the paper on my own and arrived at a preliminary conclusion that differences between any two selection strategies are **not distinguishable from a "null hypothesis"** based on pairwise non-parametric Wilcoxon test ("ranksum" function in MatLab https://www.mathworks.com/help/stats/ranksum.html).

To start: I summarized all Kd values of all measured peptides from screening of E6-E14 libraries

**Current Sample**
E6: (212, 102)
E8: (21, 31, 199, 212)
E10: (10, 19, 21, 31, 219, 308)
E12: (8, 10, 15, 16, 32);
E14: (2, 3, 4, 31, 35, 64)

The manuscript builds on an observation that there exist a tendency (of ***some*** statistical significance) implying that numbers in E14 and maybe E12 sample are significantly lower than numbers in E6 and/or E8 samples. An alternative hypothesis is that any tendency observed in E6-E8-E12-E14 series is merely by chance. Given current sample size, one must **reject** the "null hypothesis" that the mean value of any pair (e.g., E6 and E14) is identical; they come from the same distribution (i.e., they represent the same observation); the observed differences between E6 and E14 distributions are merely a chance. If they are not, then E6 and E14 originate from the same distribution and they differ merely by chance (**and hence, the remainder of the story is irrelevant**).

To avoid any doubt, I ran each pairwise test for all E combinations and summarized the p-values below.

	E6	E8	E10	E12	E14

E6		0.6667	0.6429	0.0952	0.0714
E8			0.7143	0.0635	0.1810
E10				0.1385	0.3312
E12					0.9307
E14					

Values above the diagonal are calculated by P = ranksum(X,Y) MatLab function that performs a twosided rank sum test (Wilcoxon test) of the hypothesis that two independent samples, in the vectors X and Y, come from distributions with equal medians, and returns the p-value from the test. P is the probability of observing the given result, or one more extreme, by chance if the null hypothesis ("medians are equal") is true. The two sets of data are assumed to come **from any continuous distributions** that are identical except possibly for a location shift, but are otherwise arbitrary.

In other words: all p-values for all pairs is greater than 5%, and even "intuitively correct assumption" that E6 and E14 populations are different has 7.14% chance that the difference is due to random sampling.

For illustration, I then ran another, incorrect test. Specifically Student ttest commonly used by researchers but it is inadequate in this case. The data are assumed to come from **normal distributions** with unknown, but equal, variances. Since the normality of the distribution cannot be proven for E6 population that has only 2 measurements; a ttest is not an admissible statistical test.

	E6	E8	E10	E12	E14
E6					
E8	0.6523				
E10	0.5961	0.7143			
E12	0.0053	0.0635	0.1385		
E14	0.0057	0.1810	0.3312	0.9307	

An incorrect statistical test provides a hopeful result that perhaps E6-E12 and E6-E14 pairs can be proven to be significantly different if more data is collected and normality of the distribution is proven. I will leave it up to the authors to decide. These are my suggestions: fill in a few more values and re-run statistical tests.

**Current Sample**	**Desired Sample**
E6: (212, 102)	E6: (212, 102, X3, X4, X5, X6)
E8: (21, 31, 199, 212)	E8: (21, 31, 199, 212, Y5, Y6)

E10: (10, 19, 21, 31, 219, 308)	E10: (10, 19, 21, 31, 219, 308)
E12: (8, 10, 15, 16, 32);	E12: (8, 10, 15, 16, 32);
E14: (2, 3, 4, 31, 35, 64)	E14: (2, 3, 4, 31, 35, 64)

This is the point of the review where one can still decide to ignore my analysis and suggestion and accept the story as is with no statistical basis for the underlying hypotheses. However, is proving statistical significance of the manuscript is important to the ACS Publications, I recommend reading the remainder of this review. The next pages simply elaborate on the 7 other concerns summarized on the first page.

A summary of the analysis from the remaining pages:

By adding 3-5 additional measurements to E6 and E8 samples, the authors can test whether their story is based on statistically significant observations. Without such data, the manuscript is based on observations that are not distinguishable from "null hypothesis". I encourage the authors not to perform nomination of additional peptides from an exhausted Round 7 of E6 an E8 libraries. Instead, I advise looking at earlier round (perhaps R3, R4) and making 3-5 nominations for synthesis and measurement from there.

1. The authors must cite prior work that looked at the problem that they are looking.

RE: "*We appreciate if the reviewer knew such reports, please point the report(s) and explain what exact reason s/he thinks lacking the novelty of our work*."

All three reviewers provided ample references proving existence of such work in public domain. I stand by the suggestions I made already. I mention a few new references below; most of them can be integrated in one way or another. The work is novel in its disclosure of mRNA-display-derived macrocycles of specific cyclic architecture for a specific target (MET). Overall, it is not novel in asking "how does diversity of library influences the success of the discovery". If the authors want to defend the local novelty, they are correct. The global novelty implies that they contribute to a larger field of molecular discovery and prior experience from protein phage display (Boyer et al. PNAS, 2016, 113, 3482), RNA aptamers by Szhostack (JACS 2004, 126, 5130-5137), small molecules (Tanaka et al., J. Theor. Bio. 2009, 261, 260) or fundamental insight from olfactory receptors (Lancet et al., PNAS, (1993) 90, 37153719). I recommend to discuss and juxtapose these papers to the findings of this publication.

Here is an expert opinion of Prof. David Spring (Cambridge): Galloway & Spring (2009). *Expert Opinion on Drug Discovery*, 4(5), 467–472 reflecting on similar problem in a different molecular space:

By the late 1980s, a strong belief had emerged in the pharmaceutical industry that drug discovery was simply a numbers game [1]. The development of combinatorial chemistry strategies allowed companies to generate libraries of hundreds of thousands of different compounds in a rapid manner at comparably low cost [2]. The assumption was that a multitude of drug leads would emerge simply as a consequence of the sheer volume of molecules available for screening. However, the expected surge in productivity has not materialised [3]. This disappointing degree of success is generally attributed to defects in the nature of the libraries produced, which have been described as being intrinsically useless for drug discovery [4]. Indeed, a general consensus has emerged over the past decade that ***LIBRARY SIZE IS NOT EVERYTHING****; library diversity, in terms of molecular structure and, more importantly function, is a crucial consideration [5-7].*

The authors are encouraged to read the remainder of the "Expert Opinion" and recalibrate their views to more appropriately worded scientific conclusion. Here is a well, calibrated summary that captures the most important message of this paper:

"Extreme affinity of macrocyclic peptides for MET protein discovered by mRNA-display scale by factor of <100 as the library size increases by factor 100 million."

2. The authors should discuss the difference between "size" and "diversity" in combinatorial exploration of molecular space. Again, citing prior 30 years of work from the scientists who have spent their lifetime pondering on the importance of such distinction is important.

3. The authors must not generalize their local n-of-1 case.

The problem starts from the over-general title and continued in the Abstract, which still does not mention the nature of the target and tends to generalize it as "ectodomain of receptor tyrosine

kinase”. The implication here is that 100s of other RTK ectodomain will behave the same and I disagree.

Above I suggested a summary or a title that captures the most important observation of this paper: the authors confirm, again, a well-understood log compression of cost vs. value in a local screening case against one protein.

It is yet another confirmation of extreme value theory by Tanaka *et al.* (see paper cited by reviewer 1) which stipulates that **“that the logarithm of the mean of the highest affinity in a combinatorial library grows linearly with the square root of the log of the library size”**. I plotted the data of the Authors in Microsoft Excel using coordinate system X: square root of Log(library size) vs. Y: Log(affinity) suggested by Tanaka et al. The dependency has slope k=1.1 for extreme Kd and k=0.9 for all Kd. It remarkably closely mirrors the extreme value theory of Tanaka which predicts k=1.

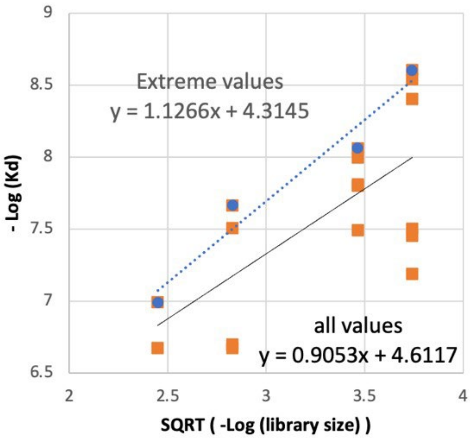

I think the authors should use these coordinates and comment on apparent alignment with extreme value theory of Takaka *et al*. The figure S9 plotted by the authors in response to Reviewer 1 is “ok” but it does not capture this conclusion because it does not use square-root-of-logarithm in its X-axis.

4. The authors need to discuss the reproducibility of their findings in their Manuscript.

Here is an example of professional, in-depth discussion of the reproducibility of selection from a publication that tests the same hypothesis as the authors.

Reproducibility and Specificity. Several observations based on the frequencies and amino acid patterns of the sequences in populations under selection validate our experimental approach. (*i*) Screening the same library against the same target in separate experiments yields reproducible frequencies f_i^t at the last round $t=3$ (*SI Appendix*, Fig. S3). (*ii*) Screening the same library against different targets yields target-specific amino acid patterns (*SI Appendix*, Fig. S4). (*iii*) Screening two libraries against the same target yields library-specific amino acid patterns (*SI Appendix*, Fig. S4). Taken together, these results show that enrichment of some of the sequences is reproducible and that it arises from selection for specific binding to the targets.

We note that one feature of our experiments is critical for reproducibility: the initial populations have a large degeneracy (the number of copies of each sequence) and not just a large diversity (the number of distinct sequences). For a sequence i with probability s_i^0 to pass a round of selection to be reproducibly selected, its number n_i^0 of copies in the initial population must, indeed, be large compared with $1/s_i^0$; if instead, $n_i^0 \sim 1/s_i^0$, the sequence will be lost in $\sim 1/3$ of the experiments. The initial degeneracy, thus, controls the range of selectivities that we can reliably infer.

Hierarchy and extremes in selections from pools of randomized proteins

Sébastien Boyer[a], Dipanwita Biswas[a,1], Ananda Kumar Soshee[a,1], Natale Scaramozzino[a,1], Clément Nizak[b], and Olivier Rivoire[a,2]

Significance

Evolution by natural selection requires populations to be sufficiently diverse, but merely counting the number of different individuals provides a poor indication of the potential of a population to satisfy a new selective constraint. To achieve a more relevant characterization of this selective potential, we performed in vitro experiments of selection with populations of partially randomized proteins and analyzed the results quantitatively by high-throughput sequencing. We find that selective potentials in these populations follow simple statistical laws, which can be interpreted with extreme value theory (the mathematical theory of extreme events—here, the rare finding of a protein meeting the selective constraints). Our results provide an approach to quantitatively measure the selective potential of a population.

To authors credit, proof of "**<u>reproducibility of discovery already existed in their study</u>**". Although 5 libraries were not designed to contain an inclusive set of peptides, identical peptide sequences reproducibly emerged from E6-E14 selections. See cursory note in **Table 1**

†**Same sequence:** [a]6-1 and 8-4; [b]8-1 and 10-2; [c]8-2 and 10-3; [d]10-1 and 12-3.

Per my request, the authors shared additional NGS data, which made it possible for me to look deeper at the question of reproducibility. In doing so, I uncovered a wonderful example of convergent discovery.

E6	E8	E10	E12	E14
YMLFWSAFESNKWNAIC	YWYYTFDGRWKEYGAAC	YYSWSGSVSESVKWNSC	YISWFAYASKDWFVRPC	YWVYGFGKLNNDWRAFC
YSWYLNWQAQWKKFEFC	YYHWNGRDGDKVEWVVC	YWYYTFDGRWKEYGAAC	YYSWSGNVSESVKWNSC	YYSWSGKDVDSVIWNNC
KVLFWSAFESNKWNAIC	KVLFWSAFESNKWNAIC	YYHWNGRDGDKVEWVVC	YSCYWVYCQKVRPDGVC	YLSWSEFDSKNWSLAYC
YFYYNWTNVAKWAKTLC	YSWYLNWQAQWKKFEFC	YIEWNRFNSKLWNCNCC	YYSWSGSVSESVKWNSC	YYSWTGRDFDAVKWNKC
YYMTYQQNAYIPRHLLC	YWYYTFDGRWEEYGAAC	YLLWHKYDNKDWVYVDC	YNCLWVECKIVLPNGRC	YLSWKYYDSSDWVILSC
TVLFWSAFESNKWNAIC	YWYYTFDGRWEEYDAAC	YWWYYWYRDEYLWRTRC	YLSWNDYNSDKWFYTPC	YIEWYVYDSKSWVYVRC
KVLFWSDFESNKWNAIC	YNSMFVYGLDDWKKYTC	YYSWSGSVSESVKWNNC	YLVWHEYDSKKWYFSLC	YYFWSGRDSDPVRWQNC
YYIIYHAPLGWIKYLSC	KVLFWSAFESNKWTAIC	YYSWSGNVSESVKWNSC	YLFWKEFNSAQWVVVKC	YNCCFIWNITNWLLVPC
YRYFFVIRNRLVLTFHC	YWYYTFDRRWEEYGAAC	KVLFWSAFESNKWTAIC	YYSWSGNVYEDVNWKEC	YIGWNRYDSREWFLLDC
YMLFWSAFESNKWTAIC	YFIRNGWRHTEITSMCC	YYILNSYPTFTVWNFRC	YFYLHCINECEWKRQPC	YYVWSGKDSDIVIWIVC
YFSMKISKSGNLLWKQC	YMLFWSAFESNKWNAIC	YLDWMTFDSKDWLVNDC	YWWYKCISECVWSWTKC	YLTWDDFDSNEWYITSC
IMLFWSAFESNKWNAIC	TVLFWSAFESNKWNAIC	YTYLSPSPFHSVWDYEC	YLEWSTYESSLWNFVMC	YIRWLTFDSKDWNFVNC
YKIWVFVNVRIFVLHQC	KVLFWSAFESNKWIAIC	YVYIGEKPAFEIIAYSC	YWYYTFDRRWVPFC	YIEWANYNSKMWKYIRC
YKVYVSKTWNFVVIITC	YYYYDYTCNYVWLEDLC	YYSWSGSVSESVKWNRC	YISWADYDSLNWYITRC	YIAWNRWNSKDWFYVDC
YFVVNNITGLVITLIKC	YWYYTFDGRWKKYTC	YYYWSGRDPDPVQYENC	YIGWKRFDSQEWYITNC	YLRWNAFNSKNWFVDAC
KVLFWSAFESNKWIAIC	YWFYNIVQCCSWYYFKC	YYSWSGRVSESVKWNSC	YRFFYYWFRGKYVKLAC	YYRWNGKLDSDIEWIGC
YMLFWSAFESNKWIAIC	YWYYTFDGRWEEYCAAC	YWYYTFNQIWIELKSHC	YWILHIDGYSWKNYAC	YLQWNNYDSRDWSIRPC
YYMTYQQNAYIPRDLLC	YMLFWSAFESNKWTAIC	YYSWSGSVSESVKWNTC	YIHVYGKPEFIVQQYRC	YYSWSGNVSESVKWNSC
YLVIIRSYSTWTIFKQC	YWYYRFDGRWEEYGAAC	YWWYYWYRDEYLWKTRC	YIKWTAYDSKQWFFVQC	YITWTDWDSSDWYFVNC
YIIKI(Stop)ATTALWI	KVLFWSAFESNKWKAIC	YIFWLVYDSPNWQILNC	YYFYYGLSGWVPLGRYC	YLNWKTWDSKSWIYVNC

By analysing the top 20 most abundant sequence across five selections (E6-E14) from new **Table S3**, I found a total of 11 reproducibly discovered sequences (highlighted in the same color).

Specifically, E10, E12 and E14 pools find the same sequence YYSWSGNVSESVKWNSC. This sequence is a confirmed binder with Kd=8.67, see E-12, Table 1), and this sequence can be dubbed 10-8 or 12-2 or 14-18 (i.e., sequence ranked 8, 2 and 14 in E8, E12 and E14 screen). This 10-8 peptide that evaded attention of the authors is now the strongest confirmed binder identified in E10 selection. The sequence YYSWSGNVSESVKWNSC needs to be added to the E10 segment of **Table 1** in the Main text of the manuscript

The most remarkable observation was reproducible convergence to the 6 out of 20 sequences from in E6 with the same 5 out of 20 sequences in E8 selections. E8 selection, in turn shares 3 other sequences with E10. The convergence of peptides originating from small, sampled libraries is counterintuitive to me based on simple sampling statistics and the origins of such libraries. E6 is created by sampling from E14 whereas E8 is sampled from E12 and, if I am reading the SI correctly, the E12 and E14 samples are nonoverlapping samples of very

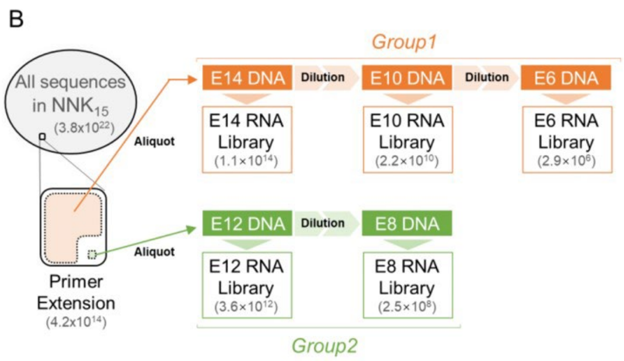

diverse pool. Based on sampling statistics that mirrors sampling dilutions, the probability of 10^6 and 10^8 sized samples from 10^{14} sized pool of 10^{22} diversity to contain 6 identical sequences is vanishingly small (the authors are encouraged to calculate this probability, I estimate the probability to find 6 identical sequences in E8 and E6 pools to be $p < 10^{-50}$).

In conclusion: The authors did not design the E6, E8, E10, E12 and E14 pools to be "nested" by design but despite divergent compositions of E12-E8 and E14-10-6 pools, the discoveries remarkably converged on the same pool of sequences. I strongly suggest for the authors to add 2-3 sentences to summarize this observation.

5. The authors perform n-of-1 selection under uniform conditions that might or might not be optimal for each library. The authors need to state this fact clearly in their manuscript. They are also encouraged to cite publications that examine the effect of selection strategy on the success of the selection (e.g., Bradbury and coworkers: "Antibodies in haystacks: how selection strategy influences the outcome of selection from molecular diversity libraries"). Ergo I am simply stating the fact: **selection under uniform conditions might or might not be optimal for each library.**

My analysis of the authors NGS data from Round 7 highlights convergence of E6, E8 and E10 libraries despite divergent initial composition of E6, E8, E10 libraries. Further analysis of data for low diversity libraries from Round 7 shows that the E6 and E8 selections are "exhausted". These libraries contain fewer than 10 unique ligands occupying 99% of the space. Among top 20 most abundant sequences 711 sequences are point mutations of the most abundant ligands. In contrast E14 selection and E12 show a balanced non-exhausted appearance with a well-represented diversity of ligands.

	E6		
parent	YMLFWSAFESNKWNAIC	56652	37.04%
	YSWYLNWQAQWKKFEFC	39966	26.13%
mutant	KVLFWSAFESNKWNAIC	39365	25.74%
	YFYYNWTNVAKWAKTLC	3065	2.00%
	YYMTYQQNAYIPRHLLC	2327	1.52%
	TVLFWSAFESNKWNAIC	1442	0.94%
mutant	KVLFWSDFESNKWNAIC	1339	0.88%
	YYIIYHAPLGWIKYLSC	791	0.52%
	YRYFFVIRNRLVLTFHC	425	0.28%
mutant	YMLFWSAFESNKWTAIC	346	0.23%
	YFSMKISKSGNLLWKQC	295	0.19%
mutant	IMLFWSAFESNKWNAIC	285	0.19%
	YKIWVFVNVRIFVLHQC	210	0.14%
	YKVYVSKTWNFVVIITC	173	0.11%
	YFVVNNITGLVITLIKC	154	0.10%
mutant	KVLFWSAFESNKWIAIC	147	0.10%
mutant	YMLFWSAFESNKWIAIC	127	0.08%
	YYMTYQQNAYIPRDLLC	124	0.08%
	YLVIIRSYSTWTIFKQC	108	0.07%
	YIIKI(Stop)ATTALWLILPC	107	0.07%

	E8		
parent	YWYYTFDGRWKEYGAAC	44745	42.40%
	YYHWNGRDGDKVEWVVC	14436	13.68%
parent	KVLFWSAFESNKWNAIC	13206	12.51%
	YSWYLNWQAQWKKFEFC	9248	8.76%
mutant	YWYYTFDGRWEEYGAAC	8364	7.93%
mutant	YWYYTFDGRWEEYDAAC	5578	5.29%
	YNSMFVYGLDDWKKYTC	1818	1.72%
mutant	KVLFWSAFESNKWTAIC	918	0.87%
mutant	YWYYTFDRRWEEYGAAC	838	0.79%
	YFIRNGWRHTEITSMCC	807	0.76%
mutant	YMLFWSAFESNKWNAIC	534	0.51%
mutant	TVLFWSAFESNKWNAIC	482	0.46%
mutant	KVLFWSAFESNKWIAIC	276	0.26%
	YYYYDYTCNYVWLEDLC	265	0.25%
	YWYYTFDGRWKKYTC	196	0.19%
	YWFYNIVQCCSWYYFKC	140	0.13%
mutant	YWYYTFDGRWEEYCAAC	137	0.13%
mutant	YMLFWSAFESNKWTAIC	126	0.12%
mutant	YWYYRFDGRWEEYGAAC	116	0.11%
mutant	KVLFWSAFESNKWKAIC	106	0.10%

E14		
YWVYGFGKLNNDWRAFC	27219	13.84%
YYSWSGKDVDSVIWNNC	25421	12.93%
YLSWSEFDSKNWSLAYC	6226	3.17%
YYSWTGRDFDAVKWNKC	6006	3.05%
YLSWKYYDSSDWVILSC	5957	3.03%
YIEWYVYDSKSWVYVRC	4070	2.07%
YYFWSGRDSDPVRWQNC	4059	2.06%
YNCCFIWNITNWLLVPC	4027	2.05%
YIGWNRYDSREWFLLDC	3490	1.77%
YYVWSGKDSDIVIWIVC	3482	1.77%
YLTWDDFDSNEWYITSC	2578	1.31%
YIRWLTFDSKDWNFVNC	2511	1.28%
YIEWANYNSKMWKYIRC	2347	1.19%
YIAWNRWNSKDWFYVDC	2184	1.11%
YLRWNAFNSKNWFVDAC	1952	0.99%
YYRWNGKLDSDIEWIGC	1874	0.95%
YLQWNNYDSRDWSIRPC	1710	0.87%
YYSWSGNVSESVKWNSC	1692	0.86%
YITWTDWDSSDWYFVNC	1672	0.85%
YLNWKTWDSKSWIYVNC	1651	0.84%

Publications from other lab deal routinely with libraries of 1 million diversity. Libraries with millionfold diversity libraries can be analyzed by routine NGS at the naïve stage with great certainly and statistical significance [1]. Furthermore, NGS can be used conclusively for selection of hits even after one round of panning [2, 3].

1. He et al. "Compositional Bias in Naïve and Chemically-modified Phage-Displayed Libraries uncovered by Paired-end Deep Sequencing ***Sci Rep***, 2018, 19, 1214
2. Ekanayake et al "Genetically Encoded Fragment-Based Discovery from Phage-Displayed Macrocyclic Libraries with

Genetically Encoded Unnatural Pharmacophores", ***J Am Chem Soc***, 2021, 143, 5497-5507

3. Yan, et al. "Learning the structure–activity relationship (SAR) of the Wittig reaction from genetically-encoded substrates", ***Chem. Sci***., 2021,12, 14301-14308

Applying exhaustive 7 rounds of selection of very small libraries with 1 million diversity is not productive. Depletion of the search pool is clear from analysis of NGS data of E6 and E8. It will become even more clear as the authors share data from earlier rounds of panning. R2-3-4-5-6 for these low diversity libraries will clearly show that earlier rounds of selection are more productive sources of analysis when compared to Round 7. If the authors decide to make 3-4 more peptides, I suggest for these hits to come from earlier rounds.

6. Conflict of interest must exist in this manuscript.

RE: "Suga and PeptiDream". I performed this internet search and in top 3 hits on Google, I found an article released this year in a premier journal of Nature Publishing Group: "Putting peptides on display" by Russell Johnson, Nature Chemical Biology, volume 20, pages1–2 (2024) https://www.nature.com/articles/s41589-023-01494-0 . It has the following abstract (copied as is):

Hiroaki Suga's research has made substantial contributions to the development of artificial ribozymes and their application in mRNA display libraries. In 2006, Professor Suga help found the biopharmaceutical company PeptiDream Inc. He spoke to Nature Chemical Biology about the future of mRNA display libraries and the advantages they offer.

The 2024 issue of Nature Chemical Biology reminded me that there exists a strong connection between Hiroaki Suga and continuing financial success of PeptiDream (and other companies founder by HS https://www.mirabiologics.com/about/). To me this is a definition of a perceived COI. I advise for the management of COI and perceived COI to be performed by the Editor and ACS publication organization. Further information can be found on ACS website:

Conflict of Interest Disclosure. During manuscript submission, ACS journal authors are required to disclose the nature of any competing and/or relevant financial interest. A statement describing any financial conflicts of

interest or lack thereof is published with each manuscript. During the submission process, the corresponding author must provide this statement on behalf of all authors of the manuscript. The statement should describe all potential sources of bias, including affiliations, funding sources, and financial or management relationships, that may constitute conflicts of interest. The statement will be published in the final article. If no conflict of interest is declared, the following statement will be published in the article: "The authors declare no competing financial interest." https://pubs.acs.org/page/policy/ethics/index.html

7. NGS data must be shared this manuscript.

The authors uploaded raw FASTQ the data to NCBI under BioProject: PRJNA1151868. The authors also provided a processed frequency summary in the SI Table 3, albeit only for Round 7. Since the paper draws conclusion about the frequencies in every round (see Main Text Figure 3), it may be more transparent to the reader to see frequencies from all 7 rounds.

7.2. My original question was **Is it possible that [repetitive round of] selection from small libraries [lead to] suboptimal selection conditions?** New data supplied by the authors confirms this suspicion. By round 7, certain peptide sequences firmly dominate the screen but they might or might not be the strongest binders.

In E10 selection, where six different peptides and selection trajectories were tested, there is no correlation between read number and affinity (3% is occupied by 20, 200 and 300 nM binders). This observation was already apparent in the original manuscript but it became even more apparent once NGS data shared by the authors in **Table S3**. The initial dominance of 20-30 nM binders (10-1 and 10-3) in Round 4 no longer exists in Round 7. By Round 7, the abundance of 200-300 nM binders (10-4 and 10-6) is indistinguishable from the abundance as 20-30 nM binders (10-1 and 10-3). This is a great illustration how transparency in data sharing and testing of large enough, statistically-significant population can lead to important scientific observations:

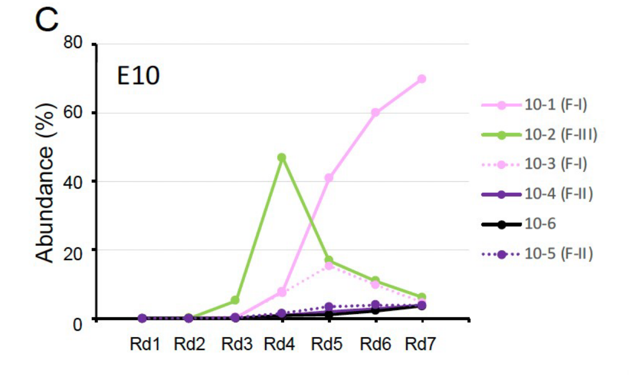

				Read number	Population
E10	10-1[d]	10.1	I	162330	69.85%
	10-2[b]	31.0	III	14156	6.09%
	10-3[c]	21.6	I	10668	4.59%
	10-4	308	II	8752	3.77%
	10-5	19.5	II	8699	3.74%
	10-6	219	-	8256	3.55%

Similar observation exists in E6 selection, where the most abundant 6-1 is weaker binder than much lesser abundant 6-2. Unfortunately, there are only 2 peptides tested and tracked from that selection, the reader is left to wonder if some optimal binders from earlier rounds have been lost. I have no doubt that additional insight into E6 selection will exist if the authors elect to test more than two peptides. Here, again, I would like to insist that Frequency data from E6 selection should exist not only for Round 7 (Table S3) but also for Rounds 6-5-4-3-2, in other words, the table needs to track back to the rounds where there is a clear pivot from all-singleton population. For library with only 1 million peptides, I expect for this pivot to happen as early as Round 2 or 3.

One of the reasons I insist on sharing pre-Round7 because I know that earlier rounds of E6 and E8 selection are likely to look like the Round7 or E14 selection. They are likely to be useful in examination of selection trajectories and overall conclusion of this manuscript.

Conclusion

I'd like to recap by re-stating requests in my first review: by adding 3-5 additional measurements to E6 and E8 samples, the authors can test whether their story is based on statistically significant observations. Without such data, the manuscript is based on observations that are not distinguishable from "null hypothesis". Statistical tests imply that all perceived differences in potency between different groups of ligands may come from the same "observation", i.e., they are not different phenomena.

I encourage the authors not to perform nomination of additional peptides from an exhausted Round 7 of E6 an E8 libraries. Instead, I advise looking at earlier round (perhaps R3, R4) and making 3-5 nominations for synthesis and measurement from there. Once data from earlier rounds is disclosed, the transparency of such nominations will also be obvious.

Author's Response to Peer Review Comments:

ACS Central Science
Senior Editor

November 20, 2024

Manuscript ID: oc-2024-01021v.R1
"Diversity sizes matter: Impact of mRNA library diversities on the discovery of macrocyclic peptides by the RaPID system"

Dear Editor,

We have revised our manuscript, "**Diversity scale of library matters: Impact of mRNA library diversity scales on the discovery of macrocyclic peptides targeting a protein by the RaPID system**". We appreciate the editor's and reviewers' valuable time and attention throughout this process. Note that the underlined words were changed to the title due to the critiques of the present reviewer.

Enclosed, please find our point-by-point responses to the reviewers' questions, including detailed responses to the newly raised concerns to further address their comments. Upon reviewing the feedback, we realized that some points required further clarification rather than changes to the manuscript itself. As such, minimal changes have been made to the manuscript in this version. The responses to Reviewer 3 are below:

> 1. The authors must cite prior work that looked at the problem that they are looking.

Response 1: This study addresses the impact of the "diversity" size or scale of library on the outcome of RaPID selection, where each library has the same library design but has a difference in its size (*which means all libraries are in the same chemical space as shown in Fig 1B*). We appreciate the references provided by Reviewer 3, however, all of them studied on the importance of the selection using libraries from different chemical spaces. Also, regarding the *Expert Opinion on Drug Discovery* paper, as Reviewer 3 said, there is a comment "*Library size is not everything*", but it should be noted that the following sentence is "*library diversity, in terms of molecular structure and, more importantly function, is a crucial consideration*". We believe that their opinion doesn't criticize our opinion that "*diversity scale of library matters*" when we use libraries with the same design and building blocks. Still, as we noted in the revised manuscript (page 5, lines 1–4), we agree that there are many other factors affecting the selection outcomes. We tried to avoid generalizing our conclusion in the previous version of the revised manuscript. However, we still believe that the knowledge developed in this study should be applicable to at least the RaPID system and possibly other platforms. We added sentences discussed this in the conclusion section.

> 2. The authors should discuss the difference between "size" and "diversity" in combinatorial exploration of molecular space.

Response 2: Both "size" and "diversity" in the previous version of the revised manuscript referred to the "number of different mRNA sequences". We are aware of the potential confusion that may arise from the use of these terms, although researchers would understand these differences in the context. However, we have decided to change all words of "size(s)" (which may include lengths and sizes) to "scale(s)" (more appropriately indicate the diversity sizes or scales which ever others use). Accordingly, we have changed the title of the manuscript as described earlier.

> 3. The authors must not generalize their local n-of-1 case.

Response 3: I believe that this reviewer, who has been working for years, knows why this is the case. Nevertheless, we added sentences in the conclusion section below:

"Although we only conducted each selection once, it is impossible to reconduct the same selection using the exact same library due to unavoidable contaminations from the previous selection. However, it should be noted that this experiment is a repeat of our previously reported work,[22] and the same family of

peptide including the critical motif for binding to MET was found in the present work, *i.e.* the experimental reproduction was clearly made. Moreover, we found new families and independent rare species. This means that the main conclusion of "diversity scale of library matters" reported herein can be generalized at least for the RaPID selection platform and possibly for other platforms."

Moreover, Fig S9 was revised according to the reviewer's suggestion, and comments on the figure was added in the revised manuscript (Page 19, lines 29-30).

> 4. The authors need to discuss the reproducibility of their findings in their Manuscript.

Response 4: Again, this is an improper comment from someone who knows the field so well as described the critique 3.

Nevertheless, we would like to clarify to the reviewer that our discussion is limited to "positive clones" whose representation exceeds a threshold of 2%, as this is consistent with our goal of assessing the impact of library size on selection outcomes. To include "non-positive clones", YYSWSGNVSESVKWNSC in Table 1, as suggested by the reviewer, is inconsistent with this objective.

Regarding the observation of the same sequences in different libraries, we have added the following sentences in the revised manuscript (Page 9, line 10-13):

"Here, it should be noted that we observed more identical sequences across groups when we extended the analysis to the top 20 sequences in each library, which might indicate that the sequence pools were nested or that mutations occurred under the selection conditions."

In addition to the above comment regarding technical difficulties of reproducing any finding of selection, of which this reviewer should be aware. Once the selection was done using a library with serial dilutions, i.e. different "diversity scales" of library, we cannot reproduce the experiments using the same library. This is simply because the contamination takes place regardless how seriously controlled experiments. We therefore ingeniously performed selection starting the lower diverse libraries to minimize the potential of the contamination from higher diverse libraries. However, we emphasize that we actually did repeat the experiment in this work using a different but the same design of library of 10^{12} diversity published in Nature Comm., and showed a same family of peptides could be identified using the NGS method. In this sense, we reproduced the finding by two different researchers and libraries.

> 5. The authors perform n-of-1 selection under uniform conditions that might or might not be optimal for each library.

Response 5: Regarding the concern about uniform versus optimal conditions, we believe that uniform conditions are necessary to facilitate a fair comparison between libraries, since it is difficult to define "optimal conditions". Although the reviewer may think the 7 rounds of the selection is optimal for E14 or E12, this is not guaranteed until we continue the selection for several rounds or more. We would like to emphasize the importance of round-by-round analysis rather than end-point analysis.

> 6. Conflict of interest must exist in this manuscript.

Response 6: The ACS editor has confirmed that there are no conflicts of interest for this manuscript.
Also, we have added following sentence in the revised manuscript:
"The authors declare no competing financial interests."

> 7. NGS data must be shared this manuscript

The processed NGS results have been uploaded as Supporting Information.

We sincerely appreciate the time and effort you have dedicated to handling and reviewing our manuscript. We did everything possible to improve the clarification as possible. We hope that our manuscript is now ready for publication in ACS Central Science. If you have any further questions or need additional information, please do not hesitate to directly contact with us.

Sincerely,

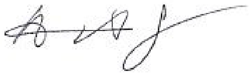

Hiroaki Suga
Professor, Department of Chemistry, Graduate School of Science
The University of Tokyo,
Tokyo, Japan

oc-2024-01021v.R3

Name: Peer Review Information for "Diversity scale of library matters: Impact of mRNA library diversity scales on the discovery of macrocyclic peptides targeting a protein by the RaPID system"

Third Round of Reviewer Comments

Reviewer: 3

Comments to the Author

This is the third time I am emphasizing that the conclusions of this manuscript are based on a skewed population (few hits from E6 and E8 were made and tested; many more binders were made and tested from E12-E14).

In this version, I was hoping to see 2-3 more peptides made and measured from E6 and E8 populations (5 total peptides max). But even after 1 year of asking for the same request, the authors refuse to make and measure mere handful of peptides. As I already shown in my previous review: E6, E8, E10, E12, E14 differences are not statistically significant. This observation remains unchanged.

In their updated conclusions the authors state "This means that the main conclusion of “diversity scale of library matters” reported herein 8 can be generalized at least for the RaPID selection."

RE: Unfortunately, this conclusion is still not statistically valid even for this publication even for this case of MET selection. As I demonstrated in my previous review, strength of populations of binders from E6 or E8 is statistically insignificant from strength of population of binders from E10, E12 or E14 ($P>0.05$ for every tested pair-wise comparison).

As a person who values statistics, I cannot recommend publishing this manuscript in a scientific journal where statistical significance of finding is important. If this manuscript is published in its current form, anyone can publish a follow up study showing that these conclusions are based on observations of low confidence ($p>0.05$). Once it happens, it will be very inconvenient for the authors and the journal to defend the significance of already published research.

I now leave it to the Editors to decide whether the top ACS journal is a suitable place to publish conclusions based on sewed samples and insignificant differences

Reviewer: 2

Comments to the Author

I was already satisfied with the first revision. I had another look at the manuscript and confirm that I recommend publication.

Author's Response to Peer Review Comments:

Hiroaki Suga, Ph.D. Professor
Bioorganic Chemistry Lab (Chemistry West Wing 2208) Department of Chemistry, Graduate School of Science
The University of Tokyo
7-3-1 Hongo, Bunkyo-ku, Tokyo, 113-8654, JAPAN hsuga@chem.s.u-tokyo.ac.jp
TEL&FAX: +81-3-5841-8372

ACS Central Science

Senior Editor

February 6, 2025

Manuscript ID: oc-2024-01021v.R1
"Diversity sizes matter: Impact of mRNA library diversities on the discovery of macrocyclic peptides by the RaPID system"

Dear Editor,

We are submitting a revised version of our manuscript entitled, "Diversity scale of library matters: Impact of mRNA library diversity scales on the discovery of macrocyclic peptides targeting a protein by the RaPID system." We greatly appreciate the thorough reviews and insightful comments from you and the reviewers throughout the revision process.

Following your recent feedback, we have performed additional analysis to robustly address concerns regarding the statistical significance of the library diversity scale. This additional work, which includes the synthesis of five additional macrocyclic peptides, binding kinetics characterization, and evaluation of the K_D difference using Mann-Whitney U tests, is now included in the Supplementary Information as Table S3, Table S5, and Figure S10.

We also added one sentence in the revised manuscript on page 19, lines 32–34 as follows:

(Upon the request from a reviewer, we intentionally picked additional peptides from E6 and E8 libraries blow our "triaged criterion", i.e. 2% of the total read counts, and chemically synthesized them; and their K_Ds were determined in Figure S10 and their statistical analysis was shown Table S5, respectively. Their SPR profiles shown in Figure S10 indicated that binding activities of these peptides from E6 and E8 libraries were evidently poorer than other peptides, suggesting that the triaged criterion used in this study was appropriate.)

We have made every effort to comprehensively address the points raised by the reviewer and have updated our manuscript and SI to reflect additional data and analyses. Even after these experiments and analyses, our conclusions regarding the impact of the mRNA library diversity scale on peptide binding properties remain unchanged. Therefore, we revised it with the same main story line, yet we believe that these changes have strengthened our manuscript and hope that it now meets the rigorous standards of ACS Central Science.

We are open to further revisions and eagerly await your decision or any additional feedback. Thank you again for patiently considering our work and for the opportunity to improve our manuscript to meet the journal's standards.

Sincerely,

Hiroaki Suga

Professor, Department of Chemistry, Graduate School of Science

The University of Tokyo,
Tokyo, Japan

oc-2024-01021v.R4

Name: Peer Review Information for "Diversity scale of library matters: Impact of mRNA library diversity scales on the discovery of macrocyclic peptides targeting a protein by the RaPID system"

Fourth Round of Reviewer Comments

Reviewer: 3

Comments to the Author

See next page

Author's Response to Peer Review Comments:

Hiroaki Suga, Ph.D. Professor
Bioorganic Chemistry Lab (Chemistry West Wing 2208) Department of Chemistry, Graduate School of Science
The University of Tokyo
7-3-1 Hongo, Bunkyo-ku, Tokyo, 113-8654, JAPAN hsuga@chem.s.u-tokyo.ac.jp
TEL&FAX: +81-3-5841-8372

ACS Central Science
Senior Editor

February 6, 2025

Manuscript ID: oc-2024-01021v.R1
"Diversity sizes matter: Impact of mRNA library diversities on the discovery of macrocyclic peptides by the RaPID system"

Dear Editor,

We appreciate the opportunity to submit the revised version of our manuscript, "Diversity Sizes Matter: Impact of mRNA Library Diversities on the Discovery of Macrocyclic Peptides by the RaPID System", for further consideration in ACS Central Science. We have carefully addressed Reviewer 3's comments and revised our manuscript accordingly. Below, we outline the key points of our revision and the rationale for our considerations.

To further evaluate the binding properties of the newly synthesized five peptides (6-3 to 6-6, 8-5) and assess their potential inclusion in the main figures and tables, we performed additional Biacore measurements. This was necessary because, with the exception of aML5, all of the newly synthesized peptides exhibited box-shaped sensorgrams in the last measurement, suggesting that their binding kinetics might vary significantly upon remeasurement.

Among these additional measurements, peptide 6-3 was the only one that showed detectable binding; however, the signal was weak, and the quality of the curve fit was poor. Also, since it did not meet our hit criterion (the "triaged criterion" is less than 2%, page 9 line 7), we decided not to include it in the manuscript but only in the statistical analysis of Table S5 with some cautionary notes.

Although peptide 8-5 met our hit criteria, the SPR signal exhibited a reproducible negative shift at high concentrations, making it impossible to accurately evaluate its binding. Therefore, it was not included in the main manuscript.

For the other newly synthesized peptides (6-4, 6-5, 6-6), none met all three essential criteria for a binding peptide—reproducibility in SPR, sufficient signal intensity, and clear concentration dependence. Consequently, we determined that none of these peptides were suitable for inclusion in the manuscript but are presented in Figure S10 of the Supporting Information.

We would like to emphasize again that these new peptides were synthesized in response to Reviewer 3's request and were not initially identified as meeting our selection criteria. Given their

poor affinity, it is possible that their initial enrichment was due to non-specific factors rather than the binding to the selection target.

Based on these results, we have decided that no additional peptides should be included into the main text. The SPR data for the newly synthesized peptides (Figure S10) and the statistical analysis in Table S5 have been updated following the remeasurement of the binding kinetics. In addition, a description of the statistical analysis has been added to the manuscript as follows (Page 19, Lines 32 – Page 20, Lines 2).

Although we could see some trend between the K_D *of the hit peptide and the diversity scale of the library as shown in Figure 2 and S9, the statistical analysis didn't show the significance using the current data set. Further analysis including the data of additional synthesized peptides is shown in Figure S10 and Table S5.*

Hiroaki Suga, Ph.D. Professor
Bioorganic Chemistry Lab (Chemistry West Wing 2208) Department of Chemistry, Graduate School of Science
The University of Tokyo
7-3-1 Hongo, Bunkyo-ku, Tokyo, 113-8654, JAPAN hsuga@chem.s.u-tokyo.ac.jp
TEL&FAX: +81-3-5841-8372

We believe that our revised manuscript provides a more robust and scientifically sound analysis, and we respectfully submit it for your consideration.

We appreciate your patience throughout this review process and your efforts to improve our manuscript. We remain open to further constructive revisions and look forward to your decision or any additional feedback.

Sincerely,

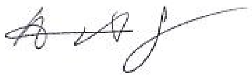

Hiroaki Suga, Professor
Department of Chemistry, Graduate School of Science
The University of Tokyo,
Tokyo, Japan
